# Breaking the scaling relations of effective CO_2_ electrochemical reduction in diatomic catalysts by adjusting the flow direction of intermediate structures†

**DOI:** 10.1039/d4sc03085k

**Published:** 2024-08-08

**Authors:** Yanwen Zhang, Zhaoqun Yao, YiMing Yang, Xingwu Zhai, Feng Zhang, Zhirong Guo, Xinghuan Liu, Bin Yang, Yunxia Liang, Guixian Ge, Xin Jia

**Affiliations:** a School of Chemistry and Chemical Engineering, State Key Laboratory Incubation Base for Green Processing of Chemical Engineering, Shihezi University Shihezi 832003 China jiaxin@shzu.edu.cn; b Department of Physics, College of Science, Shihezi University Shihezi 832003 China geguixian@126.com; c College of Agriculture, Shihezi University Shihezi 832003 China yaozhaoqun@sina.com; d Key Hefei National Laboratory for Physical Sciences at the Microscale, School of Chemistry and Materials Science, University of Science and Technology of China Hefei Anhui 230026 China; e Department of Mathematics, College of Science, Shihezi University Shihezi 832003 China

## Abstract

The electrocatalytic carbon dioxide reduction reaction (CO_2_RR) is a promising approach to achieving a sustainable carbon cycle. Recently, diatomic catalysts (DACs) have demonstrated advantages in the CO_2_RR due to their complex and flexible active sites. However, our understanding of how DACs break the scaling relationship remains insufficient. Here, we investigate the CO_2_RR of 465 kinds of graphene-based DACs (M1M2-N6@Gra) formed from 30 metal atoms through high-throughput density functional theory (DFT) calculations. We find that the intermediates *COOH, *CO, and *CHO have multiple adsorption states, with 11 structural flow directions from *CO to *CHO. Four of these structural flow directions have catalysts that can break the linear scale relationship. Based on the adsorption energy relationship between *COOH, *CHO and *CO, we propose the concepts of linear scaling, moderate breaking, and severe deviation regions, leading to the establishment of new descriptors that identify 14 catalysts with potential superior performance. Among them, ZnRu-N6@Gra and CrNi-N6@Gra can reduce CO_2_ to CH_4_ at a low limiting potential. We also discovered that DACs have independent bidirectional electron transfer channels during the adsorption and activation of CO_2_, which can significantly improve the flexibility and efficiency of regulating the electronic structure. Furthermore, through machine learning (ML) analysis, we identify electronegativity, atomic number, and d electron count as key determinants of catalyst stability. This work provides new insights into the understanding of the DAC catalytic mechanism, as well as the design and screening of catalysts.

## Abstract 2

High-throughput calculations show 11 flow directions from *CO to *CHO on DACs. The catalyst disrupting the linear adsorption relationship is linked to multiple, not just one, flow direction.

## Graphical abstract

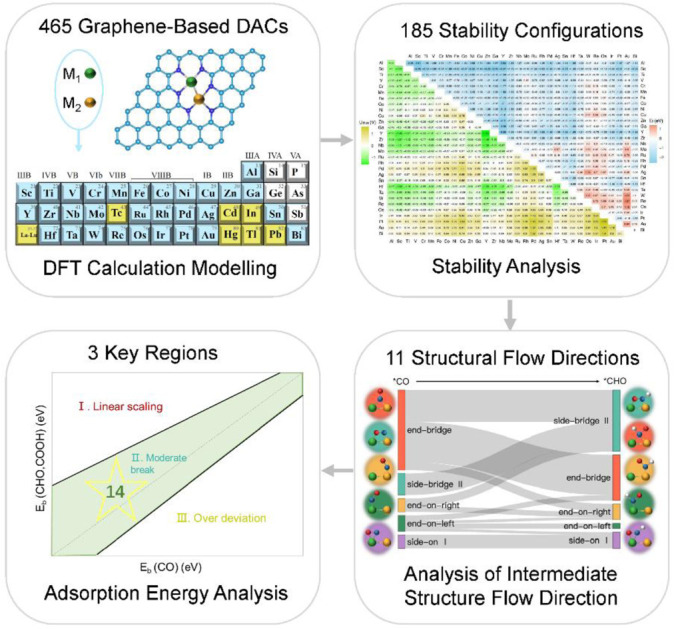

## Introduction

**Fig. 1:**
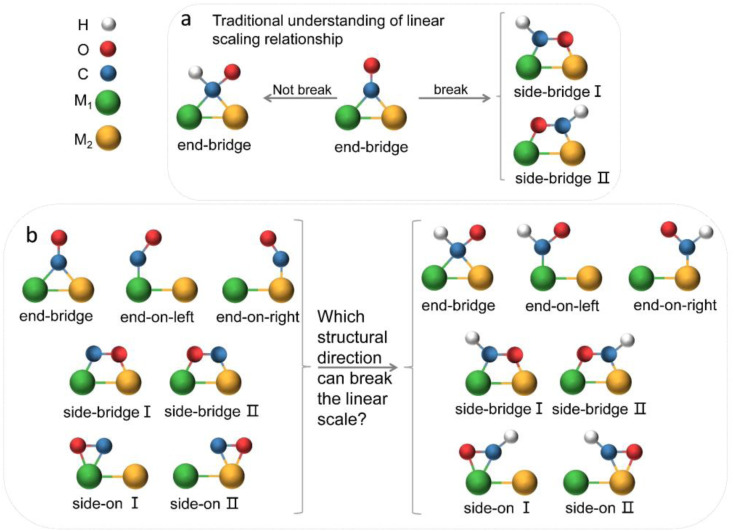
(a) Traditional understanding of breaking the linear scaling relationship between *CO and *CHO intermediates on the surface of bimetallic catalysts. (b) Various adsorption configurations of *CO and *CHO, posing significant challenges in decoupling the adsorption energies. Green and orange represent two metal atoms, blue for carbon atoms, red for oxygen atoms, and white for hydrogen atoms.

The electrochemical reduction of CO_2_ into useful chemicals or fuels using intermittent green energy sources is a promising strategy for reducing CO_2_ emissions and mitigating climate change.^1–3^ In recent decades, alongside experimental efforts, there has been inspiring and remarkable progress in theoretical understanding.^4,5^ Theoretical calculations play a crucial role in guiding experiments, shedding light on active sites and reaction mechanisms at the atomic scale, and offering insights into the design and optimization of efficient electrocatalysts.^6,7^ The emergence of DACs, as an extension of single-atom catalysts (SACs), has recently drawn widespread interest.^8–10^ DACs, with higher metal loadings and more complex and flexible active sites compared to SACs, achieve better catalytic performance, presenting more opportunities for electrocatalysis.^11–16^ However, we still lack a deep understanding of the reaction mechanism of diatomic catalysts in the complex process of carbon dioxide reduction and multi-electron transfer. The linear scaling relationship has always provided a direct principle for the design of electrocatalysts.^17–20^ Initially, Nørskov and colleague discovered adsorption energy scaling relations based on the adsorption structure characteristics of intermediates on metal surfaces, simplifying complex multidimensional analysis to two dimensions, and successfully predicted that the Cu (211) surface had favorable capabilities for catalyzing the reduction of CO_2_ to methane.^21^ The discovery of scaling relationships has strongly guided experimental work but also implied that the reduction efficiency of traditional transition metal catalysts is limited by inherent scaling relationships (SRLs). In order to break the linear scaling relationship, they also theoretically proposed a method to stabilize key intermediates through the synergistic effect of multi-site and ligand stabilization, so as to achieve the purpose of reducing the free energy of the decision step reaction.^22,23^ The ordered AuCu nanoparticles synthesized by Kim in the experiment can selectively convert CO_2_ into CO with a Faraday efficiency of 80%.^24^ Ma and colleagues utilized synthesized Cu–Pd bimetallic alloy catalysts for the CO_2_RR, finding that ordered CuPd catalysts exhibited the highest selectivity for C_1_ products, suggesting that geometric rather than electronic effects were key to the selectivity of bimetallic Cu–Pd catalysts.^25^ Obviously, multi-site insights are beneficial for the design of catalytic surfaces, thereby further improving the activity and selectivity of CO_2_ reduction. This design concept was subsequently introduced into diatomic catalysts. Li *et al.* constructed diatomic catalysts (Cu_2_, CuMn and CuNi) on graphene substrates, finding that the MnCu@2SV catalyst was selective for CH_4_ production, while NiCu@2SV facilitated CH_3_OH production due to the difference in oxygen affinity between the incorporated Mn and Ni.^26^ Wang *et al.* applied the metal alloy double-site strategy to SACs, studying 21 kinds of diatomic catalysts. They found that the dual sites provided by DACs facilitated a side-bridging adsorption of *CHO, enhancing the adsorption strength and decreasing the Gibbs free energy difference between *CO and *CHO, thus reducing the reaction overpotential.^27^ The geometric structure and flow direction of *CO end adsorption to dual-bridged *CHO were deemed essential for breaking the linear scaling relationship of key intermediates in DACs, and this conclusion has been widely accepted (Fig. 1a). The above theoretical studies have deepened people's understanding of diatomic catalysts and promoted the development of experiments. Many recent experiments have also confirmed that dual sites generally have better selectivity and activity towards the CO_2_RR than corresponding unit sites.^28–32^

However, in the CO_2_RR process, a large number of experiments have shown that its key intermediates have multiple possible adsorption structures (Fig. 1b), which greatly affect catalytic performance. It is believed that the identification of adsorption states is crucial for improving catalyst design and optimizing the structure–reactivity relationship.^33–36^ Presently, theoretical studies of dual-atom systems often involve calculations on a limited selection of metals, resulting in conclusions that only present partial adsorption states and may not correspond with experimental findings. Therefore, it is necessary to expand the range of candidates in theoretical calculations to provide a necessary sample of adsorption states and establish a more accurate relationship between catalytic performance and intermediate adsorption states. In fact, the introduction of double sites provides opportunities for CO_2_RR research, but also makes the catalytic mechanism more complex.

In recent years, rapid advances in computational science and technology, augmented computational power, and the application of machine learning in catalyst design and screening have expanded our research horizons and allowed us to gain experience in a wider range of catalyst systems.^37–40^ In this study, we employ our self-developed high-throughput computational program to conduct DFT simulations on 465 candidate materials formed from 30 types of metal atom combinations. The results indicate that there is a linear relationship between the adsorption energies of *COOH and *CHO, while there is a significant deviation between them and *CO. There are three distinct regions in the adsorption energy relationship diagram: linear scaling region I, moderately broken region II, and excessively deviated region III. Only candidates within region II potentially possess outstanding catalytic performance. Further analysis of structural flow revealed that no specific structural flow would break the linear scaling relationship between key intermediates. This work, leveraging high-throughput computational screening, has significantly expanded the pool of study subjects, refined the adsorption structures of intermediates, and provided new insights into breaking the linear scaling relationships of dual-atom catalysts in the CO_2_RR. Our research findings provide guidance for the rational design and experimental synthesis of diatomic catalysts.

## Results and discussion

In experiments, bimetallic catalysts have been successfully anchored on various two-dimensional materials such as graphene, graphitic carbon nitride (gC_3_N_4_), rectangular expanded phthalocyanines, and nitrogen-doped carbon, forming metal–carbon/nitrogen dimer structures coordinated by C or N atoms.^41–46^ These bimetallic catalysts, typically formed under high-temperature conditions in an N_2_ atmosphere through nitrogen coordination, not only enhance the binding strength between the catalyst and the substrate but also improve the stability of the catalysts. A common experimental structure is the graphene-based bimetallic catalyst with four vacancies, where two metal atoms are usually stabilized by six pyridinic nitrogen atoms.^11,47,48^

**Fig. 2:**
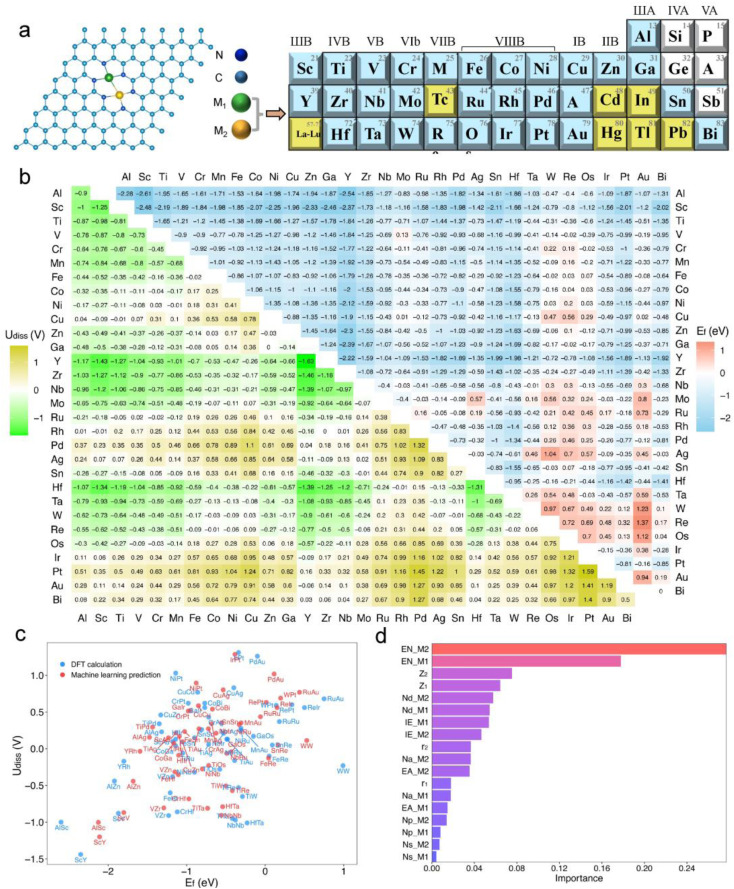
(a) Structure of the graphene-based bimetallic catalyst (M1M2-N6@Gra). (b) Calculated formation energy (*E*_f_, in eV) and dissolution potential (*U*_diss_, in V) of metal atoms in M1M2-N6@Gra. (c) Machine learning predictions of *U*_diss_ and *E*_f_ compared with DFT calculations. (d) Feature importance analysis based on a random forest model algorithm in machine learning.

In this study, we have developed a library of graphene-based bimetallic catalysts, denoted as M1M2-N6@Gra (Fig. 2a). Thirty metal atoms were selected, including 26 transition metals (excluding lanthanides, Tc, Cd, and Hg) and four main-group metals (Al, Ga, Sn, and Bi), to serve as the central atoms in the graphene's four vacancies. Toxic and radioactive elements such as Tc, Cd, Hg, In, Tl, and Pb were excluded to ensure the safety and practicality of the catalysts.

Theoretical studies were conducted on these M1M2-N6@Gra structures composed of 30 elements, and their geometric structures were optimized (Table S1†). In the optimized structures, whether the bimetallic catalysts lie within or protrude from the graphene plane primarily depends on the radii of the two metal atoms. For example, diatomic catalysts formed by atoms with larger radii (such as Sc, Y, Hf, *etc.*) will protrude from the graphene plane, forming a buckling structure, while diatomic catalysts formed by atoms with smaller radii (such as Fe, Co, Ni, *etc.*) are almost entirely located within the graphene plane. In the optimized M1M2-N6@Gra structures, the average bond lengths between the metal centers and the coordinating nitrogen atoms range from 1.91 Å to 2.57 Å, while the distances between the two metal atoms vary from 1.77 Å to 3.23 Å. These structures exhibit an adaptive interconnection between the two metal atoms and the adjacent three nitrogen atoms, which not only lowers the system's energy to stabilize the structure but also cooperatively controls the adsorption of intermediates through geometric adjustments and electron transfer. This results in a catalytic efficacy that differs from single-atom catalysts in terms of catalyst stability, adsorption structure, and electronic structure.^49^

To evaluate the thermodynamic and electrochemical stability of M1M2-N6@Gra dual-atom catalysts composed of 30 elements, we employed formation energy (*E*_f_) and dissolution potential (*U*_diss_) as metrics. The equations are defined as follows:^50,51^

1 *E*_f_ = (*E*_M1M2-N6@Gra_ − *E*_N6@Gra_ − *E*_M1_ − *E*_M2_)/2

2
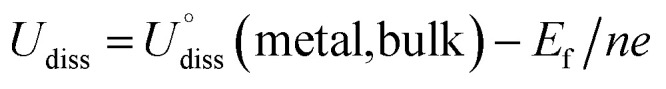
where *E*_M1M2-N6_@Gra and *E*_N6_@Gra are the energies of DACs and the metal-free graphene-based structure calculated by DFT, respectively. *E*_M1_ and *E*_M2_ represent the energies of metal atoms in their most stable bulk structures. 
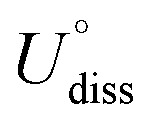
 (metal, body) and *n* are the standard dissolution potential of the body metal and the number of electrons involved in dissolution, respectively. By our definition, an *E*_f_ value of less than 0 eV indicates thermodynamic stability, while a *U*_diss_ value greater than 0 V *vs.* SHE suggests electrochemical stability. The exact values of *E*_f_ and *U*_diss_ are listed in Table S2.†

As illustrated in Fig. 2a, dual-atom catalysts formed by elements with lower atomic numbers exhibit greater thermodynamic stability. For instance, the formation energies for the third-period elements are all negative, indicating high thermodynamic stability. With increasing atomic numbers, the formation energies of the fourth and fifth-period elements tend to be positive, suggesting decreased thermodynamic stability.

Regarding electrochemical stability, we found that some diatomic catalysts of certain elements in the third, fourth, and fifth periods exhibit poor electrochemical stability due to their mostly negative dissolution potentials (Fig. 2a), especially the diatomic catalysts formed between the atoms of elements in the IIB to VIIB groups. Conversely, dual-atom catalysts composed of VIII group elements like Fe, Co, Ni, and Cu show greater electrochemical stability. Furthermore, electrochemical stability may correlate with atomic electronegativity. The dissolution potential of diatomic catalysts formed by atoms with lower electronegativity tends to be negative, whereas DACs composed of atoms with higher electronegativity are the opposite.

Out of the 465 objects considered, 386 exhibit *E*_f_ values below zero, many of which are significantly negative, indicating high thermodynamic stability.^20,52,53^ Regarding *U*_diss_, 237 systems show positive values, demonstrating electrochemical stability under acidic conditions. After comprehensive consideration, we have identified 185 dual-atom catalysts meeting the stability criteria for further investigation.

Additionally, we employed a machine learning (ML) model to study the correlation between catalyst stability and their intrinsic properties.^20,52^ The model included 26 intrinsic descriptors of catalysts, such as atomic number, atomic radius, bond length between metal atoms and neighboring nitrogen atoms, bond length between metal atoms, valence electron count, electronegativity, electron affinity, and first ionization energy (Tables S1 and S3†). A Pearson correlation analysis was conducted to reduce the number of variables, setting the number of trees and maximum depth to 200 and 5, respectively, with a random state of 9. After standardizing the data, a random forest regression model was established. Encouragingly, the trained model showed satisfactory performance in predicting the stability of dual-atom catalysts compared to DFT calculations (Fig. 2c), with training and testing *R*^2^ scores of 0.94 and 0.97, respectively. The feature importance analysis, as shown in Fig. 2d, revealed that the electronegativity (EN), atomic number (*Z*), and d-electron numbers (*N*_d_) of metal atoms are the main factors affecting the stability of the catalysts, with electronegativity (EN) having the greatest impact.

In summary, through theoretical calculations and machine learning approaches, we have comprehensively assessed the stability of M1M2-N6@Gra dual-atom catalysts, successfully identifying 185 catalyst systems that meet stability requirements, thus providing a solid foundation for future research.

**Fig. 3:**
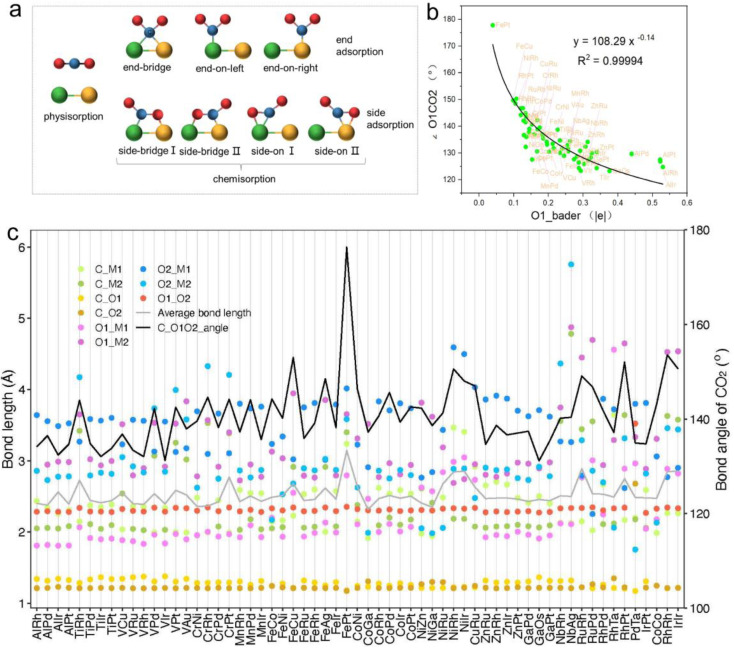
(a) Potential adsorption configurations of carbon dioxide at active sites of catalysts, including both physisorption and chemisorption. Chemisorption shows three terminal and four lateral adsorptions. Green and orange represent two metal atoms, blue for carbon and red for oxygen atoms. (b) Correlation curve between differential charge of oxygen atoms bonded to metal and the CO_2_ activation angle. (c) Bond lengths, CO_2_ activation angles, average bond lengths (for bonds ≤3.5 Å), and bond lengths within CO_2_ between carbon and the two oxygen atoms during CO_2_ adsorption on bimetallic catalysts.

The adsorption and initial activation of CO_2_ are key steps in the CO_2_RR and often determine the CO_2_ reduction efficiency of the catalyst.^54,55^ In this study, we evaluated the adsorption and activation degree of CO_2_ by comparing the physicochemical properties of CO_2_ molecules before and after adsorption and activation, such as changes in bond length and bond angle. Despite the clear affinity of individual metals for carbon (C) and oxygen (O) on metal crystal surfaces, the affinity may alter when metals are combined into dimers, particularly for metal atoms with similar affinities. Hence, when constructing the initial structure of CO_2_ adsorption, CO_2_ molecules were placed horizontally above the adsorption plane, and C atoms were located above the center of the connection between two metal atoms, and two O atoms were distributed on both sides, so as to achieve structural optimization under the adsorption competition between C and O atoms, and achieve a stable adsorption configuration (Fig. 3a and S1†). Ultimately, 61 M1M2-N6@Gra systems interacted with CO_2_ molecules *via* chemisorption, with adsorption energies less than 0 eV (Table S4†).

In order to further explore the mechanism of CO_2_ activation by diatomic catalysts, we analyzed the changes in bond angles and bond lengths of CO_2_ molecules on the catalyst surface (Fig. 3c and Table S5†). Maximum bending was observed when C and O atoms were adsorbed on two metal atoms in side-bridge mode, indicating the highest level of activation. When the C and O atoms are attached to the same metal atoms in side-on terminal mode, the maximum bending is observed, indicating the highest level of activation. When C and O were adsorbed on the same metal atom in end-on mode, smaller bending deformation was observed, indicating a decrease in activation level. When only C atoms are adsorbed on metal atoms, CO_2_ molecules do not bend much and have the lowest degree of activation. We extracted bond lengths less than or equal to 3.5 Å from the six possible bond lengths between carbon dioxide and bimetallic materials and calculated the average value. We found a linear relationship between the average effective bond length and the activation angle of carbon dioxide, with a Pearson correlation coefficient of 0.68, indicating a strong correlation between the two (Fig. S2†). The average effective bond length can serve as a marker of the degree of CO_2_ activation in diatomic catalysts, and indirectly reflect a maximum effective distance of 3.5 Å between the atoms in the adsorbed small molecules on the catalyst surface and the metal atoms in the active center (Fig. 3c). Detailed representations of changes in the C=O bond lengths of CO_2_ molecules on DACs are provided in Table S5.† Significantly, when CO_2_ is in a side-bridge adsorption mode, the C=O bond between the C and O atoms (O1) bonded to the metal atom is slightly elongated, ranging from 1.22 Å to 1.38 Å, which is longer than the bond length (1.21 Å) between the C atom and the distal unadsorbed O atom (O2). When CO_2_ molecules are only bonded by C atoms to metal atoms, the C=O bond lengths remained close to those in free CO_2_ molecules. These observations underscore the synchronized activation of bond angles and lengths, highlighting the synergistic effect of dual active sites in DACs for activating CO_2_ molecules. Particularly, in the PdTa system, due to the fact that Pd and Ta atoms are located on the same side of graphene and far apart, and they exhibit strong adsorption on C and O1 atoms, the C=O1 bond breaks, resulting in a significant increase in the distance between C and O1. Moreover, no apparent correlation was found between the distance of the two metal atoms in DACs and the activation angle of the CO_2_ molecule.

Intriguingly, our research also found a clear correspondence between the charge transfer from the active centers of DACs to CO_2_ molecules and the degree of CO_2_ activation. Specifically, a greater amount of charge transferred to the CO_2_ molecule corresponded to a smaller activation angle (∠O1CO_2_), indicating more pronounced activation (Fig. S3 and Table S6†). Notably, the correlation between the Bader charge of the bonded oxygen atom (O1) and the activation angle was most evident. Through nonlinear curve fitting, we obtained the equation and constant coefficient that represent this relationship, with a coefficient of determination *R*^2^ as high as 0.99 (Fig. 3b). This emphasizes the crucial role of charge transfer between DACs and CO_2_ in promoting CO_2_ activation, and it is more reasonable to use the Bader charge of O1 as an indicator to describe the degree of activation. Fig. 3b reflects a nonlinear exponential relationship between the Bader charge of O1 on the adsorbed carbon dioxide and the activation angle. As the Bader charge of O1 transfer increases, the activation angle starts to decrease from 180° and tends towards 120°. From the trend of the curve, it can be seen that as the activation angle approaches 120°, a significant amount of charge transfer is required to achieve a change in the unit activation angle, which means that there is a certain activation limit for carbon dioxide activation angle. From the perspective of adsorption structure, as the activation angle increases, O1 and O2 will become closer and closer. Meanwhile, we also noticed that O1 and O2 have the same type of charge transfer as shown in Fig. S3,† and as the activation angle decreases, they inevitably exhibit stronger repulsive interactions between them, which will prevent further activation. Therefore, from the trend of curve extension, the nonlinear relationship between Bader charge and carbon dioxide activation angle is consistent with its physical significance. The above results not only demonstrate the geometric structure of the adsorption and activation of CO_2_ molecules by diatomic catalysts, but also reveal the relationship between average effective bond length and activation angle, providing a new perspective for understanding the mechanism of DACs in CO_2_ electrochemical reduction.

**Fig. 4:**
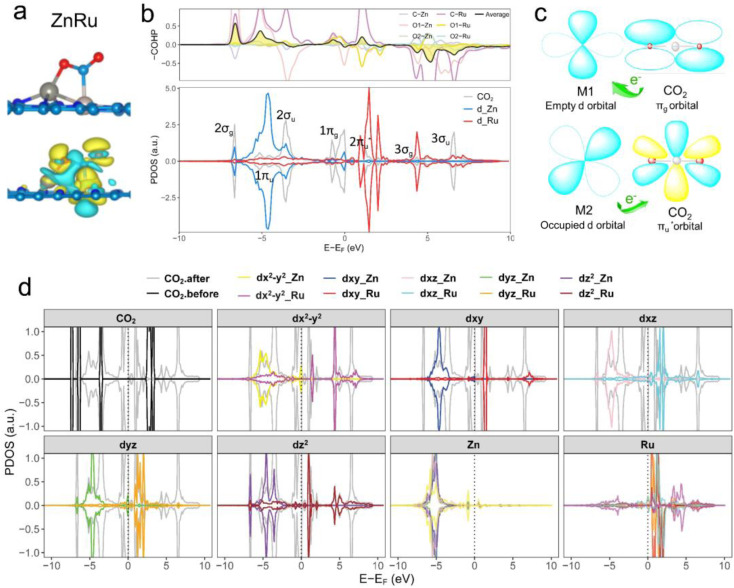
(a) Optimized adsorption configuration and charge density difference of chemisorbed CO_2_ on the ZnRu-N6@Gra surface. Charge depletion and accumulation are shown in cyan and yellow, respectively, with an isosurface value of 0.003 e Å^−3^. (b) Calculated partial density of states (PDOS) and crystal orbital Hamilton population (COHP) for CO_2_ adsorbed on ZnRu-N6@Gra. Fermi level set at 0 eV. Positive COHP indicates bonding states, negative for antibonding states; colored lines represent COHP between two metal atoms and C, O1, and O2 atoms, with black indicating average COHP. (c) “Donor–acceptor” mechanism on a dual conduit formed by the π_g_ and 
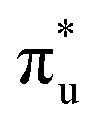
 orbitals of adsorbed CO_2_ with d orbitals of Zn and Ru. (d) PDOS calculations for Zn and Ru d orbitals in ZnRu-N6@Gra before and after CO_2_ adsorption; Fermi level at 0 eV.

Further analysis of electronic structures involved partial density of states (PDOSs) and crystal orbital Hamilton population (COHP) calculations.^56^ As shown in Fig. 4b (ZnRu), S4 and S5† (the remaining 13 types of DACs), there was evident hybridization between the d orbitals (the s and p orbitals of the main group Al atoms) of transition metal atoms and the molecular orbitals of CO_2_. COHP analysis indicated interactions between CO_2_ molecular orbitals and metal d orbitals forming partially occupied states. The average integrated COHP (ICOHP) values ranged between ∼−0.474 and −1.693, signifying strong interactions between DACs and CO_2_. The more negative the ICOHP value, the stronger the interaction between two atoms, favoring bond formation. A detailed analysis of ICOHP values in Table S7† aligned closely with adsorption structures. For instance, in ZnRu-N6@Gra, the ICOHP values for Zn with O1 and Ru with C were −1.625 and −3.399, respectively, significantly higher than those of other interactions, indicating strong bonding between Zn with O1 and Ru with C, forming a side-bridge II structure as depicted in Fig. 4a. Similarly, in VAu-N6@Gra, the V atom showed strong adsorption affinity towards O1 and C, as indicated by ICOHP values of −3.014 and −3.222, respectively, leading to an end-on-left configuration (Fig. S1†), with similar observations in VCu and TiRh. These findings demonstrate that the varied adsorption structures of CO_2_ on DACs are driven by the unique electronic structures of the dual-atom active sites.

Taking ZnRu-N6@Gra as an example, we further analyzed the molecular orbitals of carbon dioxide and the d orbitals of Zn and Ru before and after CO_2_ adsorption. The density of states (PDOS) analysis in Fig. S6† reveals a significant hybridization between the d orbitals of the two metal atoms and the CO_2_ molecular orbitals. This hybridization effect causes the CO_2_ molecular orbitals to disperse and broaden from their initially narrow and discrete density of states distribution, with a notable shift in energy levels, especially the movement of the 
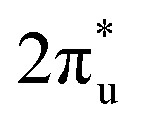
 orbital towards the Fermi level (Fig. S7a and b†). From Fig. S8,† it can be seen that although the density of states of metals before and after adsorption has shifted in energy distribution, the two metal orbitals are always distributed on both sides of the Fermi level. Among them, Zn d orbitals are mainly distributed below the Fermi level, while Ru d orbitals are mainly distributed above the Fermi level. Further PDOS calculations showed that the five degenerate orbitals of the metal d-orbitals were split into different energy states (Fig. 4d). Zn d_*xy*_, d_*xz*_ and d_*z*^2^_ orbitals were lower energy occupied states, while some of the orbitals (d_*x*^2^–*y*^2^_ and d_*yz*_) were unoccupied. Conversely, some of Ru's orbitals (d_*x*^2^–*y*^2^_ and d_*xz*_) were occupied, with the remaining three orbitals being unoccupied. This characteristic of dual-atom electronic orbitals provides a substantial potential for interactions between the metal atoms and the occupied and unoccupied orbitals of CO_2_. The COHP analysis around the Fermi level revealed two significant bonding states, one between the C 
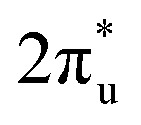
 orbital and Ru orbital, and the other between the 1 π_g_ orbitals and Zn d orbital. PDOS results indicated that CO_2_ 1π_g_ orbital donated electrons to the Zn d_*yz*_ empty orbital and Ru d_*z*^2^_ empty orbital, forming bonding states at −1.00 eV, enhancing CO_2_ adsorption. Moreover, Ru d_*xy*_, d_*xz*_, and d_*z*^2^_ orbitals as occupied states interacted strongly with the CO_2_
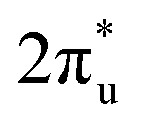
 orbital near and above the Fermi level, feeding electrons back to the 
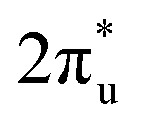
 orbital and causing it to shift towards the Fermi level, forming bonding states at 1.00 eV. Table S8† shows that the electron numbers of the two metal atoms did not change significantly before and after adsorption. They served merely as intermediaries for electron transfer through two channels near the Fermi level: the 1π_g_ and 
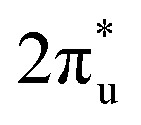
 orbitals, employing a “donor–acceptor” mechanism to facilitate electron transfer between CO_2_ and the substrate, enhancing CO_2_ adsorption and activation (Fig. 4c). The COHP analysis in Fig. 4b shows that the bonding between Zn and O1 dominates the interaction between their d orbitals and the CO_2_ 1π_g_, while the interaction between Ru and C atoms dominates the bonding between their d orbitals and CO_2_
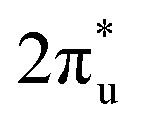
 orbitals. Fig. S9–S19 and Table S7† display the PDOS and COHP conditions for other dual-atom catalysts before and after adsorbing CO_2_, further confirming these findings.

**Fig. 5:**
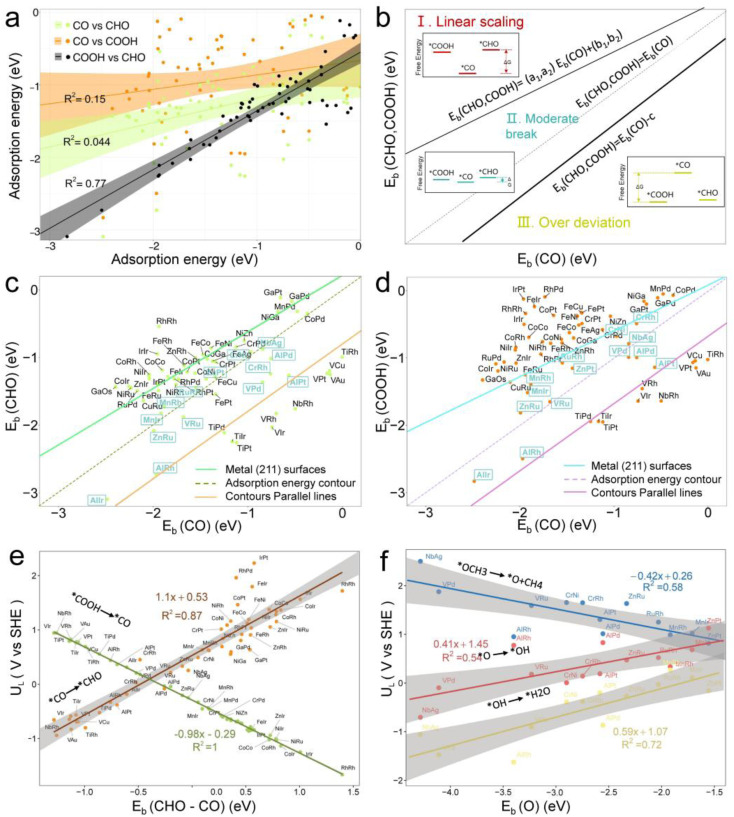
(a) Relationship between the adsorption energies of *E*_b_(CHO), *E*_b_(COOH), and *E*_b_(CO). Linear correlation exists between *E*_b_(CHO) and *E*_b_(COOH), whereas *E*_b_(CHO) and *E*_b_(COOH) show non-linear correlation with *E*_b_(CO). (b) Classification into linear scaling zone I, moderate deviation zone II, and excessive deviation zone III, based on linear scaling lines of metal (211) surfaces and lines parallel to adsorption energy isotherms. Insets show free energies of *COOH, *CHO, and *CO in the three zones. In zone I, *COOH and *CHO have higher free energies than *CO. In zone II, free energies of the three are similar, and in zone III, *CO has higher free energy than *COOH and *CHO. On M1M2-N6@Gra surfaces, (c) *E*_b_(CHO) *vs. E*_b_(CO), (d) *E*_b_(COOH) *vs. E*_b_(CO). (e) Limiting potential (*U*_L_) for the proton transfer step as a function of descriptor *E*_b_(CHO–CO). (f) As a function of descriptor *E*_b_(O). The equilibrium potential for the overall electrochemical reduction of CO_2_ to CH_4_ compared to SHE is +0.17 V.

In our study, to further understand the electrocatalytic activity of the 61 selected DACs, we optimized the structures of *COOH, *CO, and *CHO, three key intermediates in the CO_2_ reduction reaction (CO_2_RR). Except for the disruption of O–O bonds in RhTa and PdTa while adsorbing *COOH, all other intermediates were successfully optimized. We calculated the adsorption energies of these intermediates and found a linear relationship between the adsorption energies of *COOH and *CHO, whereas the relationship with *CO significantly deviated (see Fig. 5a). A linear fitting of *CHO and *COOH adsorption energies yielded a Pearson's *r* value of 0.77, suggesting a coupled adsorption energy. Linear relationships maintain close connections and correspondences between intermediates, while deviating from linear relationships can serve as clues for finding high-performance catalysts.

**Fig. 6:**
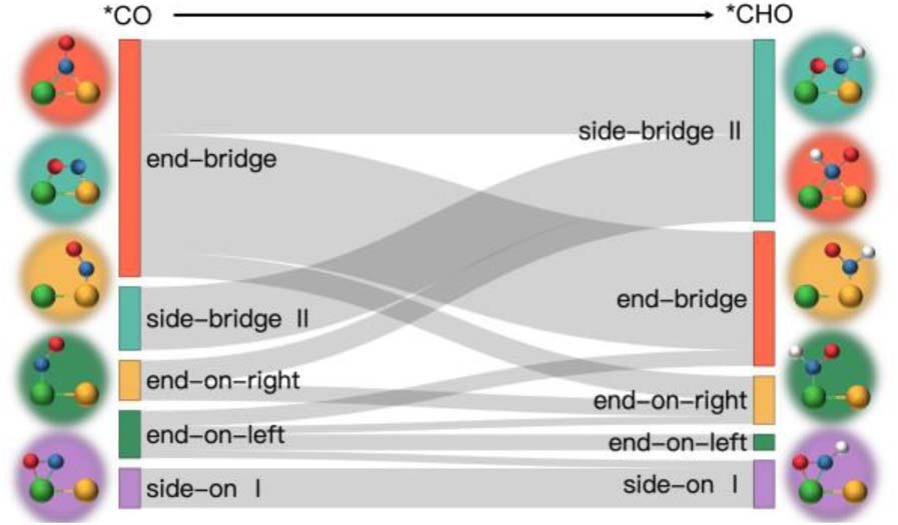
Structural transition pathway from *CO to *CHO.

Fig. 5b illustrates three regions based on the adsorption energy relationships of *COOH, *CHO, and *CO on the catalysts: region I (linear scaling relation), region II (moderate deviation), and region III (excessive deviation). The boundaries of these regions are defined by a linear scaling line from the metal (211) surface (*E*_ads_ (CHO,COOH) = (*a*_1_,*a*_2_)*E*_ads_(CO) + (*b*_1_,*b*_2_)) and a parallel line to the adsorption energy contour line (*E*_ads_(CHO,COOH) = *E*_ads_(CO)-*c*).^22^ The catalysts in the upper left corner of Fig. 5c and d (including the linear scale line) exhibit strong adsorption of *CO and weak adsorption of *COOH and *CHO, resulting in a lower Gibbs free energy of *CO relative to *COOH and *CHO. This will generate a high overpotential between *CO and *CHO, making these catalysts unsuitable as screening objects. Conversely, catalysts located in the lower right region III have significantly stronger adsorption energies for *COOH and *CHO than *CO, resulting in lower Gibbs free energies for *COOH and *CHO compared to *CO, which is also unfavorable for the CO_2_RR process. Therefore, only catalysts located in zone II between two solid lines exhibit small differences in the free energy of the three intermediates *COOH, *CHO, and *CO, and are considered potential efficient catalyst candidates. It is worth mentioning that the width of region II, which is the width of the catalyst region that moderately breaks the linear scaling relationship, is determined by parameter *c* in the formula. In our screening process, this parameter *c* was set to 0.5 eV. The selection of this value directly affects the range of region II and also represents the tolerance of screening requirements for limiting potential. Further analysis of the adsorption structural transitions from *CO to *CHO (shown in Fig. 6 and Table S9†) revealed that categorizing catalysts based on geometrical structure alone is unreliable. For instance, only a subset of catalysts with an end-bridge to side-bridge transition for *CO to *CHO fit into the moderate deviation category (*e.g.*, ZnRu, ZnPt, CrNi, VRu, AlIr, AlRh, MnRh, MnIr), while others like CrPd, CrPt, FeRu, MnPd, NiIr, ZnIr, and ZnRh, although having the same transition, do not fit into region II. Although AlPd, AlPt, and VPd (side bridge II to side bridge II), ZnPt (end on right to side bridge II), CrRh (end on left to end bridge), RuRh, and NbAg (end on left to side on I) belong to other structural flows, they are also in region II where linear scaling relationships are moderately broken. Therefore, relying solely on the geometric structure flow direction as a basis for breaking the linear scaling relationship is not reliable. It is necessary to comprehensively consider the adsorption energy formed by the geometric structure and electronic structure to analyze the synergistic mechanism of DACs in the decoupling of intermediate adsorption energy.

**Fig. 7:**
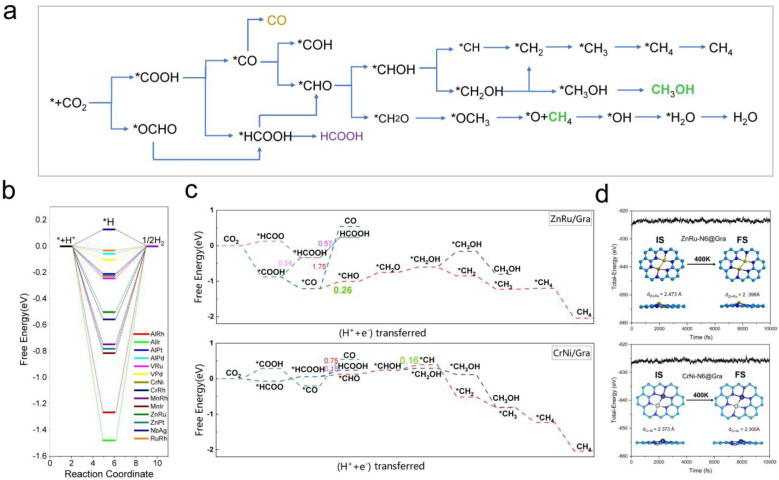
(a) Possible reaction pathways of the CO_2_RR on M1M2-N6@Gra catalysts. (b) Free energy graphs of HER side reactions on 14 M1M2-N6@Gra catalysts. (c) The free energy diagrams of ZnRu-N6@Gra and CrNi-N6@Gra in reducing CO_2_ to CH_4_ and CH_3_OH pathways highlight the rate limiting steps of methane production with green bold numbers. (d) Fluctuations of energy at 400 K for catalysts ZnRu-N6@Gra and CrNi-N6@Gra with atomic structure snapshots shown in insets.

Based on the analysis of the relationship between the adsorption energies of *COOH, *CO, and *CHO, we used the difference in adsorption energies between *CHO and *CO (*E*_b_[CHO] − *E*_b_[CO]) as descriptors to construct a volcano curve to study the Gibbs free energy changes of these three intermediates during the reaction process (Fig. 7). This method enables us to gain a deeper understanding of the energy relationship among the three key intermediates *COOH, *CO, and *CHO in the electrochemical reduction of CO_2_. According to the reversible hydrogen electrode (RHE), the equilibrium potential for the overall electrochemical reduction of CO_2_ to CH_4_ is +0.17 V, which theoretically limits the possibility of the catalyst operating at a potential more negative than the equilibrium potential. Therefore, the theoretical limiting potential for these two steps can be represented by the distance between the equilibrium line and the most negative limiting potential line. Setting the selection interval at −0.5 eV, we identified AlRh, AlIr, AlPd, AlPt, VRu, VPd, CrNi, CrRh, MnRh, MnIr, ZnRu, FeCu, CoPd, ZnPt, NbAg, and RuRh as catalysts capable of operating at lower limiting potential. However, FeCu and CoPd, having a significant Gibbs free energy difference in the step from *COOH to *CO, were excluded from further selection. Thus, only 14 catalysts remained as potential candidates for further selection.

From Fig. 5e, we can see that in the three proton electron pair steps of the first four intermediates, the diatomic catalysts with lower limiting potentials are VPd, AlPd, VRu, AlIr, ZnRu, NbAg, CrNi, AlRh, AlPt, RuRh, CrRh, ZnPt, MnRh, and MnIr, respectively. The detailed data are shown in Table S10.† However, AlRh, AlPd, VRu, and VPd exhibit high energy steps during the final electron protonation process of *H_2_O in path 1: CO_2_ → *COOH → *CO → *CHO → *CH_2_O → *CH_3_O → *O + CH_4_ → *O → *OH → *H_2_O, which hinders their CH_4_ production along this pathway (Fig. 5f and Table S11†). The main possible reaction pathways involved in the 14 candidate products CH_4_ in this study are shown in Fig. 7a, and the free energy of intermediates is shown in Tables S11–S13.† Next, we also considered searching for possible pathways to reduce the limiting potential through different intermediates. On path 2: CO_2_ → *COOH → *CO → *CHO → *CHOH → *CH → *CH_2_ → *CH_3_ → *CH_4_, the limiting potentials of VPd, CrNi, CrRh, and NbAg can be reduced to −0.47, −0.33, −0.45, and −0.43 V, respectively (Table S12†). On path 3: CO_2_ → *COOH → *CO → *CHO → *CH_2_O → *CH_2_OH → *CH_2_ → *CH_3_ → *CH_4_, the limiting potentials of AlRh, AlPd, and VRu can also be reduced, and the overpotential of ZnRu can be reduced to −0.26 eV (Table S13†). Based on the analysis of possible reaction pathways, the limiting potentials of AlPt, VPd, CrNi, CrRh, MnRh, MnIr, ZnRu, ZnPt, NbAg, and RuRh for CH_4_ production are −0.38, −0.47, −0.33, −0.45, −0.43, −0.45, −0.26, −0.52, −0.34, and −0.39 V, respectively. In addition, we also investigated them on the *CO_2_ → *HCOO → *HCOOH → *CHO pathway and found that the AlPt, CrNi, and CrRh in them can operate at low potentials of −0.30, −0.16, and −0.46 eV, respectively (Table S14†). As shown in Table S15,† we calculated the adsorption energy of the catalyst surface on the product. For the adsorption of CO and HCOOH, except for ZnPt and RuRh surfaces which are weakly adsorbed with HCOOH, all others maintain strong adsorption, indicating that the CO_2_RR of the vast majority of catalysts tends to continue towards deeper electron step reactions. These 14 catalysts are weakly adsorbed to CH_4_, which is very conducive to CH_4_ production. In general, the closer the free energy of the *H intermediate (ΔG_*H_) is to zero, the more HER is likely to occur. AlPt, AlPd, VRu, VPd, NbAg, and RuRh in Fig. 7b show ΔG_*H_ values closer to 0, so they are unfavorable for the CO_2_RR, and the remaining 8 catalysts can compete through hydrogen evolution. Based on the above analysis, ZnRu, ZnPt, CrNi, CrRh, MnRh and MnIr have limiting potentials of −0.26 V, −0.52 V, −0.16 V, −0.45 V, −0.43 V and −0.45 V, respectively. They can be used as diatomic catalysts with high stability, high reactivity and high selectivity for CH_4_ production. Among them, the optimal *U*_L_ values for ZnRu and CrNi were −0.26 V and −0.16 V, respectively, which exceeded most reports.^57^ Among them, the optimal *U*_L_ values for ZnRu and CrNi were −0.26 V and −0.16 V, respectively, which exceeded most reports. Among the 14 catalysts, VPd, CrNi, CrRh, MnIr, and RuRh showed weak adsorption to CH_3_OH and tended to produce CH_3_OH. After considering the hydrogen evolution competition and excluding VPd, NbAg and RuRh, CrNi, CrRh and MnIr produce CH_3_OH at a limiting potential of −0.33, −0.45 and −0.45 eV, respectively. The reaction paths and Gibbs free energies of the calculated intermediates are shown in Tables S16–S18.† Through *ab initio* molecular dynamics (AIMD) simulations, we further evaluated the stability of the best candidates ZnRu and CrNi. As shown in Fig. 7d, the catalyst structures hold up well, indicating that they can withstand the thermal conditions of the CO_2_RR. As is well known, the active sites on the catalyst surface are easily covered by various functional groups; if so, the reaction area on its base will be greatly reduced.^58,59^ In order to solve the problem of whether the catalyst surface is blocked by *O/*OH electron acceptors, we constructed the above 14 screening methods by plotting the relationship between equilibrium potentials and pH between different surface ends (including –O, –OH, and –H_2_O end systems) of M1M2-N6@Gra. The surface Pourbaix diagram of the catalyst is shown in Fig. S22.† Obviously, most catalysts except NbAg, VPd, AlRh, AlPd, and CrNi exhibit *U*_R_ values higher than *U*_L_ values, indicating that they are not affected by *O/*OH species under working conditions.

## Conclusions

In summary, our comprehensive study, facilitated by DFT calculations, has systematically explored the potential of graphene-based bimetallic catalysts in the electrocatalytic reduction of carbon dioxide (CO_2_RR). Through high-throughput screening of 465 M1M2-N6@Gra configurations, comprising 30 different metal atoms, we have successfully identified 14 catalysts exhibiting high activity, primarily in the efficient production of methane and methanol. Among these catalysts, ZnRu and CrNi show highly selective production of CH_4_ at an extremely low limiting potential of −0.26 V and −0.16 V, respectively, which exceeds the performance of most electrocatalysts reported so far under acid conditions. However, CrNi may be covered by *O/*OH under working conditions, which can affect its CO_2_RR performance. Both CrRh and MnIr can reduce CO_2_ to CH_3_OH at a limiting potential of −0.45 V. In addition, our research has further transcended traditional understandings of the inherent scaling relations between intermediate adsorption strengths in bimetallic catalysts for the CO_2_RR. Through the analysis of extensive high-throughput computational data, we established novel activity descriptors, introducing the concepts of linear scaling regions, moderate deviation zones, and severe deviation areas in the design of bimetallic catalysts. This breakthrough is of significant importance in guiding the understanding and design of more effective catalysts. In our analysis of catalyst stability, beyond conventional thermodynamic, electrochemical, and molecular dynamics approaches, we employed a machine learning (ML) model based on the random forest algorithm to identify key determinants of catalyst stability. Our findings highlight that electronegativity, atomic numbers (*Z*_1_, *Z*_2_), and d-electron counts are crucial properties determining the stability of the catalysts, with electronegativity playing a pivotal role. Our study has unveiled a unique mechanism of electron orbital interactions in the adsorption and activation processes of carbon dioxide on bimetallic catalysts. We discovered that the electron orbitals of the two metal atoms establish independent “donor–acceptor” dual-channel interactions with the π_g_ and 
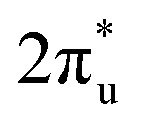
 orbitals of CO_2_ molecules near the Fermi level. The bidirectional electron interactions occurring within these independently constructed dual channels of bimetallic atoms not only facilitate effective adsorption and activation of CO_2_ molecules but also demonstrate markedly enhanced modulatory flexibility and efficiency compared to monometallic systems. This discovery opens new avenues for the design of innovative catalysts, providing fresh insights and directions in the field of catalyst development.

## Methods

All calculations in this study were conducted using spin-polarized density functional theory (DFT) within the Vienna *Ab initio* Simulation Package (VASP).^60,61^ The exchange–correlation energy was addressed using the Perdew–Burke–Ernzerhof (PBE)^62^ functional within the Generalized Gradient Approximation (GGA). The ion–electron interaction was described by the Projector Augmented-Wave (PAW)^63^ method. A kinetic energy cutoff for the plane-wave basis set was set at 450 eV. The Brillouin zone was sampled using a 3 × 3 × 1 Monkhorst–Pack *k*-point grid for structural optimization and electronic property analyses, such as Bader charge transfer and charge density differences. To accurately analyze the electronic density of states (DOS), a denser *k*-point mesh of 9 × 9 × 1 was utilized. Additionally, a vacuum space of 20 Å was applied perpendicular to the two-dimensional layers to avoid interactions between periodic images. The convergence thresholds for energy and force in self-consistent field (SCF) iterations were set to 10^−5^ eV and 0.02 eV Å^−1^, respectively. van der Waals interactions (vdWs) between the adsorbate and interface were considered using the empirical density functional dispersion correction (DFT-D3).^64^

The free energy calculations for intermediate states from reactants to products were based on the Computational Hydrogen Electrode (CHE) model.^65^ The change in free energy (Δ*G*) was calculated using the following formula:^66^

3 Δ*G* = Δ*E* + Δ*E*_ZPE_ − *T*Δ*S*

Here, Δ*E* represents the energy change based on the DFT method, while Δ*E*_ZPE_ and Δ*S* denote the zero-point energy correction and the entropy change at room temperature (*T* = 298.15 K), respectively. Post-processing of the computational results was aided by the VASPKIT software.^67^

The limiting potential (*U*_L_) was defined as:

4 *U*_L_ = −Δ*G*_max_/*e*

where Δ*G*_max_ is the free energy change at the limiting potential step in the CO_2_RR process, representing the minimum potential required to drive the entire reduction reaction.

## Author contributions

Xin Jia proposed the research concept and supervised the progress of the entire project. Yanwen Zhang and Zhaoqun Yao are responsible for designing research plans and research methods. Feng Zhang and Zhirong Guo conducted mathematical modeling of the catalyst structure. Zhaoqun Yao conducted the main calculations and data collection. YiMing Yang and Yunxia Liang plotted the data. Xingwu Zhai and Yanwen Zhang analyzed and explained the data. Yanwen Zhang and Guixian Ge have written the initial draft of the paper and made multiple revisions. All authors have reviewed and edited the final draft and agreed to publish it.

## Conflicts of interest

The authors declare no competing financial interests.

## Supplementary Material

SC-015-D4SC03085K-s001

## Data Availability

The authors confirm that the data supporting the findings of this study are available within the article.
