# Supplementary material for: Breaking the scaling relations of effective CO2 electrochemical reduction in diatomic catalysts by adjusting the flow direction of intermediate structures
Source: Chem Sci. 2024 Aug 8;15(33):13160–72. doi: 10.1039/d4sc03085k (PMC11310890; doi:10.1039/d4sc03085k)
Supplement: SC-015-D4SC03085K-s001 [file SC-015-D4SC03085K-s001.pdf]

Supplementary Information (SI) for Chemical Science.
This journal is © The Royal Society of Chemistry 2024

Supporting Information

Breaking the scaling relations of effective CO_2 electrochemical reduction in diatomic catalysts by adjusting the flow direction of intermediate structures

Yanwen Zhang, †[a,b] Zhaoqun Yao, †[c] YiMing Yang, [b] Xingwu Zhai,[d] Feng Zhang, [e] Zhirong Guo, [b] Xinghuan Liu, [a] Bin Yang, [a] Yunxia Liang, [b] Guixian Ge *[b] and Xin Jia *[a]

School of Chemistry and Chemical Engineering/State Key Laboratory Incubation Base for Green Processing of Chemical Engineering, Shihezi University, Shihezi 832003, China,
Email:jiaxin@shzu.edu.cn

Details of adsorption energy calculation:

Based on the theory of Nørskov et al., we calculate the binding energy of the metal active center of diatomic catalyst adsorbing intermediates *CO, *COOH and *HCOO with the following equation:[1]

$E_b(CO) = E(*CO) + \Delta E_{sol}(CO) – E* – E(CO(g))$

$E_b(COOH) = E(*COOH) + \Delta E_{sol}(COOH) – E* - E(CO_2(g)) – 1/2E(H_2)$

$E_b(CHO) = E(*CHO) + \Delta E_{sol}(CHO) – E* – E(CO(g)) – 1/2E(H_2)$

$E_b(CO)$, $E_b(COOH)$, and $E_b(CHO)$ are the binding energies of *CO, *COOH, and *CHO,respectively. The ΔE_{sol} indicates a solvation correction to binding energy. For *CO, *COOH,and *CHO, the solvation correction is -0.1 eV, -0.25 eV and -0.1 eV, respectively.

where ΔE_{sol} represents the solvation correction. Here we considered constant solvation correction, which was assigned as -0.1 eV and -0.25 eV for *CO and *COOH, respectively.

References

(1) K. Chan, C. Tsai, H. A. Hansen and J. K. Nørskov, ChemCatChem, 2014, 6, 1899-1905.

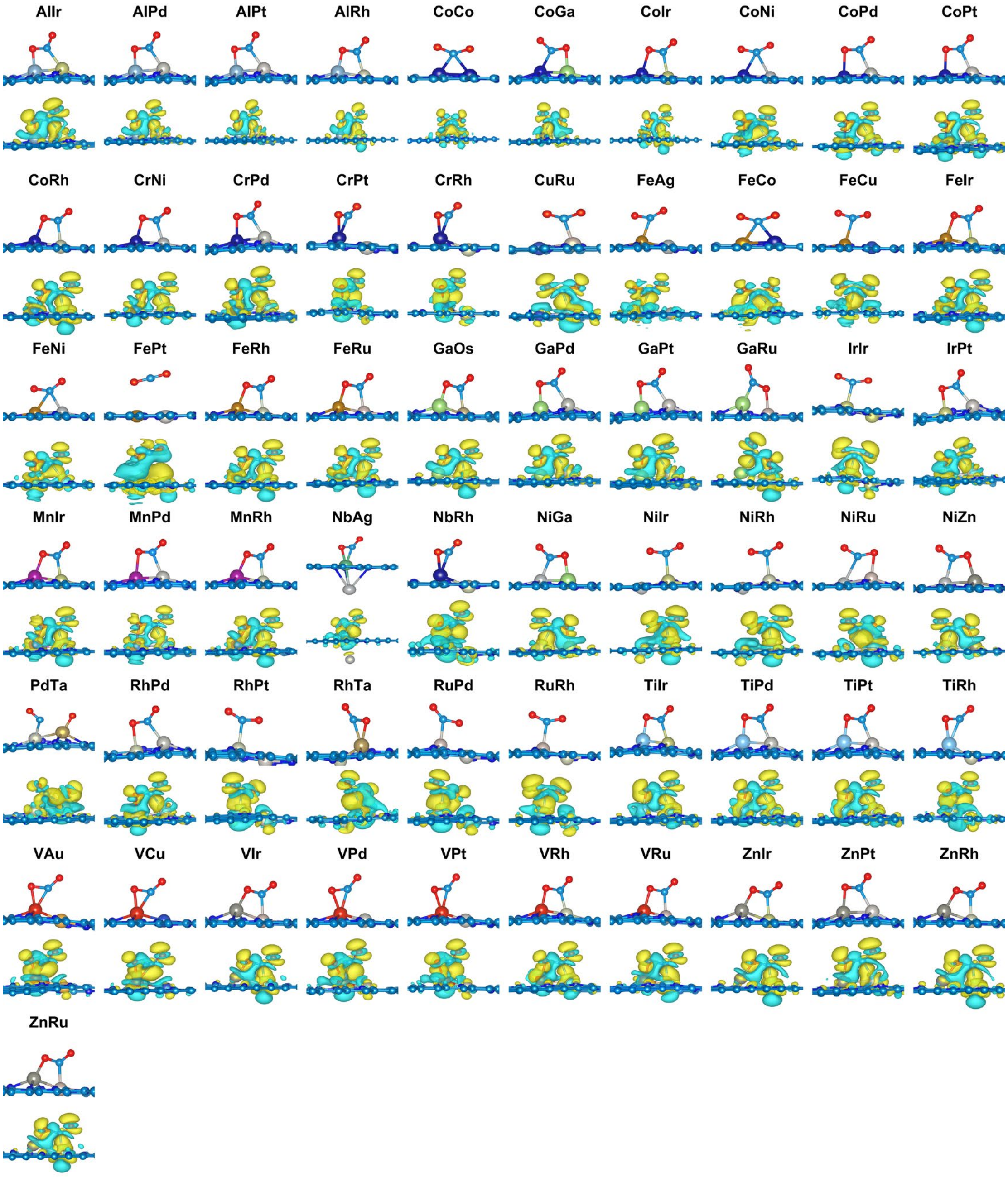

Figure S1. Optimized adsorption configurations and charge density differences for chemisorbed CO_2 on 61 different M1M2-N6@Gra surfaces.

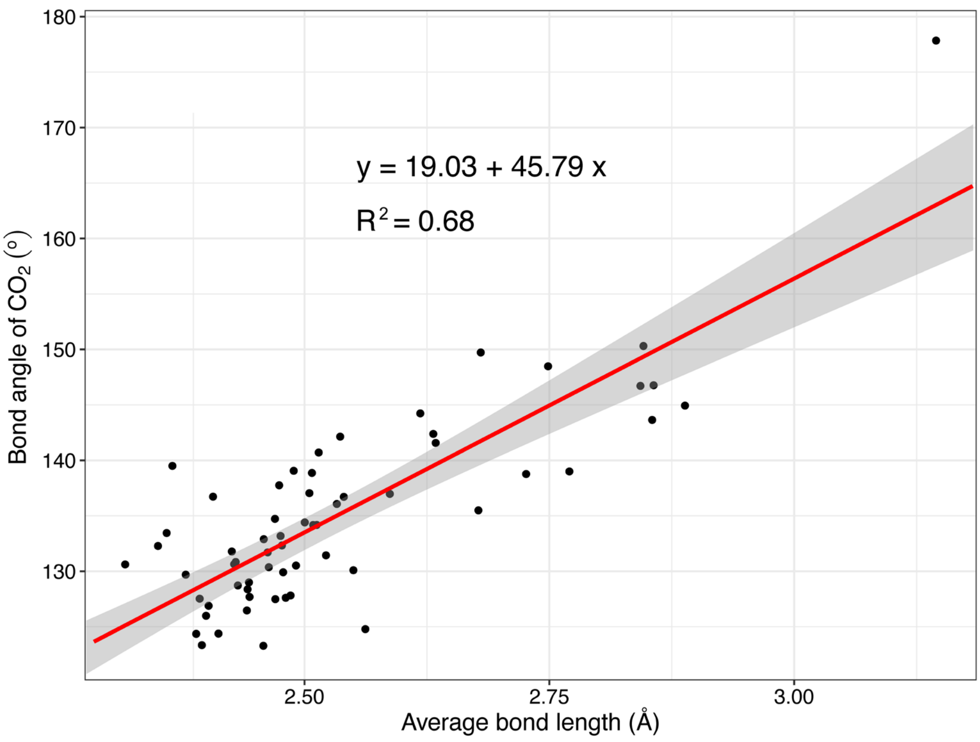

Figure S2. Linear relationship and Pearson coefficients between activation angles and average bond lengths.

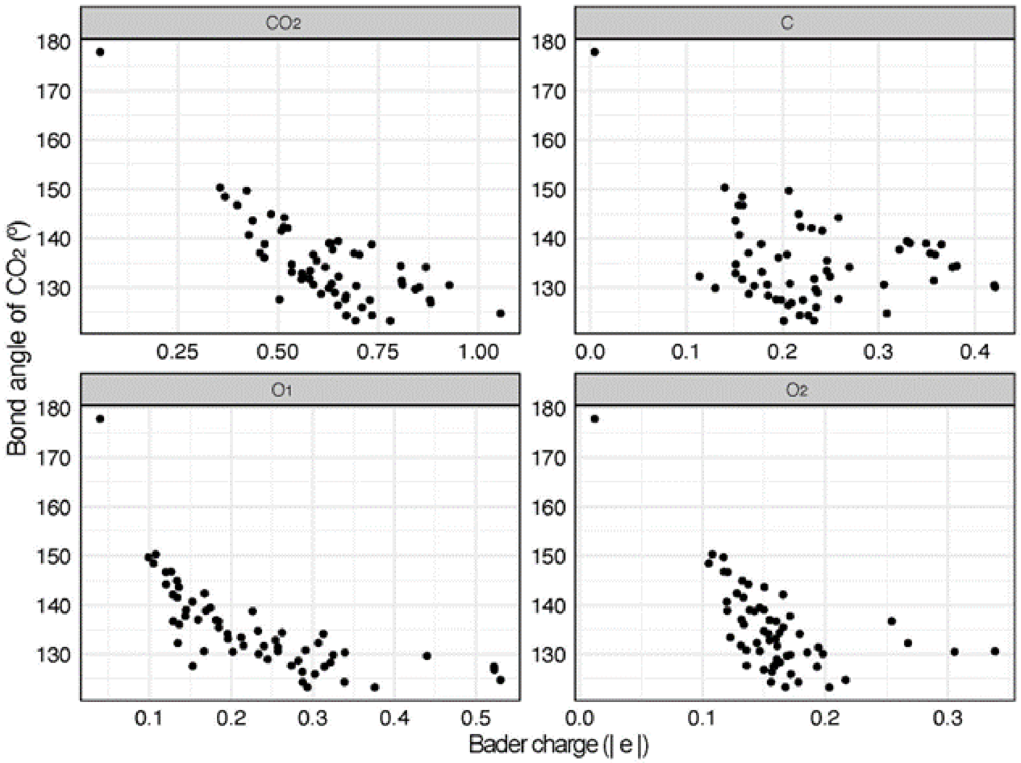

Figure S3. (a) Activation angles of CO_2 molecules chemisorbed on M1M2-N6@Gra surfaces correlated with (a) Bader charge of CO_2, (b) Bader charge of carbon atoms in CO_2, (c) Bader charge of O1, and (d) Bader charge of O2.

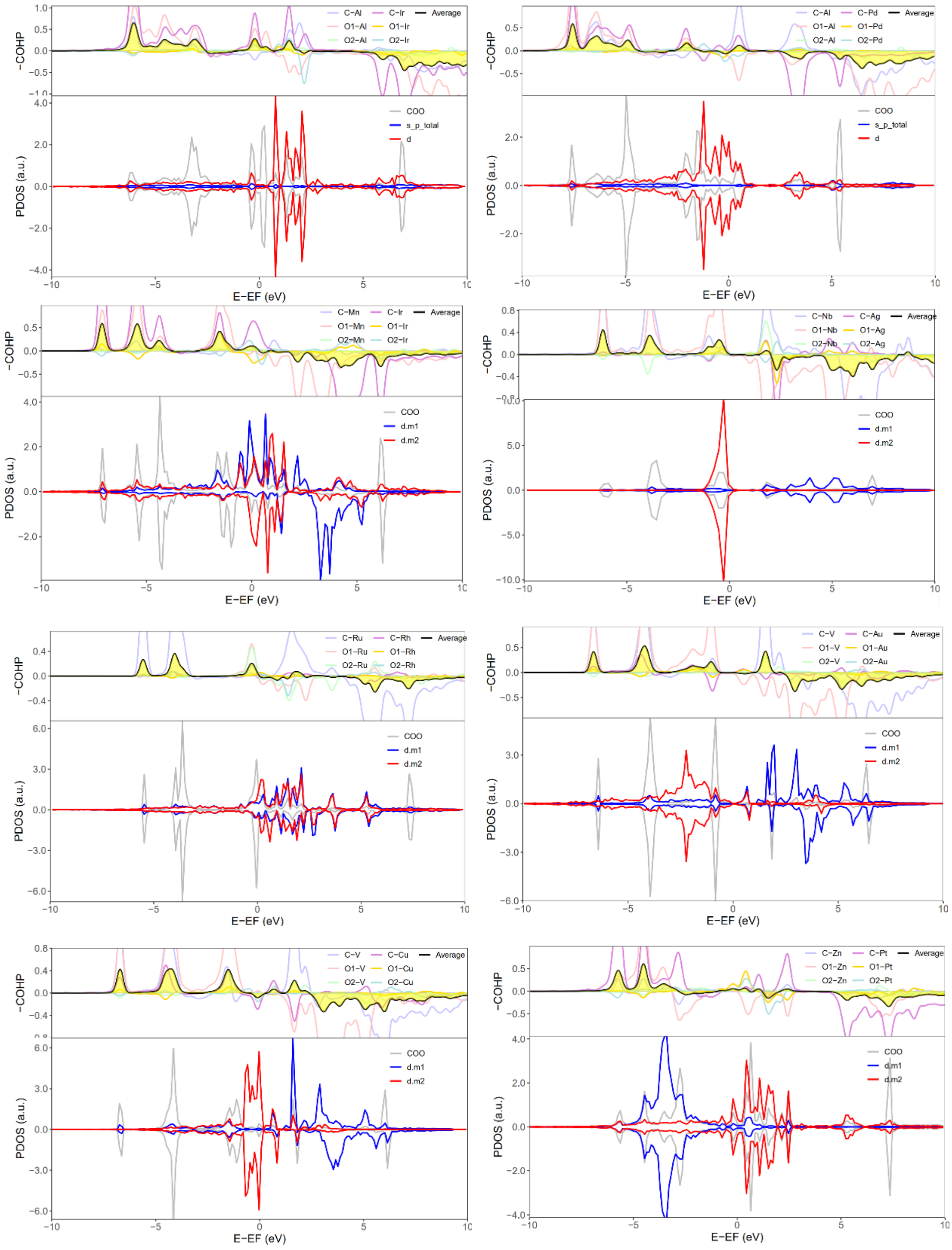

Figure S4. PDOS and COHP for CO_2 adsorption on M1M2-N6@Gra (M1M2=AlIr, APd, MnIr, NbAg, RuRu, VAu, VCu, ZnPt) surfaces. The Fermi level is set at 0 eV. Positive COHP indicates bonding states and negative indicates antibonding states; colored lines are COHP between two metal atoms and C, O1, and O2 atoms, with black representing average COHP.

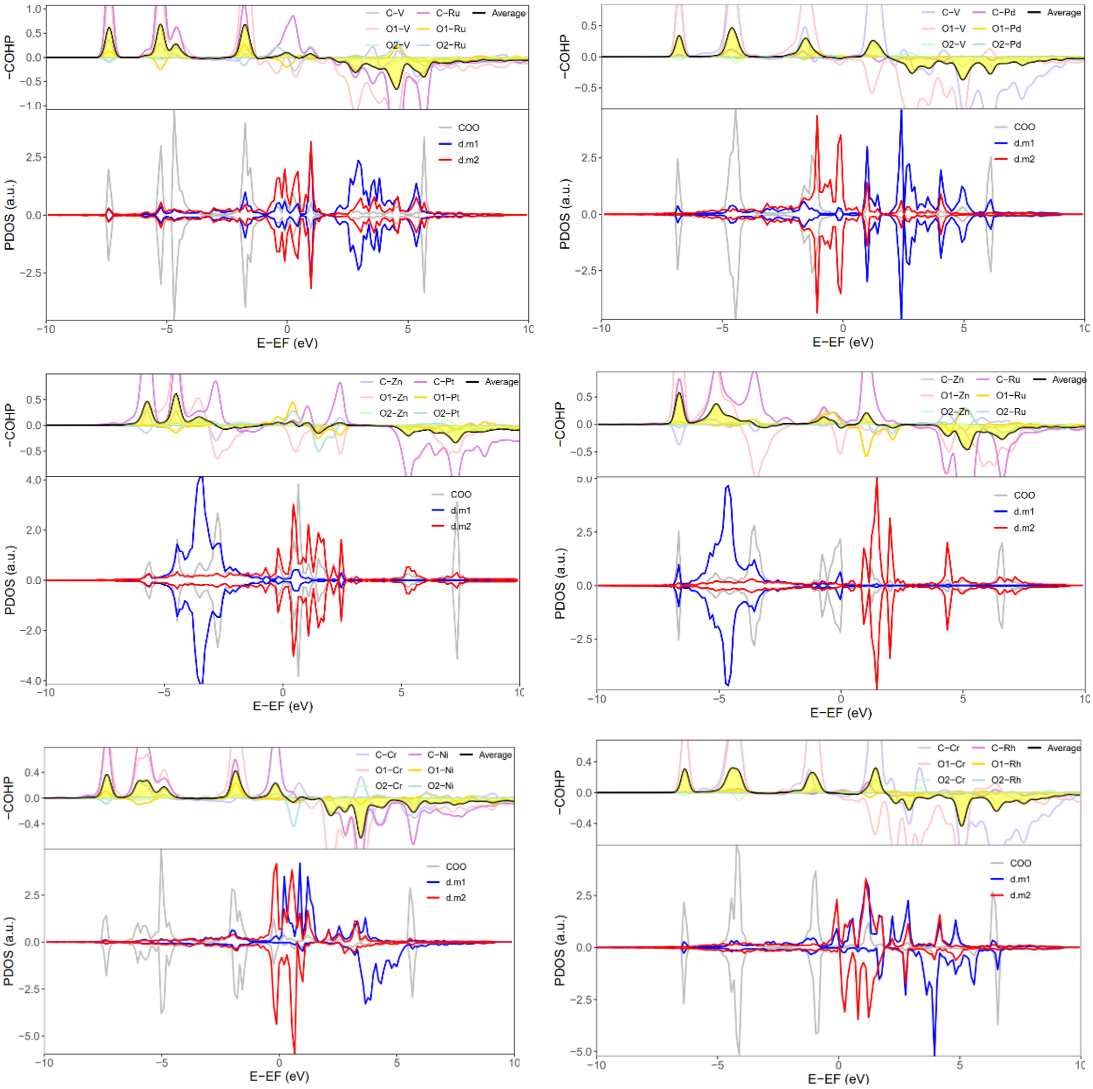

Figure S5. PDOS and COHP for CO_2 adsorption on M1M2-N6@Gra (M1M2=VRu, VPd, ZnPt, ZnRu, CrNi, CrRh) surfaces. The Fermi level is set at 0 eV. Positive COHP indicates bonding states and negative indicates antibonding states; colored lines are COHP between two metal atoms and C, O1, and O2 atoms, with black representing average COHP.

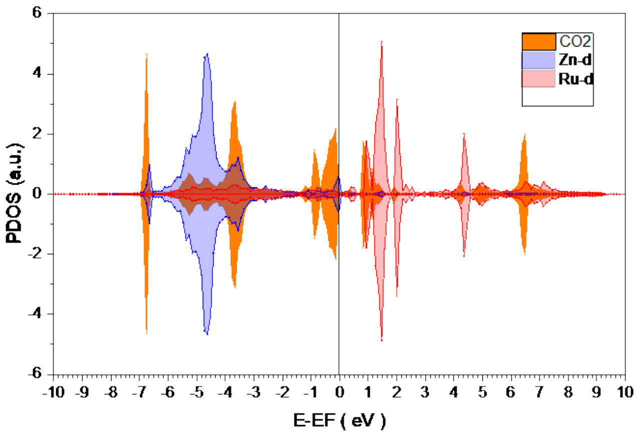

Figure S6. PDOS for CO_2, Zn, and Ru atoms after CO_2 adsorption on ZnRu-N6@Gra.

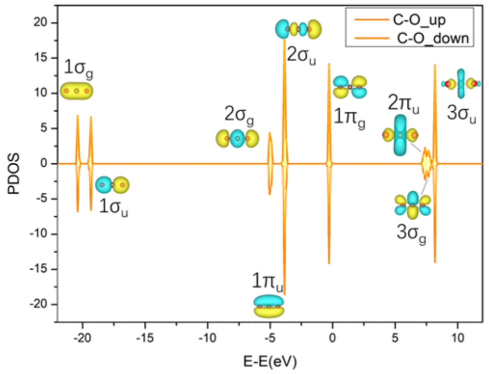

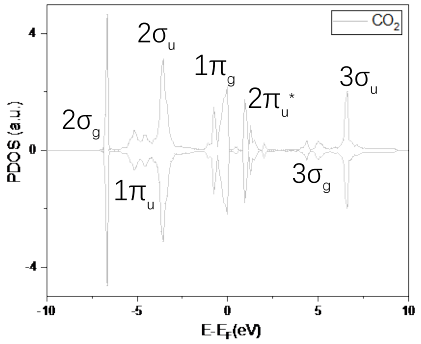

Figure S7. (a) PDOS of a free CO_2 molecule. (b) PDOS of CO_2 after adsorption. The Fermi level is set at 0 eV.

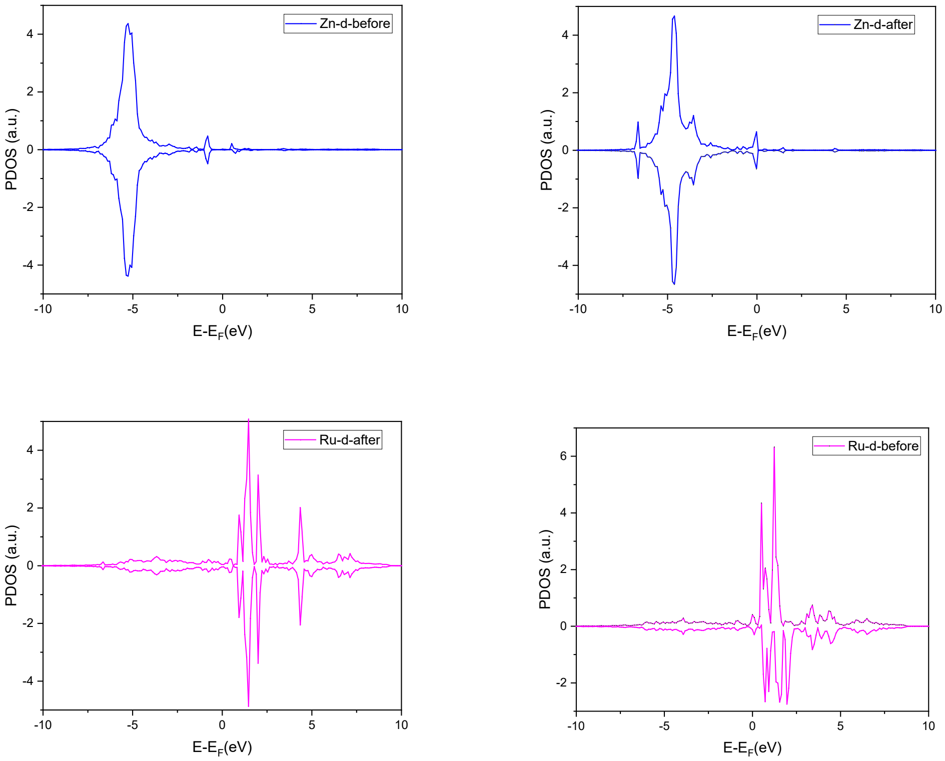

Figure S8. Calculated partial density of states (PDOS) for d electrons on ZnRu-N6@Gra surfaces before and after CO_2 adsorption.

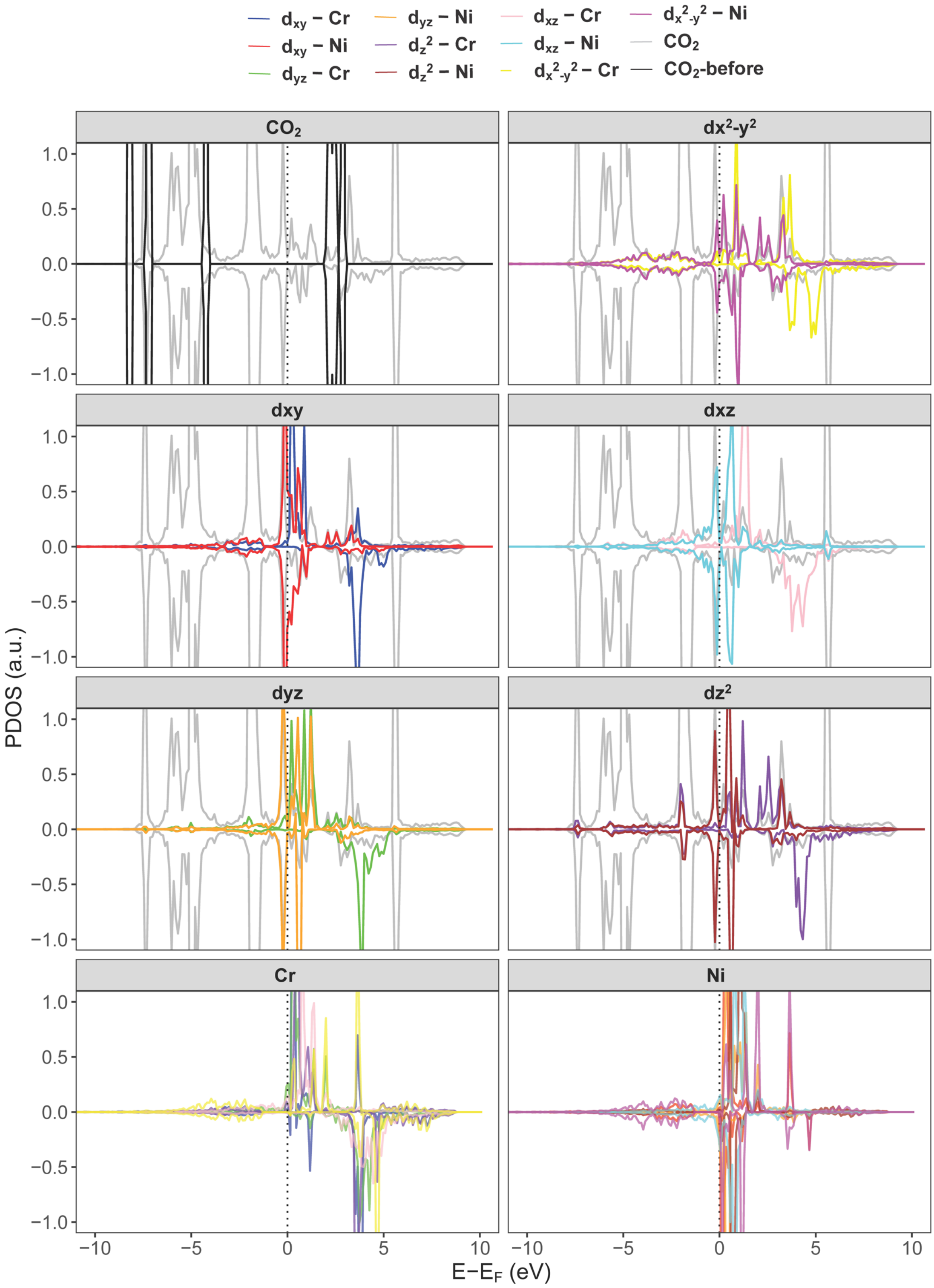

Figure S9. PDOS calculations for Cr and Ni d orbitals in CrNi-N6@Gra before and after CO_2 adsorption. The Fermi level is set at 0 eV.

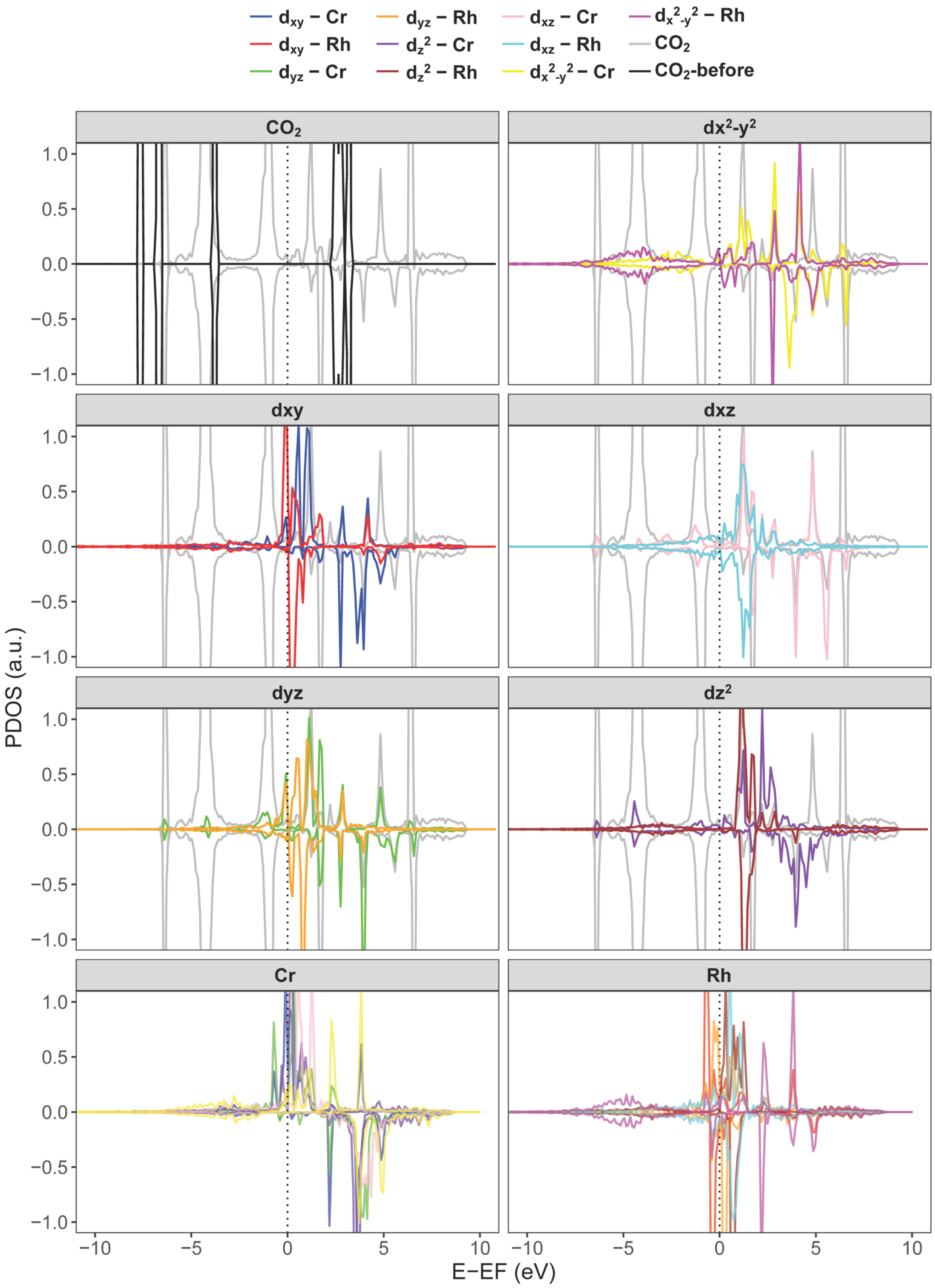

Figure S10. PDOS calculations for Cr and Rh d orbitals in CrRh-N6@Gra before and after CO_2 adsorption. The Fermi level is set at 0 eV.

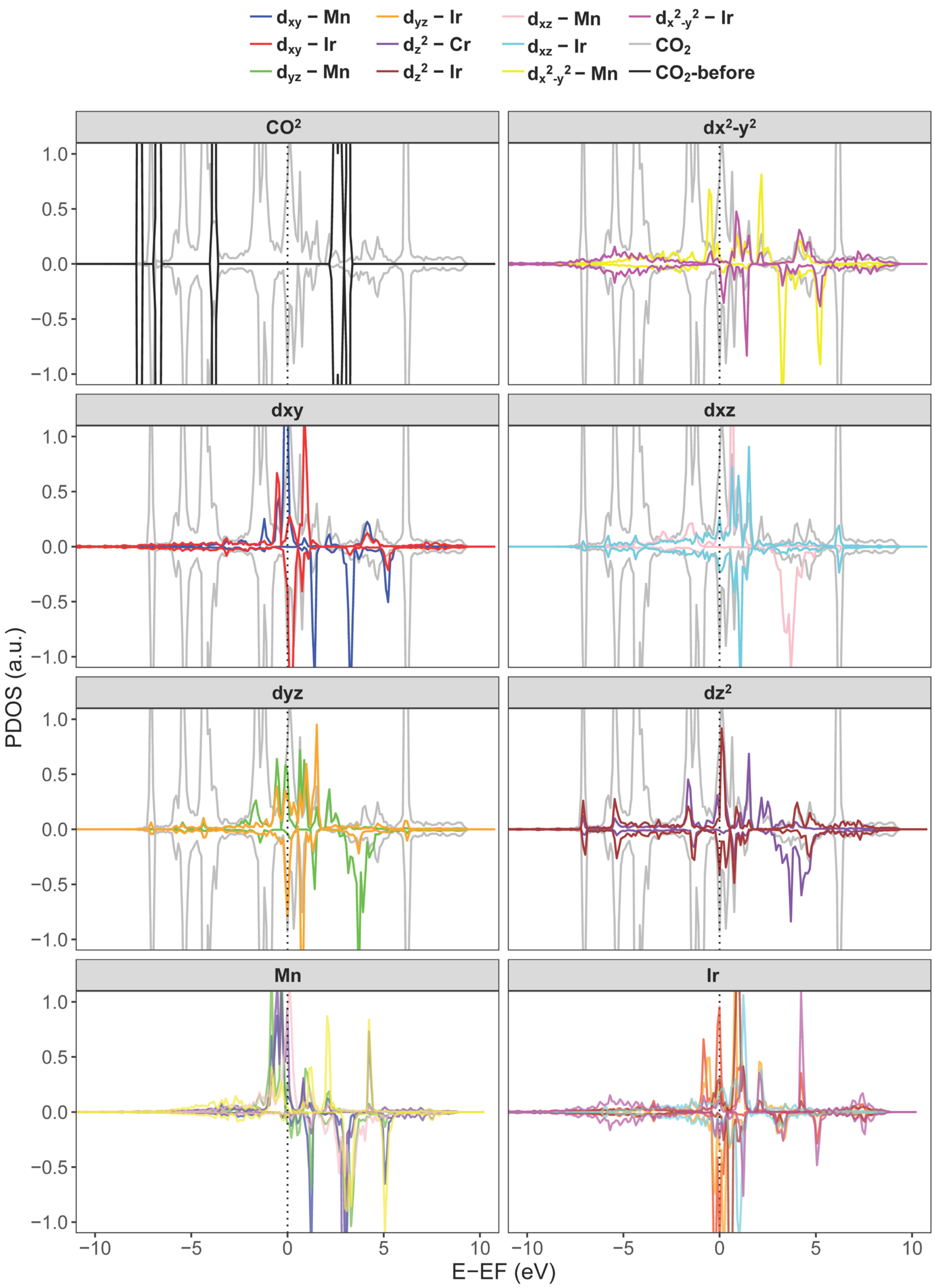

Figure S11. PDOS calculations for Mn and Ir d orbitals in MnIr-N6@Gra before and after CO_2 adsorption. The Fermi level is set at 0 eV.

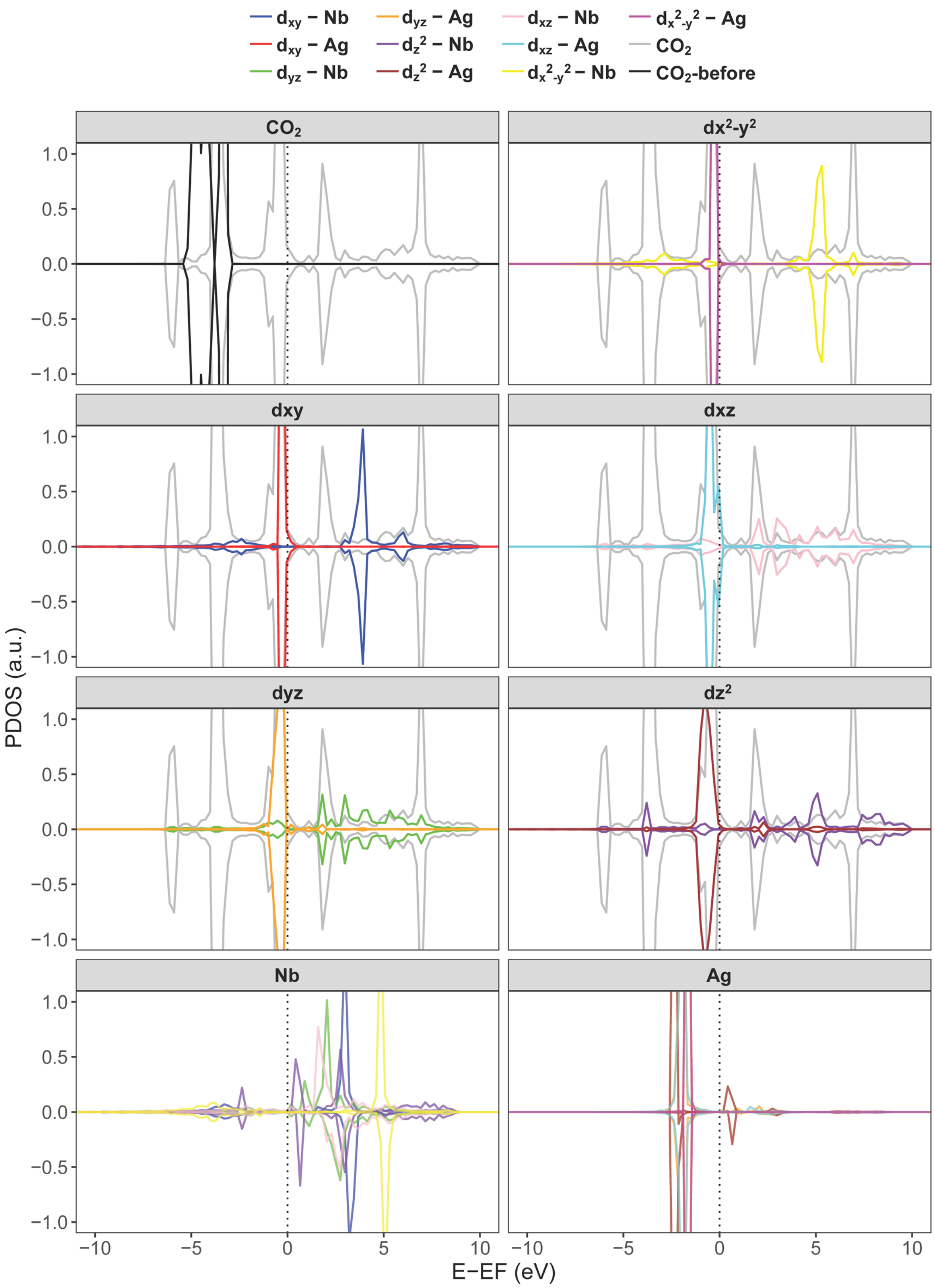

Figure S12. PDOS calculations for Nb and Ag d orbitals in NbAg-N6@Gra before and after CO_2 adsorption. The Fermi level is set at 0 eV

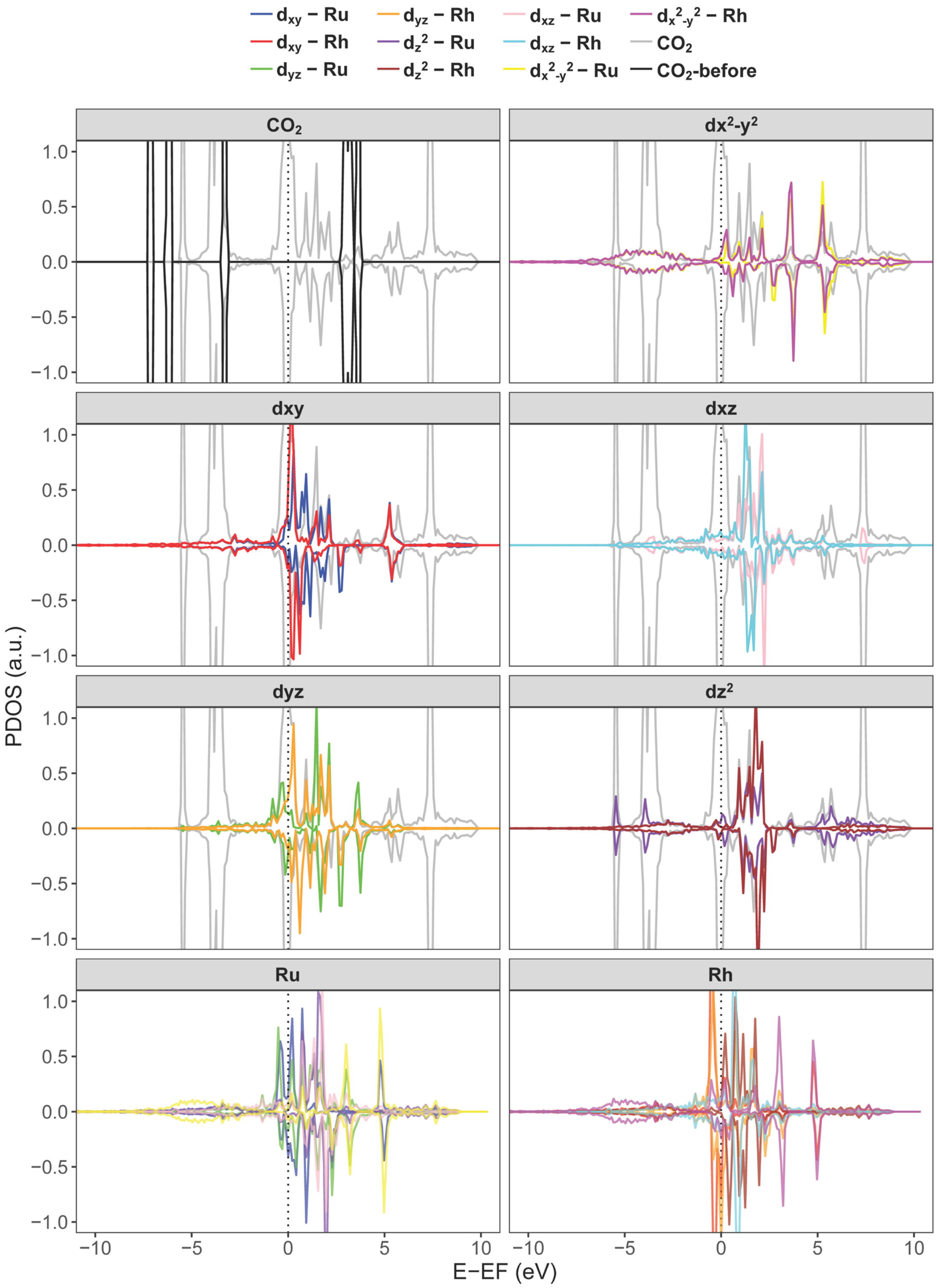

Figure S13. PDOS calculations for Ru and Rh d orbitals in RuRh-N6@Gra before and after CO_2 adsorption. The Fermi level is set at 0 eV.

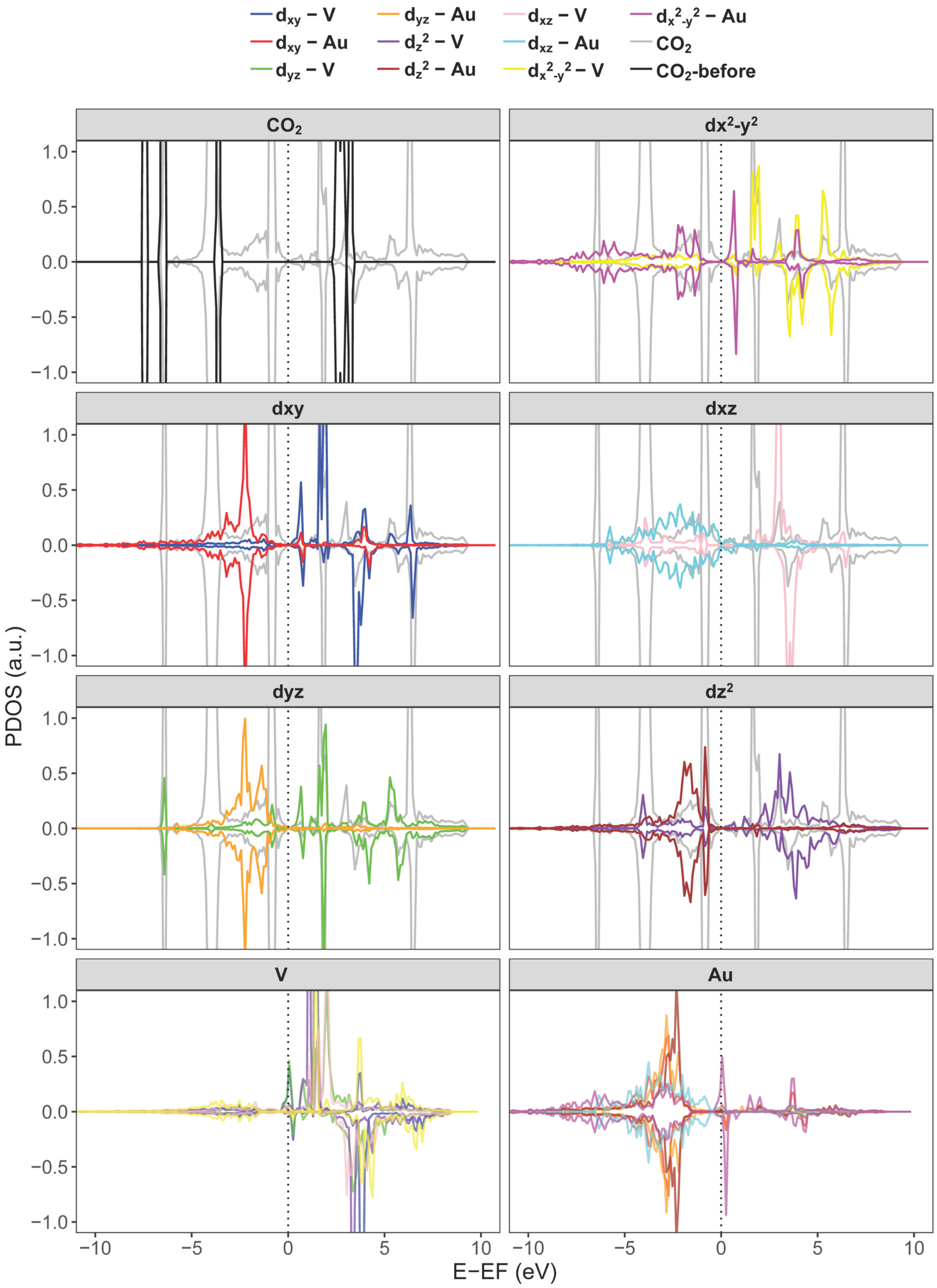

Figure S14. PDOS calculations for V and Au d orbitals in VAu-N6@Gra before and after CO_2 adsorption. The Fermi level is set at 0 eV.

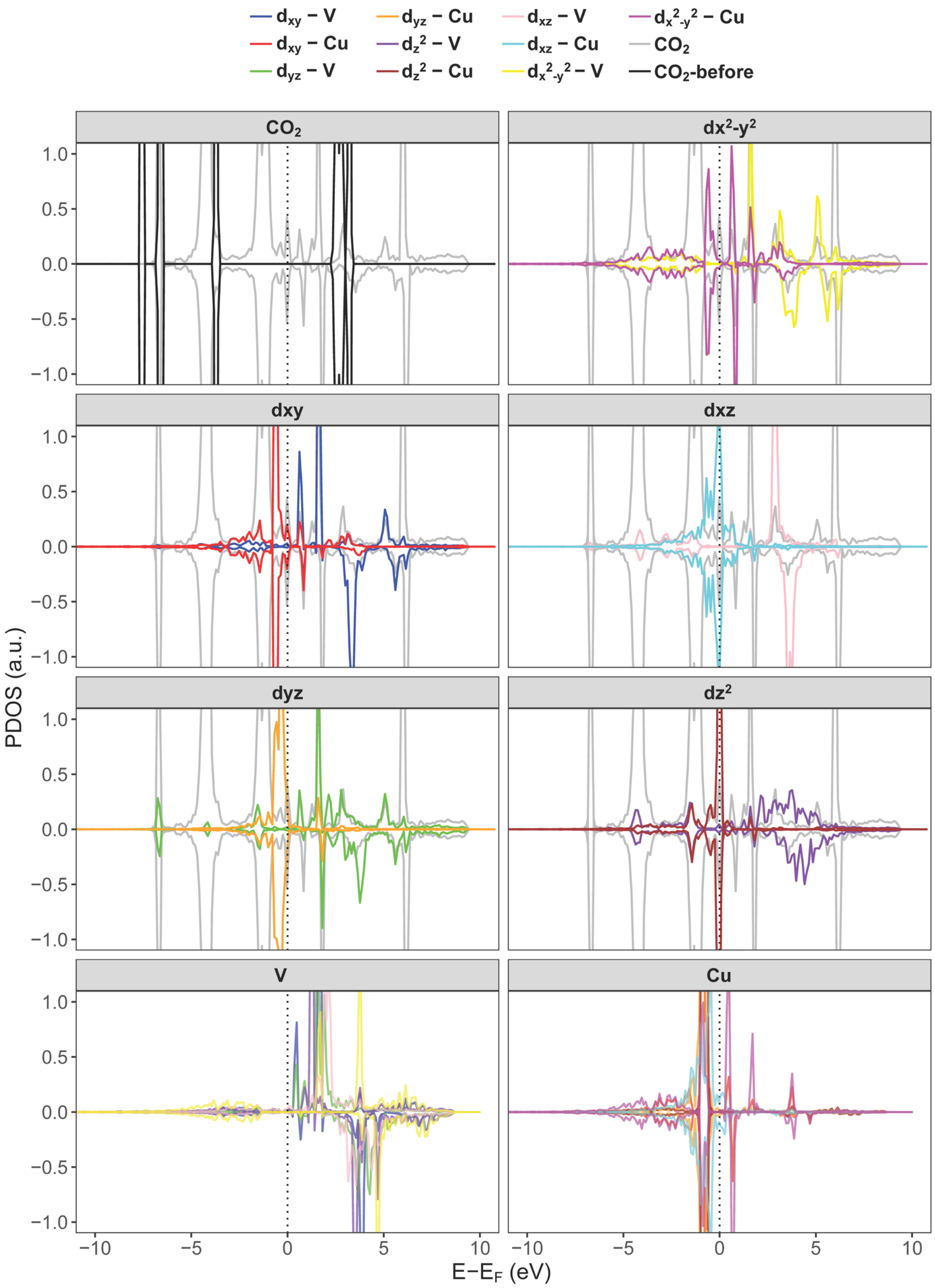

Figure S15. PDOS calculations for V and Cu d orbitals in VCu-N6@Gra before and after CO_2 adsorption. The Fermi level is set at 0 eV.

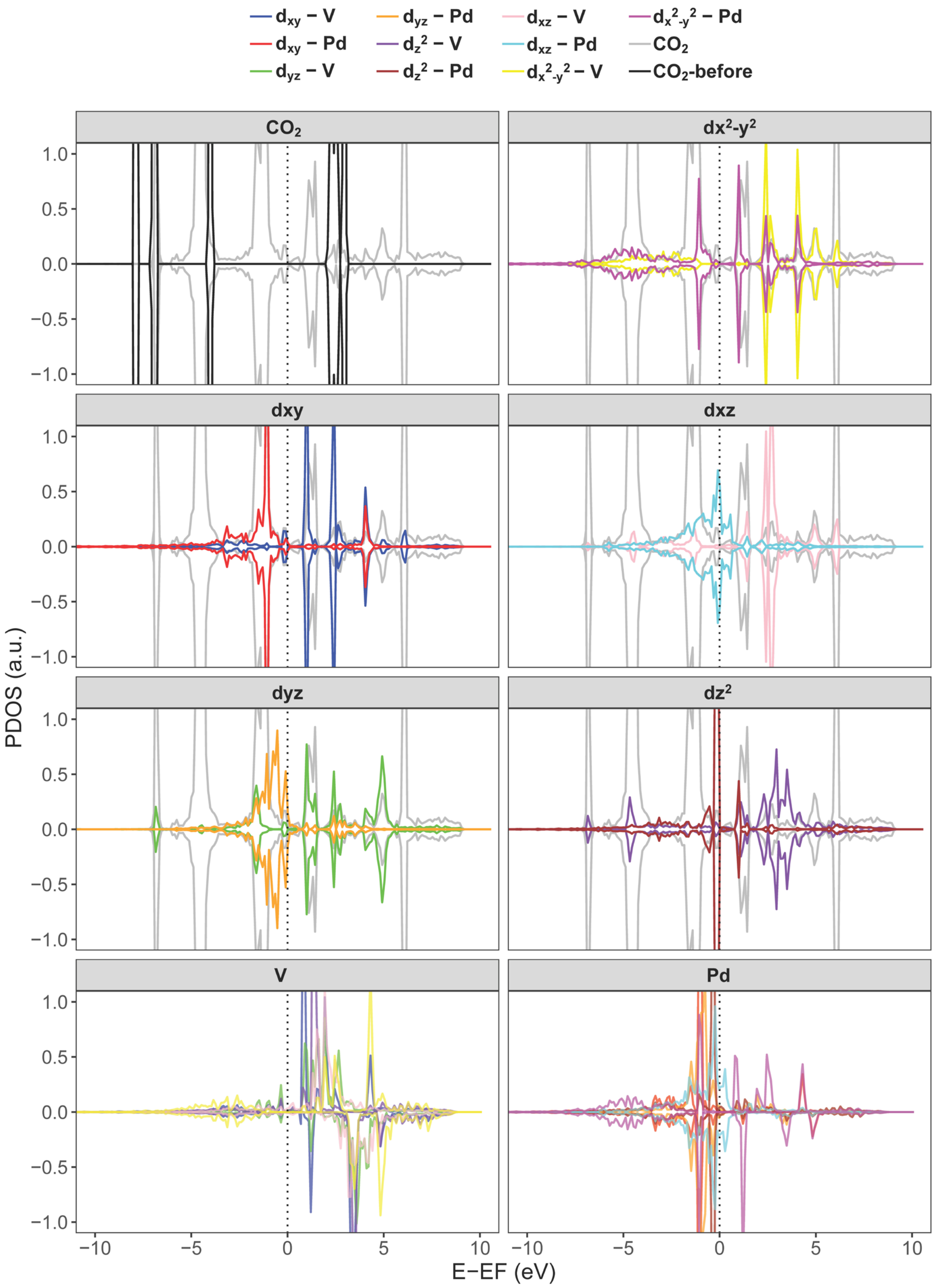

Figure S16. PDOS calculations for V and Pd d orbitals in VPd-N6@Gra before and after CO_2 adsorption. The Fermi level is set at 0 eV.

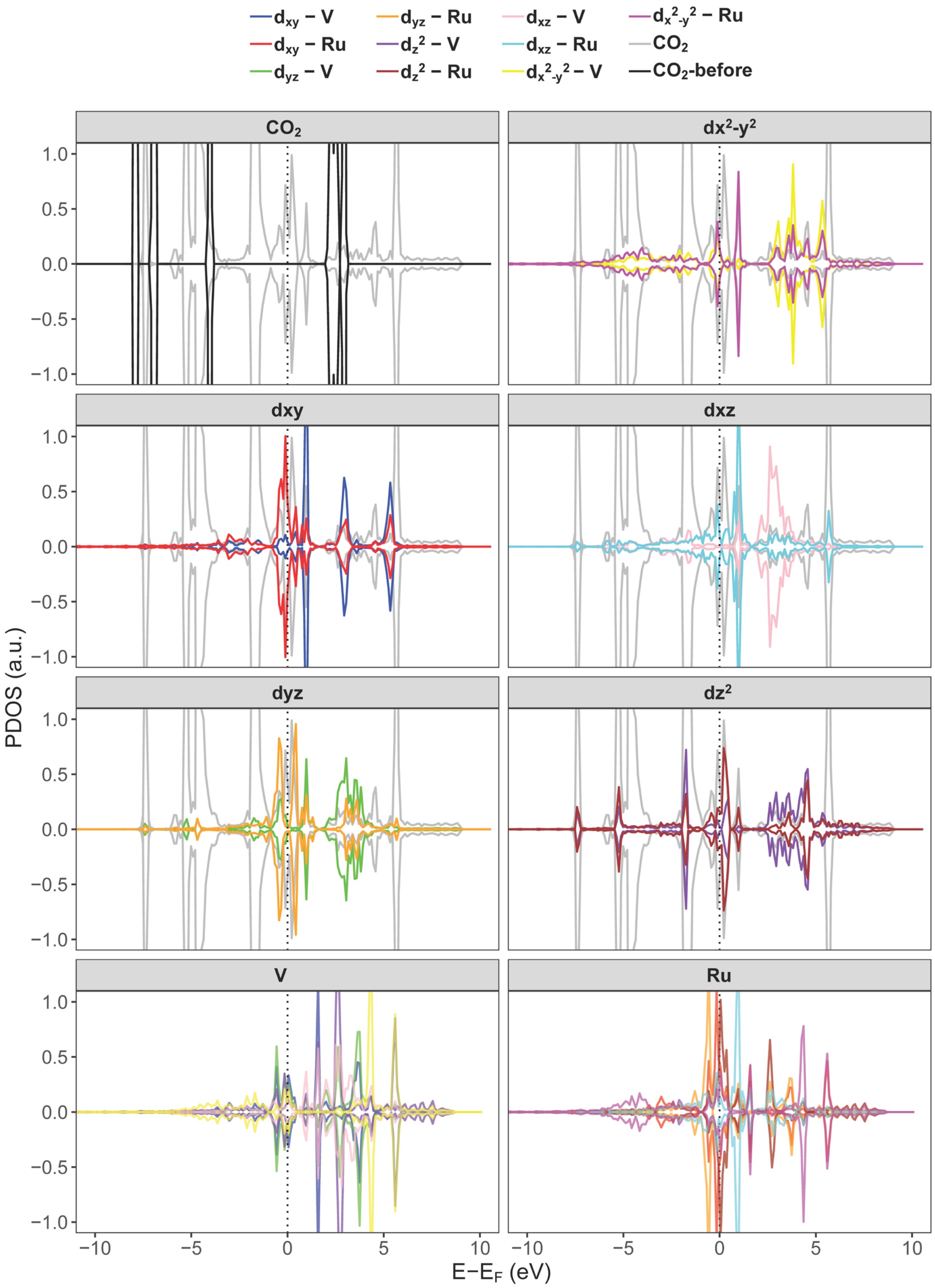

Figure S17. PDOS calculations for V and Ru d orbitals in VRu-N6@Gra before and after CO_2 adsorption. The Fermi level is set at 0 eV.

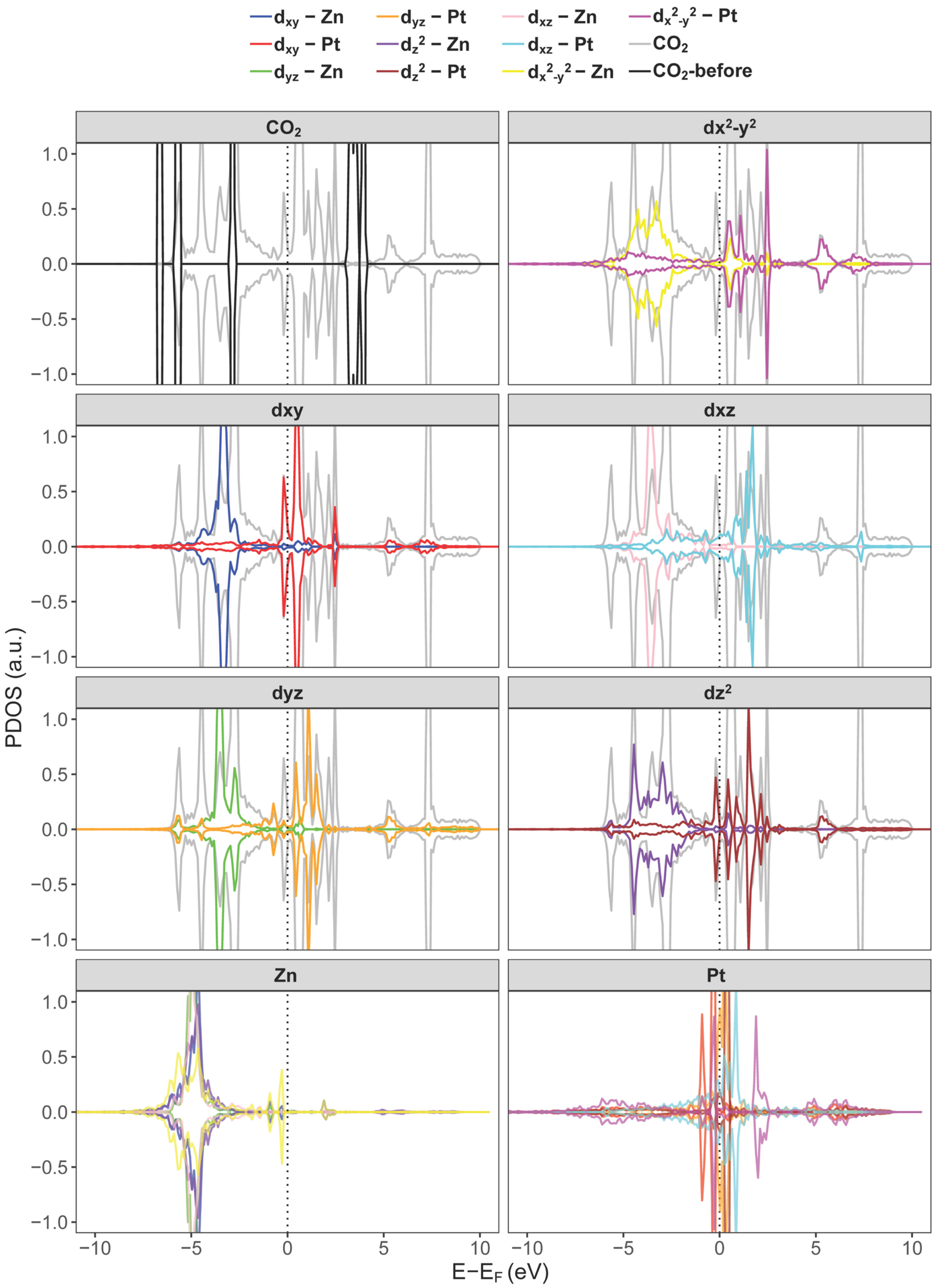

Figure S18. PDOS calculations for Zn and Pt d orbitals in ZnPt-N6@Gra before and after CO_2 adsorption. The Fermi level is set at 0 eV.

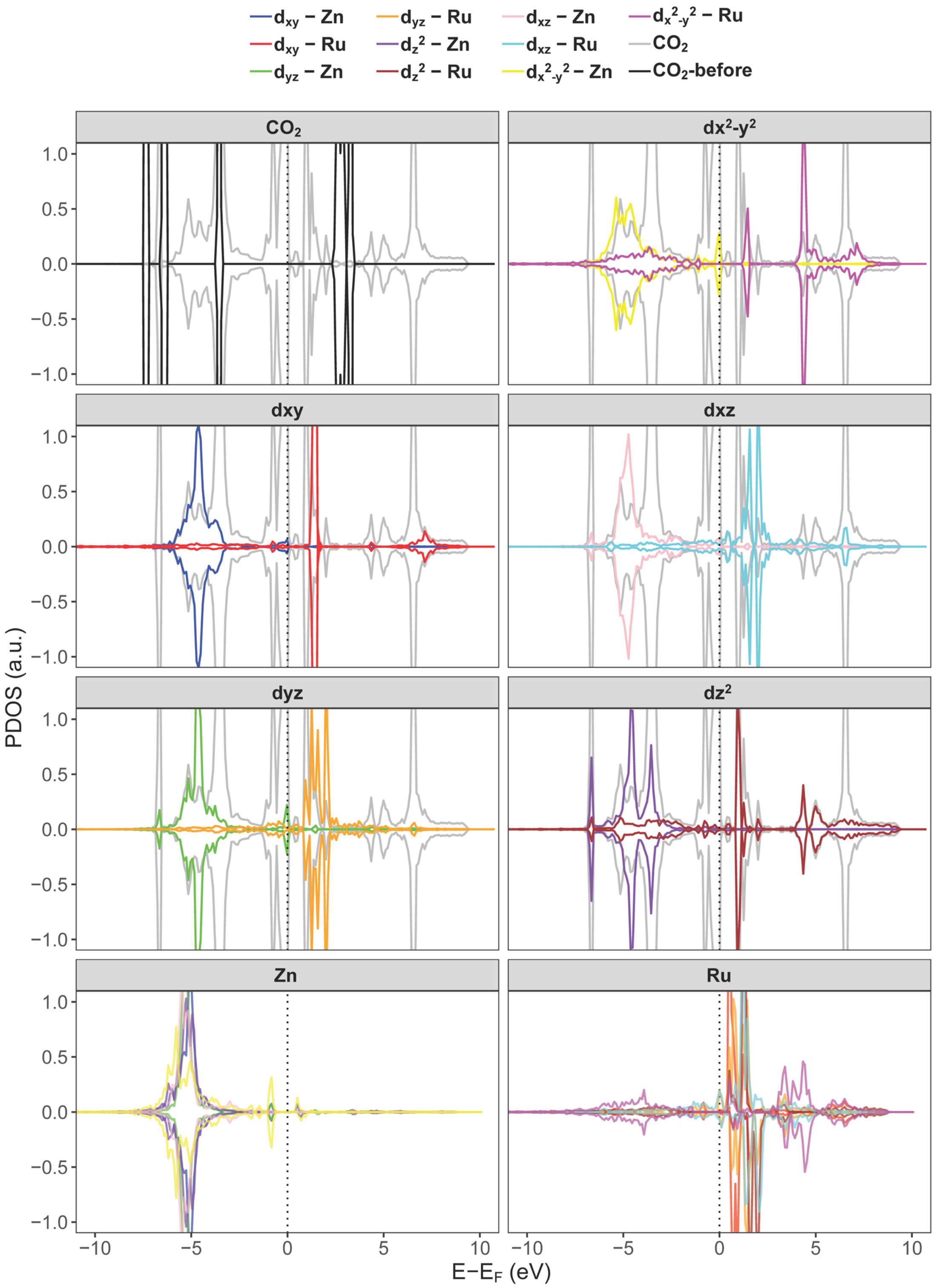

Figure S19. PDOS calculations for Zn and Ru d orbitals in ZnRu-N6@Gra before and after CO_2 adsorption. The Fermi level is set at 0 eV.

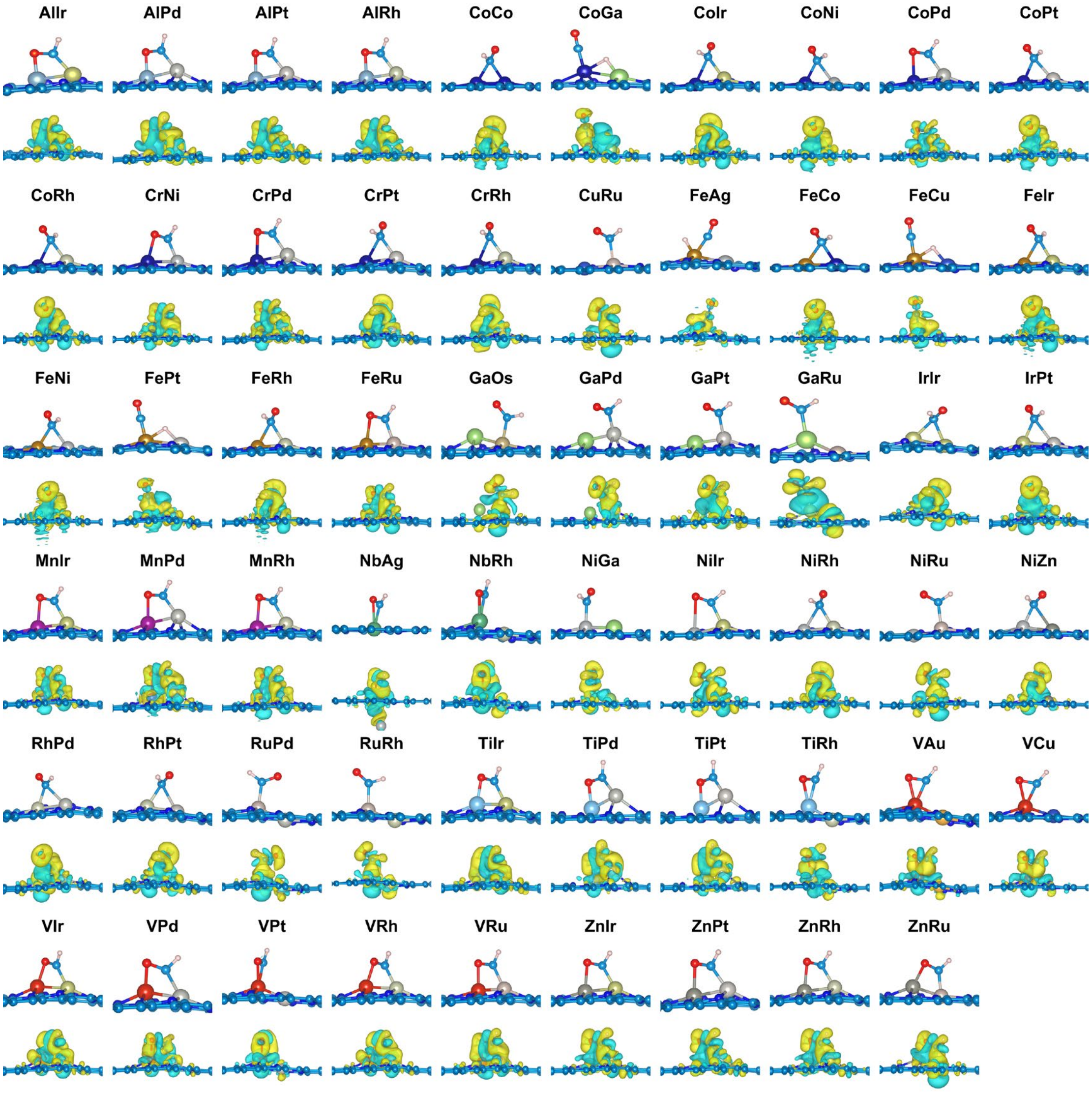

Figure S20. Optimized adsorption configurations and charge density differences for chemisorbed *CHO on 59 different M1M2-N6@Gra.

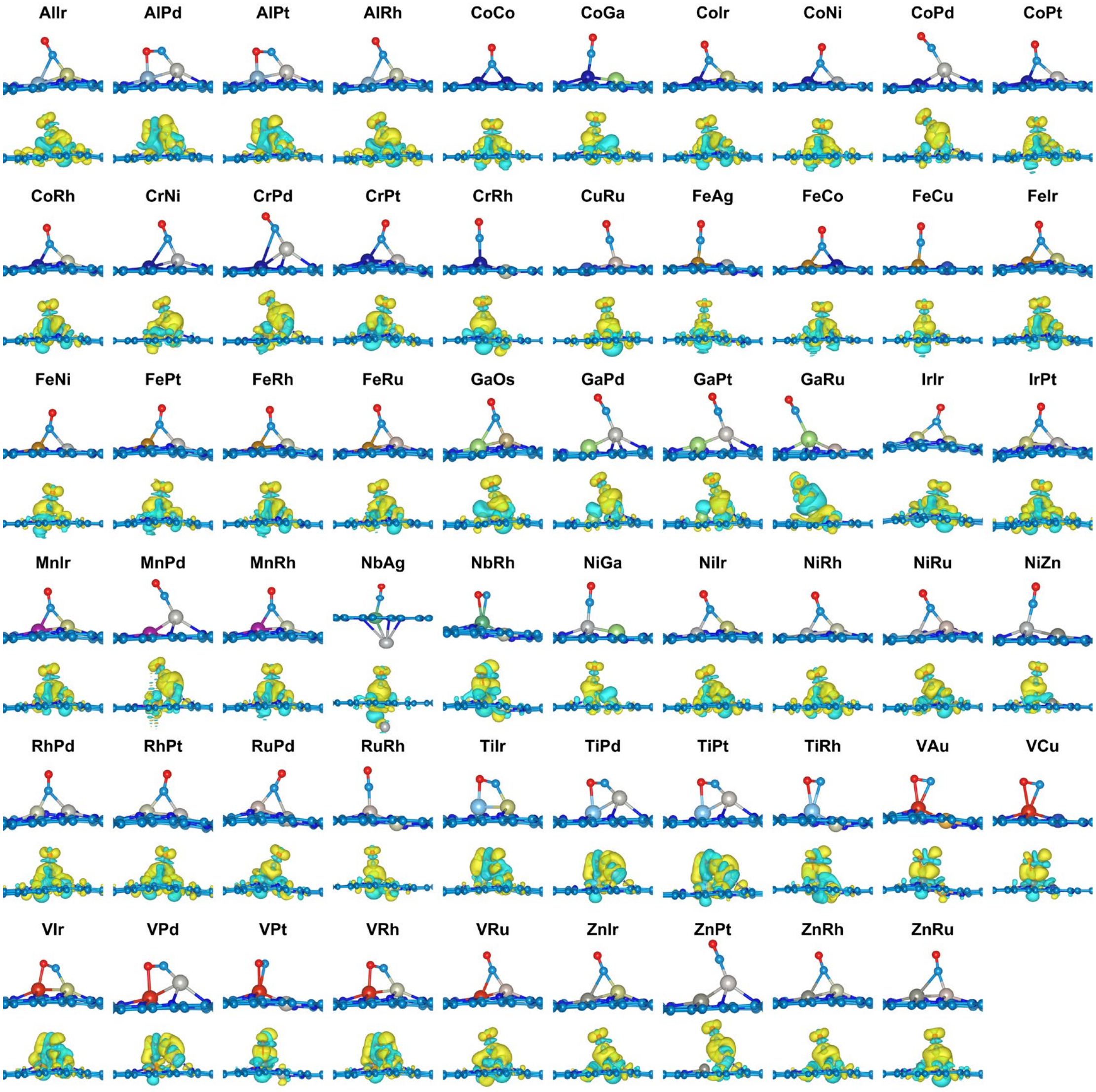

Figure S21. Optimal adsorption configurations and charge density differences of 59 M1M2-N6@Gra surface chemisorbed *CO.

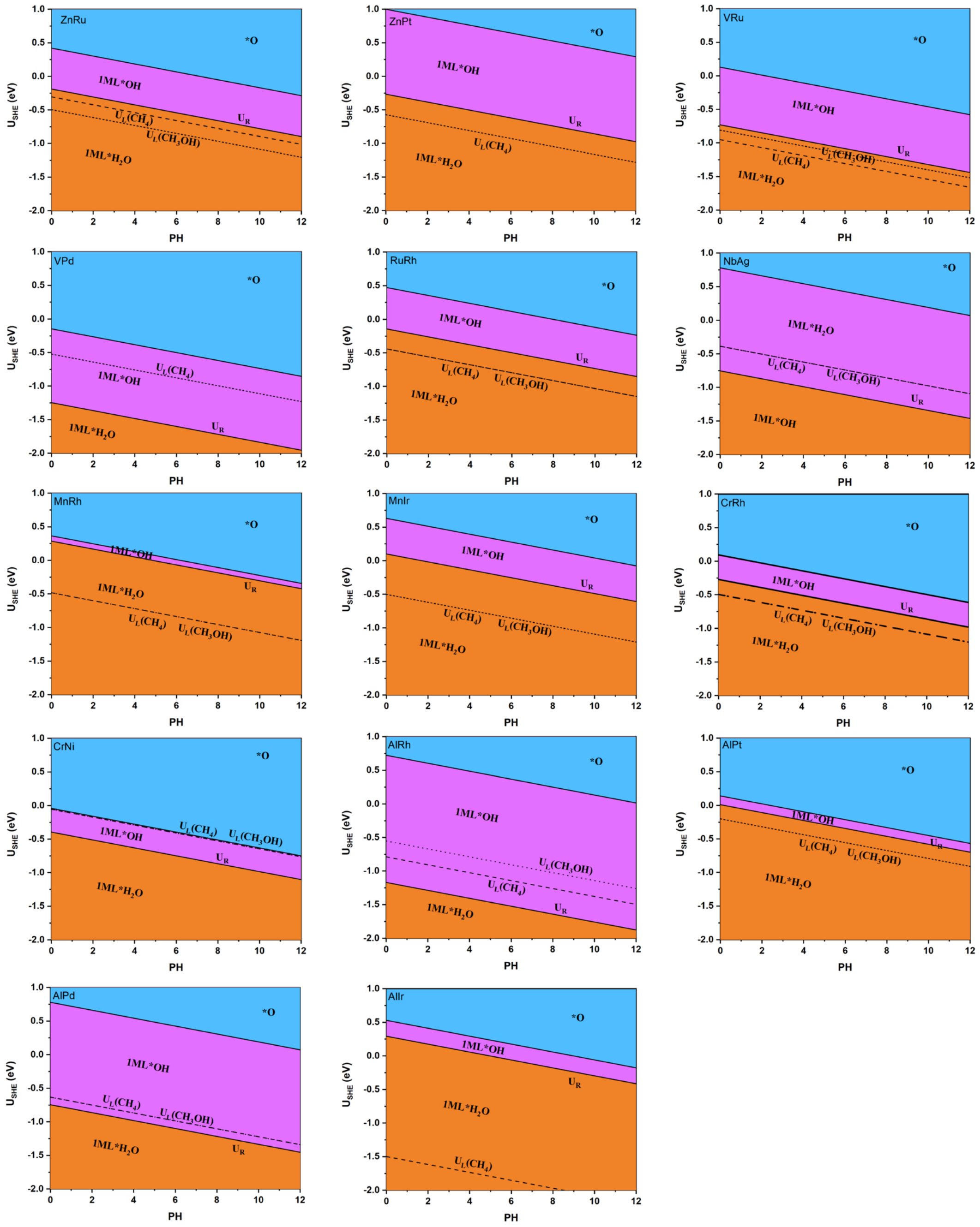

Figure S22. Surface Pourbaix diagrams on the 14 M1M2-N6@Gra (M1M2= ZnRu, ZnPt, VRu, VPd, RuRh, NbAg, MnRh, MnIr, CrRh, CrNi, AlRh, AlIr, AlPd and AlPt). The thermodynamically stable states of the surface under relevant reversible hydrogen electrode (RHE) and pH values are highlighted by blue (for *O), pink (for *OH), and orange (for *H_2O), respectively.

Table S1. Calculated structural parameters (M1-M2 bond length (Å), M1 - N_i (i = 1, 2, 3) bond length (Å) and M2 - N_j (j = 4 ,5, and 6) bond length (Å)).

Metal	d_{M1-M2}	d_{M1-N1}	d_{M1-N2}	d_{M1-N3}	d_{M2-N4}	d_{M2-N5}	d_{M2-N6}
AlSc	2.74	1.88	2.66	1.92	2.05	2.15	2.20
AlTi	2.62	1.89	2.60	1.90	2.00	2.09	2.20
AlV	2.55	1.91	2.45	1.90	1.95	2.05	2.12
AlCr	2.56	1.92	2.23	1.94	1.97	2.02	2.01
AlMn	2.45	1.89	1.97	1.92	1.99	1.95	2.02
AlFe	2.36	1.89	1.98	1.90	1.93	1.91	2.00
AlCo	2.38	1.92	1.97	1.90	1.90	1.90	1.96
AlNi	2.31	1.96	1.94	1.93	1.94	1.85	1.97
AlCu	2.43	1.88	1.92	1.91	1.93	1.87	1.95
AlZn	2.47	1.94	2.06	1.95	2.06	2.23	2.04
AlGa	2.64	1.88	1.96	1.90	2.07	2.34	2.03
AlY	2.85	1.87	2.71	1.90	2.19	2.31	2.29
AlZr	2.76	1.86	2.70	1.89	2.11	2.20	2.26
AlNb	2.60	1.88	2.59	1.88	2.05	2.14	2.20
AlMo	2.49	1.85	2.43	1.84	2.00	2.10	2.17
AlRu	2.32	1.87	2.11	1.83	1.93	2.07	2.10
AlRh	2.41	1.89	2.13	1.88	1.94	2.04	2.05
AlPd	2.31	1.92	1.99	1.95	2.05	1.95	2.12
AlAg	2.52	1.95	2.02	1.94	2.44	2.53	2.49
AlSn	2.86	1.97	2.04	1.95	2.30	2.77	2.38
AlHf	2.77	1.86	2.74	1.90	2.07	2.16	2.23
AlTa	2.61	1.87	2.65	1.87	2.05	2.10	2.21
AlW	2.50	1.85	2.47	1.84	2.00	2.08	2.15
AlRe	2.40	1.87	2.28	1.84	1.96	2.07	2.09
AlOs	2.34	1.87	2.15	1.82	1.93	2.06	2.07
AlIr	2.42	1.88	2.17	1.87	1.94	2.04	2.02
AlPt	2.32	1.92	1.99	1.96	2.03	1.95	2.12
AlAu	2.37	1.85	1.95	1.93	2.03	1.94	2.11
AlBi	2.89	1.98	2.00	1.93	2.30	2.96	2.50
ScTi	2.71	2.10	2.20	2.08	1.99	2.11	2.13
ScV	2.63	2.07	2.23	2.07	1.93	2.06	2.08
ScCr	2.74	2.08	2.15	2.09	1.95	2.01	1.98
ScMn	2.60	2.21	2.14	2.09	1.96	1.99	1.99
ScFe	2.45	2.22	2.16	2.09	1.90	1.97	1.98
ScCo	2.35	2.28	2.14	2.10	1.86	1.97	1.93
ScNi	2.32	2.25	2.11	2.10	1.90	1.91	1.97
ScCu	2.55	2.10	2.11	2.08	1.90	1.92	1.94
ScZn	2.71	2.31	2.10	2.07	2.26	2.90	2.19
ScGa	2.74	2.19	2.13	2.05	2.03	2.73	1.99
ScY	3.01	2.05	2.14	2.07	2.31	2.35	2.15
ScZr	2.83	2.05	2.15	2.14	2.20	2.17	2.11
ScNb	2.72	2.08	2.15	2.14	2.13	2.15	2.07

ScMo	2.53	2.23	2.20	2.10	1.98	2.12	2.15
ScRu	2.39	2.28	2.23	2.09	1.92	2.09	2.04
ScRh	2.44	2.23	2.18	2.07	1.92	2.08	2.03
ScPd	2.39	2.16	2.15	2.14	2.03	1.97	2.13
ScAg	2.62	2.01	2.12	2.16	2.06	2.00	2.14
ScSn	3.06	2.33	2.10	2.06	2.26	3.29	2.27
ScHf	2.84	2.05	2.16	2.14	2.14	2.12	2.06
ScTa	2.70	2.11	2.23	2.12	2.02	2.09	2.18
ScW	2.57	2.19	2.23	2.11	1.97	2.09	2.13
ScRe	2.50	2.20	2.29	2.11	1.94	2.06	2.07
ScOs	2.45	2.25	2.27	2.09	1.93	2.08	2.02
ScIr	2.51	2.18	2.21	2.07	1.92	2.07	2.01
ScPt	2.42	2.17	2.15	2.17	2.02	1.97	2.15
ScAu	2.58	2.04	2.11	2.17	2.03	1.97	2.15
ScBi	3.06	2.36	2.10	2.07	2.29	3.34	2.38
TiV	2.50	2.06	2.14	1.98	1.92	2.07	2.02
TiCr	2.73	1.97	2.07	2.06	1.94	2.00	1.96
TiMn	2.39	2.18	2.09	2.01	1.97	2.00	2.05
TiFe	2.10	2.28	2.05	2.08	1.94	1.93	2.07
TiCo	2.02	2.28	2.01	2.07	1.91	1.92	2.04
TiNi	2.24	2.19	2.04	2.02	1.88	1.93	1.96
TiCu	2.45	2.08	2.08	1.98	1.88	1.94	1.93
TiZn	2.60	2.24	2.07	2.01	2.11	2.71	2.11
TiGa	2.64	2.21	2.09	2.00	2.03	2.69	2.03
TiY	2.85	1.98	2.08	2.09	2.26	2.28	2.21
TiZr	2.64	2.02	2.08	2.11	2.19	2.17	2.15
TiNb	2.23	2.11	2.03	2.16	2.17	2.06	2.14
TiMo	2.30	2.20	2.14	2.01	2.01	2.12	2.19
TiRu	2.11	2.26	2.06	2.06	1.98	2.04	2.10
TiRh	2.10	2.20	2.04	2.06	2.01	1.99	2.15
TiPd	2.31	2.09	2.07	2.04	2.01	1.98	2.11
TiAg	2.57	1.95	2.05	2.11	2.06	2.00	2.14
TiSn	2.94	2.30	2.05	2.00	2.24	3.02	2.32
TiHf	2.67	2.02	2.06	2.11	2.15	2.12	2.12
TiTa	2.42	2.10	2.04	2.14	2.14	2.06	2.13
TiW	2.36	2.15	2.15	2.04	1.99	2.07	2.18
TiRe	2.27	2.20	2.17	2.03	1.97	2.07	2.14
TiOs	2.22	2.24	2.08	2.05	1.95	2.04	2.06
TiIr	2.18	2.18	2.05	2.09	2.00	1.97	2.14
TiPt	2.33	2.09	2.05	2.07	2.00	1.97	2.11
TiAu	2.54	1.97	2.02	2.13	2.03	1.96	2.14
TiBi	2.86	2.33	2.06	2.03	2.31	3.15	2.44
VCr	2.63	1.99	2.07	1.94	1.94	2.04	1.95
VMn	2.61	1.97	2.06	1.97	1.94	1.99	1.92
VFe	2.24	2.10	2.05	1.92	1.83	2.04	1.93
VCo	2.08	2.16	2.00	1.96	1.89	1.94	2.02
VNi	2.23	2.11	2.03	1.93	1.86	1.97	1.94
VCu	2.47	2.00	2.03	1.95	1.91	1.92	1.95

VZn	2.58	2.13	2.05	1.94	2.05	2.51	2.07
VGa	2.65	2.10	2.06	1.94	2.05	2.60	2.07
VY	2.76	2.06	2.07	1.93	2.21	2.35	2.21
VZr	2.50	2.11	2.09	1.93	2.12	2.24	2.29
VNb	2.37	2.13	2.09	1.94	2.02	2.18	2.21
VMo	2.38	2.00	2.12	1.95	2.00	2.12	2.10
VRu	1.94	2.20	1.98	2.02	2.03	2.00	2.21
VRh	2.09	2.11	2.02	1.97	2.00	2.00	2.15
VPd	2.29	2.03	2.06	1.95	1.99	2.00	2.09
VAg	2.78	2.09	2.05	1.94	2.30	2.64	2.36
VSn	2.82	2.13	2.05	1.95	2.19	2.73	2.23
VHf	2.67	2.04	2.07	1.93	2.09	2.18	2.09
VTa	2.35	2.12	2.10	1.95	2.03	2.14	2.21
VW	1.77	2.18	1.93	2.11	2.11	1.98	2.15
VRe	1.92	2.19	1.95	2.05	2.04	1.98	2.14
VOs	2.02	2.19	1.98	2.03	2.01	1.98	2.18
VIr	2.14	2.11	2.00	1.99	1.98	1.99	2.12
VPt	2.28	2.04	2.06	1.95	1.97	2.00	2.10
VAu	2.48	1.97	2.06	2.01	2.02	1.97	2.15
VBi	2.80	2.22	2.06	1.98	2.26	2.99	2.37
CrMn	2.55	1.94	2.00	1.94	1.93	1.99	1.91
CrFe	2.21	2.05	2.00	1.94	1.90	1.97	2.02
CrCo	2.25	2.05	2.01	1.92	1.86	1.97	1.96
CrNi	2.37	2.03	2.00	1.95	1.89	1.93	1.94
CrCu	2.43	1.98	1.99	1.94	1.91	1.92	1.94
CrZn	2.55	1.94	2.04	1.90	1.89	1.99	1.88
CrGa	2.91	2.04	2.03	1.92	2.12	2.67	2.13
CrY	2.92	1.98	2.04	1.93	2.23	2.30	2.19
CrZr	2.54	2.01	2.08	1.91	2.12	2.23	2.29
CrNb	2.39	2.03	2.06	1.94	2.04	2.14	2.26
CrMo	2.34	2.04	2.04	1.95	1.99	2.10	2.16
CrRu	2.26	1.99	2.06	1.90	1.91	2.09	2.04
CrRh	2.45	1.99	2.06	1.94	1.92	2.06	2.01
CrPd	2.38	1.99	2.01	1.96	2.01	2.01	2.09
CrAg	2.75	2.02	2.03	1.93	2.26	2.43	2.31
CrSn	2.87	2.10	2.04	1.94	2.21	2.79	2.30
CrHf	2.57	1.97	2.07	1.95	2.09	2.14	2.25
CrTa	2.40	2.02	2.07	1.96	2.03	2.10	2.22
CrW	2.39	2.02	2.06	1.96	1.98	2.07	2.14
CrRe	2.27	1.99	2.07	1.89	1.93	2.10	2.04
CrOs	2.30	1.99	2.08	1.89	1.91	2.09	2.00
CrIr	2.47	2.00	2.06	1.95	1.93	2.05	1.99
CrPt	2.37	2.00	2.01	1.98	1.99	1.98	2.09
CrAu	2.43	1.95	2.01	1.99	2.03	1.99	2.13
CrBi	2.84	2.11	2.03	1.94	2.29	2.96	2.52
MnFe	2.36	1.98	1.95	1.93	1.88	1.96	1.97
MnCo	2.24	2.07	1.93	1.97	1.91	1.90	1.97
MnNi	2.30	2.04	1.92	1.95	1.90	1.90	1.95

MnCu	2.43	1.93	1.97	1.91	1.91	1.91	1.95
MnZn	2.55	1.95	1.99	1.97	1.93	1.99	1.93
MnGa	2.66	2.09	1.98	1.99	2.11	2.54	2.10
MnY	2.73	1.98	1.99	1.97	2.22	2.26	2.33
MnZr	2.53	2.02	2.01	1.97	2.12	2.17	2.28
MnNb	2.42	2.03	2.02	1.96	2.04	2.11	2.23
MnMo	1.78	2.19	1.94	2.00	2.14	2.02	2.37
MnRu	2.10	2.02	2.01	1.90	1.95	2.04	2.12
MnRh	2.37	1.97	1.96	1.94	1.94	2.02	2.01
MnPd	2.32	1.99	1.95	1.95	1.99	1.97	2.07
MnAg	2.42	1.89	1.99	1.92	2.02	1.98	2.09
MnSn	2.74	2.05	1.94	1.98	2.28	2.44	2.31
MnHf	2.51	2.00	2.02	1.96	2.09	2.14	2.26
MnTa	2.42	2.02	2.04	1.96	2.03	2.08	2.21
MnW	1.92	2.15	1.92	1.99	2.10	2.00	2.31
MnRe	2.26	1.97	2.06	1.87	1.92	2.08	2.06
MnOs	2.13	2.04	2.00	1.90	1.94	2.02	2.08
MnIr	2.30	2.03	1.97	1.96	1.96	1.99	2.04
MnPt	2.32	1.99	1.94	1.96	1.98	1.97	2.07
MnAu	2.40	1.90	1.97	1.93	2.00	1.97	2.10
MnBi	2.68	1.98	1.95	1.96	2.33	2.42	2.41
FeCo	2.18	2.06	1.91	1.93	1.90	1.90	2.00
FeNi	2.32	1.99	1.93	1.91	1.90	1.90	1.96
FeCu	2.43	1.91	1.95	1.88	1.92	1.89	1.96
FeZn	2.49	1.91	2.01	1.85	1.93	2.03	1.96
FeGa	2.53	1.97	1.97	1.90	2.08	2.32	2.09
FeY	2.59	1.98	1.98	1.90	2.22	2.28	2.34
FeZr	2.36	1.99	1.97	1.90	2.14	2.17	2.31
FeNb	2.11	2.03	1.94	1.92	2.11	2.07	2.32
FeMo	2.03	2.04	1.95	1.92	2.05	2.03	2.23
FeRu	2.18	1.97	2.01	1.86	1.94	2.03	2.10
FeRh	2.25	2.01	1.95	1.92	1.96	1.99	2.05
FePd	2.34	1.95	1.96	1.91	1.98	1.97	2.06
FeAg	2.42	1.87	1.98	1.89	2.03	1.98	2.11
FeSn	2.66	2.02	1.95	1.93	2.25	2.42	2.30
FeHf	2.34	1.99	1.97	1.90	2.11	2.13	2.28
FeTa	2.01	2.07	1.91	1.95	2.13	2.04	2.35
FeW	2.03	2.06	1.93	1.93	2.04	2.00	2.22
FeRe	2.10	2.03	1.99	1.88	1.96	2.02	2.11
FeOs	2.17	1.98	2.01	1.85	1.94	2.03	2.07
FeIr	2.26	2.01	1.94	1.93	1.95	1.98	2.04
FePt	2.33	1.96	1.95	1.92	1.97	1.97	2.05
FeAu	2.39	1.88	1.95	1.91	2.01	1.96	2.11
FeBi	2.56	1.98	1.94	1.92	2.34	2.40	2.47
CoNi	2.34	1.95	1.88	1.90	1.91	1.86	1.98
CoCu	2.43	1.87	1.93	1.85	1.92	1.89	1.98
CoZn	2.49	1.88	2.00	1.83	1.91	1.97	1.95
CoGa	2.77	1.88	1.98	1.83	2.13	2.44	2.17

CoY	2.51	1.92	1.97	1.86	2.23	2.27	2.39
CoZr	2.24	1.97	1.93	1.89	2.16	2.14	2.36
CoNb	2.18	1.96	1.94	1.88	2.07	2.06	2.27
CoMo	2.14	1.97	1.98	1.86	2.00	2.05	2.19
CoRu	2.26	1.92	2.02	1.83	1.93	2.05	2.08
CoRh	2.32	1.95	1.96	1.87	1.94	2.00	2.04
CoPd	2.37	1.92	1.92	1.90	1.99	1.96	2.07
CoAg	2.40	1.84	1.96	1.87	2.03	1.97	2.12
CoSn	2.63	1.95	1.93	1.89	2.28	2.36	2.37
CoHf	2.16	1.98	1.92	1.90	2.14	2.09	2.36
CoTa	2.15	1.98	1.93	1.90	2.07	2.04	2.26
CoW	2.15	1.98	1.97	1.86	1.99	2.03	2.16
CoRe	2.19	1.95	2.01	1.84	1.94	2.04	2.08
CoOs	2.26	1.92	2.04	1.82	1.92	2.04	2.04
CoIr	2.31	1.97	1.95	1.89	1.94	2.00	2.02
CoPt	2.37	1.93	1.91	1.92	1.98	1.96	2.06
CoAu	2.39	1.85	1.93	1.89	2.01	1.95	2.13
CoBi	2.54	1.94	1.92	1.90	2.34	2.33	2.47
NiCu	2.41	1.91	1.92	1.88	1.92	1.90	1.98
NiZn	2.46	1.91	1.96	1.88	1.93	1.97	1.97
NiGa	2.30	1.94	1.89	1.93	2.01	2.03	2.05
NiY	2.48	1.95	1.91	1.90	2.24	2.24	2.36
NiZr	2.37	1.93	1.93	1.88	2.14	2.13	2.27
NiNb	2.31	1.92	1.96	1.86	2.03	2.09	2.20
NiMo	2.32	1.91	1.98	1.85	1.99	2.07	2.14
NiRu	2.34	1.94	1.97	1.88	1.94	2.03	2.03
NiRh	2.41	1.91	1.97	1.86	1.92	2.02	2.01
NiPd	2.37	1.92	1.89	1.91	1.99	1.95	2.05
NiAg	2.39	1.87	1.95	1.89	2.02	1.96	2.10
NiSn	2.67	1.94	1.93	1.89	2.27	2.32	2.32
NiHf	2.33	1.94	1.92	1.88	2.10	2.10	2.25
NiTa	2.28	1.93	1.96	1.86	2.03	2.06	2.19
NiW	2.26	1.90	2.01	1.84	1.96	2.05	2.12
NiRe	2.33	1.95	1.94	1.90	1.98	2.02	2.06
NiOs	2.34	1.95	1.96	1.88	1.92	2.02	2.01
NiIr	2.40	1.95	1.92	1.92	1.94	1.98	2.00
NiPt	2.38	1.92	1.88	1.92	1.97	1.95	2.05
NiAu	2.38	1.87	1.92	1.90	2.00	1.95	2.10
NiBi	2.50	1.93	1.90	1.93	2.32	2.29	2.43
CuZn	2.41	1.96	1.91	1.93	1.95	1.97	1.98
CuGa	2.48	2.00	1.92	1.95	2.12	2.23	2.13
CuY	2.66	1.93	1.92	1.90	2.21	2.23	2.24
CuZr	2.56	1.92	1.94	1.89	2.11	2.15	2.17
CuNb	2.57	1.95	1.93	1.91	2.05	2.13	2.08
CuMo	2.48	1.93	1.96	1.90	2.02	2.08	2.07
CuRu	2.43	1.93	1.94	1.92	1.94	2.01	1.99
CuRh	2.43	1.95	1.92	1.92	1.93	1.99	1.98
CuPd	2.42	1.93	1.91	1.92	1.96	1.99	2.01

CuAg	2.57	1.98	1.95	1.93	2.25	2.24	2.33
CuSn	2.64	1.98	1.91	1.94	2.25	2.32	2.27
CuHf	2.53	1.92	1.95	1.89	2.08	2.12	2.14
CuTa	2.45	1.89	1.97	1.88	2.00	2.09	2.13
CuW	2.47	1.92	1.96	1.91	1.99	2.05	2.05
CuRe	2.42	1.91	1.98	1.89	1.95	2.04	2.01
CuOs	2.44	1.93	1.94	1.93	1.92	2.01	1.96
CuIr	2.43	1.94	1.92	1.93	1.93	1.98	1.97
CuPt	2.42	1.93	1.91	1.93	1.96	1.99	2.00
CuAu	2.40	1.90	1.98	1.94	2.02	1.98	2.12
CuBi	2.65	1.95	1.92	1.94	2.28	2.30	2.29
ZnGa	2.78	2.01	1.96	1.94	2.13	2.50	2.11
ZnY	2.88	2.21	3.13	2.45	2.22	2.22	2.42
ZnZr	2.74	2.14	2.94	2.27	2.12	2.13	2.34
ZnNb	2.62	2.08	2.75	2.10	2.03	2.09	2.26
ZnMo	2.55	2.02	2.58	2.01	2.01	2.09	2.18
ZnRu	2.47	1.97	2.20	1.94	1.93	2.07	2.03
ZnRh	2.45	1.92	2.03	1.90	1.91	2.07	1.99
ZnPd	2.41	1.93	2.02	1.93	1.98	2.01	2.05
ZnAg	2.57	1.96	1.98	1.94	2.20	2.18	2.27
ZnSn	2.97	1.94	1.95	1.91	2.21	2.41	2.16
ZnHf	2.71	2.13	2.90	2.23	2.08	2.09	2.32
ZnTa	2.62	2.06	2.73	2.10	2.02	2.07	2.23
ZnW	2.55	2.03	2.60	2.02	2.00	2.07	2.16
ZnRe	2.50	1.98	2.45	1.96	1.97	2.08	2.10
ZnOs	2.48	1.99	2.27	1.96	1.92	2.04	2.01
ZnIr	2.46	1.91	2.05	1.89	1.92	2.06	1.98
ZnPt	2.42	1.96	2.09	1.97	1.98	2.00	2.05
ZnAu	2.41	1.95	2.09	1.99	2.07	2.01	2.17
ZnBi	2.69	2.02	2.10	2.03	2.34	2.49	2.47
GaY	2.85	1.98	2.80	2.03	2.18	2.27	2.29
GaZr	2.75	1.98	2.78	2.02	2.11	2.18	2.26
GaNb	2.63	2.00	2.69	2.01	2.05	2.14	2.21
GaMo	2.71	2.12	2.75	2.08	2.01	2.08	2.18
GaRu	2.51	2.10	2.48	2.02	1.93	2.06	2.05
GaRh	2.73	2.23	2.57	2.15	1.92	2.04	2.01
GaPd	2.34	2.00	2.12	2.03	2.05	1.99	2.07
GaAg	2.47	2.09	2.34	2.11	2.34	2.42	2.37
GaSn	2.85	2.04	2.33	2.08	2.29	2.48	2.28
GaHf	2.78	1.99	2.79	2.04	2.07	2.13	2.21
GaTa	2.63	1.99	2.72	2.00	2.04	2.10	2.21
GaW	2.55	1.98	2.61	1.97	2.00	2.09	2.15
GaRe	2.46	2.03	2.50	1.97	1.95	2.07	2.09
GaOs	2.54	2.09	2.51	2.02	1.93	2.06	2.02
GaIr	2.75	2.26	2.61	2.15	1.92	2.04	1.99
GaPt	2.34	2.00	2.13	2.04	2.03	1.99	2.07
GaAu	2.42	2.01	2.18	2.09	2.07	1.99	2.15
GaBi	2.79	2.06	2.10	2.02	2.31	2.97	2.51

YZr	2.96	2.20	2.29	2.29	2.22	2.17	2.10
YNb	2.85	2.22	2.29	2.28	2.14	2.13	2.06
YMo	2.67	2.36	2.33	2.23	1.97	2.12	2.14
YRu	2.54	2.39	2.36	2.24	1.92	2.09	2.02
YRh	2.62	2.31	2.31	2.21	1.91	2.09	2.01
YPd	2.53	2.29	2.29	2.29	2.04	1.99	2.14
YAg	2.73	2.15	2.25	2.28	2.07	2.02	2.14
YSn	3.20	2.40	2.23	2.21	2.27	3.51	2.25
YTa	2.77	2.30	2.35	2.25	2.00	2.12	2.18
YW	2.70	2.32	2.35	2.25	1.96	2.09	2.12
YRe	2.65	2.34	2.41	2.24	1.94	2.08	2.05
YOs	2.60	2.37	2.39	2.24	1.93	2.07	2.01
YIr	2.66	2.29	2.33	2.22	1.92	2.07	2.00
YPt	2.57	2.30	2.29	2.31	2.03	1.98	2.15
YAu	2.70	2.18	2.25	2.30	2.04	1.99	2.16
YBi	3.20	2.43	2.23	2.22	2.32	3.56	2.37
ZrMo	2.48	2.28	2.25	2.12	2.00	2.13	2.16
ZrRu	2.37	2.34	2.22	2.13	1.92	2.10	2.04
ZrRh	2.32	2.29	2.18	2.18	2.01	2.00	2.11
ZrPd	2.47	2.16	2.15	2.18	2.01	1.98	2.10
ZrAg	2.68	2.05	2.14	2.20	2.05	1.99	2.12
ZrSn	3.06	2.37	2.10	2.09	2.27	3.12	2.33
ZrHf	2.78	2.14	2.16	2.23	2.18	2.12	2.11
ZrTa	2.54	2.19	2.15	2.25	2.16	2.07	2.10
ZrW	2.52	2.24	2.26	2.15	1.99	2.08	2.16
ZrRe	2.44	2.27	2.30	2.15	1.97	2.06	2.11
ZrOs	2.43	2.32	2.23	2.14	1.93	2.08	2.03
ZrIr	2.36	2.28	2.18	2.21	2.01	1.98	2.11
ZrPt	2.48	2.17	2.15	2.20	2.01	1.97	2.11
ZrAu	2.65	2.07	2.12	2.23	2.03	1.97	2.13
ZrBi	2.98	2.41	2.11	2.12	2.40	3.42	2.58
NbMo	2.36	2.23	2.19	2.03	2.00	2.14	2.16
NbRu	2.18	2.26	2.09	2.11	2.00	2.01	2.12
NbRh	2.26	2.17	2.08	2.07	1.99	2.00	2.08
NbPd	2.40	2.12	2.14	2.03	1.98	2.01	2.07
NbAg	2.65	2.35	2.05	2.04	4.16	3.58	4.69
NbSn	2.94	2.31	2.07	2.04	2.26	3.11	2.34
NbHf	2.62	2.09	2.08	2.17	2.18	2.11	2.15
NbTa	2.36	2.17	2.07	2.19	2.18	2.06	2.16
NbW	2.36	2.19	2.19	2.10	2.02	2.07	2.22
NbRe	2.27	2.26	2.14	2.10	2.00	2.04	2.13
NbOs	2.22	2.25	2.10	2.13	2.01	1.99	2.13
NbIr	2.29	2.17	2.08	2.10	1.99	1.99	2.09
NbPt	2.40	2.12	2.14	2.06	1.98	2.00	2.08
NbAu	2.60	2.06	2.12	2.13	2.02	1.97	2.13
NbBi	2.83	2.35	2.08	2.05	2.37	3.20	2.54
MoRu	2.07	2.18	2.06	2.05	2.01	2.02	2.15
MoRh	2.20	2.11	2.10	1.99	1.96	2.04	2.08

MoPd	2.44	2.07	2.10	2.05	2.01	1.99	2.07
MoAg	2.54	2.04	2.13	2.06	2.04	2.04	2.11
MoSn	2.84	2.21	2.08	2.02	2.22	2.95	2.32
MoHf	2.43	2.17	2.13	2.01	2.09	2.21	2.24
MoTa	2.33	2.17	2.14	2.00	2.03	2.16	2.22
MoW	2.10	2.24	2.09	2.09	2.08	2.03	2.26
MoRe	2.06	2.24	2.06	2.11	2.05	1.99	2.21
MoOs	2.13	2.19	2.05	2.08	2.00	1.99	2.12
MoIr	2.24	2.12	2.10	2.01	1.96	2.03	2.07
MoPt	2.44	2.07	2.09	2.07	1.99	1.98	2.08
MoAu	2.54	2.05	2.11	2.08	2.02	2.00	2.13
MoBi	2.75	2.24	2.09	2.03	2.31	3.06	2.51
RuRh	2.34	2.02	2.07	1.94	1.94	2.04	2.02
RuPd	2.47	1.98	2.05	1.96	2.00	1.99	2.05
RuAg	2.74	2.00	2.06	1.92	2.33	2.54	2.41
RuSn	2.68	2.06	2.06	1.94	2.25	2.64	2.39
RuHf	2.29	2.06	2.07	1.95	2.12	2.16	2.34
RuTa	2.09	2.16	2.00	2.04	2.13	2.06	2.29
RuW	2.12	2.14	2.01	2.03	2.04	2.03	2.16
RuRe	2.16	2.11	2.04	1.96	1.96	2.05	2.07
RuOs	2.23	2.06	2.07	1.93	1.92	2.06	2.01
RuIr	2.30	2.01	2.09	1.91	1.91	2.07	1.99
RuPt	2.46	1.99	2.05	1.98	1.99	1.98	2.05
RuAu	2.49	1.97	2.04	1.98	2.02	2.00	2.14
RuBi	2.57	2.09	2.06	1.96	2.31	2.71	2.51
RhPd	2.46	1.98	2.03	1.96	2.00	2.02	2.05
RhAg	2.74	1.99	2.04	1.92	2.31	2.45	2.37
RhSn	2.71	2.03	2.06	1.93	2.24	2.51	2.32
RhHf	2.22	2.13	1.99	2.02	2.15	2.12	2.29
RhTa	2.22	2.10	1.99	2.01	2.07	2.05	2.17
RhW	2.22	2.09	2.02	1.97	1.99	2.07	2.09
RhRe	2.24	2.05	2.07	1.94	1.92	2.08	2.01
RhOs	2.30	2.02	2.08	1.92	1.90	2.08	1.97
RhIr	2.44	2.01	2.02	1.98	1.96	2.00	1.99
RhPt	2.45	1.99	2.04	1.98	1.98	2.00	2.03
RhAu	2.45	1.96	2.00	1.97	2.02	1.98	2.11
RhBi	2.59	2.05	2.00	1.99	2.34	2.39	2.41
PdAg	2.43	1.97	2.01	1.97	2.02	1.99	2.05
PdSn	2.61	2.10	1.99	2.02	2.29	2.37	2.30
PdHf	2.41	2.11	1.98	2.03	2.13	2.13	2.15
PdTa	2.36	2.09	2.01	2.00	2.03	2.11	2.10
PdW	2.43	2.09	1.98	2.01	2.03	2.07	2.04
PdRe	2.41	2.07	2.00	2.00	1.98	2.05	2.00
PdOs	2.46	2.07	1.99	2.01	1.95	2.03	1.97
PdIr	2.45	2.05	2.01	2.01	1.95	2.01	1.97
PdPt	2.41	2.02	1.96	1.99	1.97	1.96	2.02
PdAu	2.42	1.97	1.98	1.98	2.00	1.97	2.06
PdBi	2.57	2.08	1.98	2.04	2.34	2.35	2.38

AgSn	2.64	2.19	2.04	2.10	2.26	2.46	2.22
AgHf	2.64	2.13	2.00	2.06	2.16	2.12	2.03
AgTa	2.61	2.11	1.97	2.04	2.11	2.08	1.99
AgW	2.53	2.09	2.03	2.04	2.03	2.09	2.01
AgRe	2.73	2.47	2.72	2.38	1.96	2.05	2.08
AgOs	2.75	2.42	2.57	2.34	1.91	2.05	1.97
AgIr	2.73	2.37	2.49	2.32	1.92	2.04	1.97
AgPt	2.44	2.04	1.98	2.02	1.96	2.00	1.97
AgAu	2.66	2.34	2.56	2.33	2.07	2.02	2.23
AgBi	2.68	2.15	2.05	2.09	2.31	2.42	2.28
SnHf	3.04	2.34	3.20	2.28	2.07	2.08	2.36
SnTa	2.95	2.33	3.12	2.26	2.03	2.05	2.28
SnW	2.85	2.34	2.98	2.23	2.01	2.06	2.19
SnRe	2.80	2.35	2.85	2.22	1.97	2.06	2.13
SnOs	2.71	2.41	2.70	2.25	1.93	2.05	2.04
SnIr	2.73	2.35	2.58	2.24	1.93	2.05	2.01
SnPt	2.63	2.32	2.36	2.32	2.00	1.98	2.08
SnAu	2.65	2.28	2.41	2.31	2.06	1.98	2.19
SnBi	2.98	2.27	2.48	2.29	2.31	2.44	2.38
HfTa	2.65	2.14	2.11	2.18	2.14	2.07	2.09
HfW	2.50	2.19	2.21	2.11	1.99	2.08	2.16
HfRe	2.40	2.24	2.23	2.11	1.97	2.06	2.12
HfOs	2.37	2.28	2.18	2.09	1.92	2.09	2.04
HfIr	2.30	2.26	2.13	2.18	2.01	1.98	2.12
HfPt	2.43	2.13	2.10	2.16	2.01	1.96	2.12
HfAu	2.61	2.05	2.09	2.18	2.03	1.97	2.14
HfBi	2.96	2.38	2.09	2.09	2.40	3.38	2.63
TaW	2.35	2.18	2.16	2.07	2.02	2.08	2.21
TaRe	2.22	2.28	2.09	2.11	2.02	2.01	2.14
TaOs	2.18	2.26	2.08	2.13	2.03	1.99	2.15
TaIr	2.26	2.16	2.06	2.10	2.01	1.98	2.10
TaPt	2.38	2.10	2.10	2.06	1.99	1.99	2.10
TaAu	2.60	2.00	2.03	2.15	2.03	1.96	2.12
TaBi	2.80	2.34	2.07	2.05	2.40	3.24	2.71
WRe	2.13	2.22	2.04	2.09	2.05	2.00	2.19
WOs	2.17	2.16	2.03	2.07	2.03	1.99	2.13
WIr	2.25	2.10	2.07	2.01	1.97	2.01	2.09
WPt	2.44	2.05	2.07	2.04	2.00	1.98	2.08
WAu	2.53	2.02	2.07	2.04	2.02	1.99	2.11
WBi	2.75	2.22	2.07	2.02	2.32	3.08	2.54
ReOs	2.20	2.10	2.05	1.98	1.96	2.02	2.09
ReIr	2.27	2.03	2.08	1.93	1.93	2.05	2.04
RePt	2.42	2.01	2.05	2.00	1.98	1.99	2.06
ReAu	2.50	2.00	2.06	2.01	2.01	1.99	2.09
ReBi	2.62	2.14	2.07	1.98	2.35	2.89	2.55
OsIr	2.30	1.98	2.09	1.91	1.92	2.07	2.00
OsPt	2.45	1.98	2.03	1.97	1.99	1.98	2.06
OsAu	2.50	1.96	2.03	1.97	2.02	1.98	2.11

OsBi	2.60	2.07	2.06	1.96	2.33	2.81	2.53
IrPt	2.45	1.98	2.02	1.97	1.99	2.00	2.04
IrAu	2.46	1.95	1.98	1.96	2.03	1.99	2.10
IrBi	2.65	2.02	2.02	1.97	2.28	2.83	2.45
PtAu	2.44	1.98	1.98	1.97	2.00	1.97	2.05
PtBi	2.58	2.08	1.97	2.03	2.36	2.35	2.40
AuBi	2.67	2.12	1.99	2.05	2.31	2.37	2.28
AlAl	2.57	1.89	2.05	1.94	1.94	2.05	1.90
ScSc	2.87	2.02	2.18	2.11	2.11	2.18	2.02
TiTi	2.45	2.16	2.12	2.01	2.00	2.12	2.16
VV	2.44	1.98	2.11	1.92	1.91	2.06	2.00
CrCr	2.56	1.95	2.01	1.96	1.96	2.01	1.95
MnMn	2.43	1.98	1.97	1.95	1.93	1.96	1.97
FeFe	2.22	2.02	1.92	1.92	1.92	1.91	2.02
CoCo	2.26	1.99	1.92	1.88	1.88	1.92	1.99
NiNi	2.37	1.95	1.87	1.90	1.90	1.87	1.95
CuCu	2.40	1.98	1.93	1.93	1.93	1.92	1.98
ZnZn	2.57	1.91	1.95	1.92	1.92	1.95	1.91
GaGa	2.53	1.98	2.14	2.03	2.03	2.15	1.99
YY	3.23	2.17	2.31	2.30	2.30	2.32	2.17
ZrZr	2.75	2.14	2.16	2.22	2.22	2.16	2.14
NbNb	2.16	2.14	2.04	2.18	2.19	2.04	2.14
MoMo	1.78	2.19	2.00	2.15	2.15	2.00	2.19
RuRu	2.22	2.04	2.08	1.93	1.93	2.08	2.04
RhRh	2.46	2.00	2.03	1.96	1.96	2.02	1.99
PdPd	2.40	2.02	1.97	1.99	1.99	1.97	2.02
AgAg	2.62	2.27	2.45	2.29	2.15	2.13	2.24
SnSn	3.04	2.16	2.43	2.24	2.24	2.43	2.16
HfHf	2.83	2.09	2.10	2.17	2.18	2.10	2.09
TaTa	2.19	2.15	2.05	2.18	2.18	2.04	2.15
WW	2.17	2.22	2.05	2.08	2.08	2.03	2.23
ReRe	2.15	2.14	2.00	2.04	2.03	1.99	2.14
OsOs	2.25	2.03	2.06	1.92	1.92	2.06	2.03
IrIr	2.44	2.01	2.00	1.97	1.97	2.00	2.01
PtPt	2.42	2.02	1.96	1.97	1.97	1.96	2.02
AuAu	2.45	2.01	1.98	1.99	2.00	1.98	2.01

Table S2. Computed formation energy (E_f) and dissolution potential (U_{diss}) of dual-metals, and N_e are the standard dissolution potential of bulk metal and the number of transferred electrons involved in the dissolution, respectively. The electrochemically unstable systems are depicted by **red** color. $N_e = [N_e(TM1)+N_e(TM2)]/2$, = /2.

M1M2	$E_{M1}E_{M2}$	Ne	U^0_{diss}	E_f	U_{diss}
AlSc	-633.69	3	-1.87	-2.61	-1.00
AlTi	-633.91	2.5	-1.65	-1.95	-0.87
AlV	-634.52	2.5	-1.42	-1.65	-0.76
AlCr	-634.98	2.5	-1.29	-1.61	-0.64
AlMn	-634.71	2.5	-1.42	-1.71	-0.74
AlFe	-633.64	2.5	-1.06	-1.53	-0.44
AlCo	-632.50	2.5	-0.97	-1.64	-0.32
AlNi	-631.86	2.5	-0.96	-1.98	-0.17
AlCu	-629.70	2.5	-0.66	-1.74	0.04
AlZn	-627.27	2.5	-1.21	-1.94	-0.43
AlGa	-628.91	3	-1.11	-1.87	-0.48
AlY	-633.67	3	-2.02	-2.54	-1.17
AlZr	-634.36	3.5	-1.56	-1.85	-1.03
AlNb	-634.77	3	-1.38	-1.27	-0.96
AlMo	-634.64	3	-0.93	-0.83	-0.65
AlRu	-633.36	2.5	-0.60	-0.98	-0.21
AlRh	-632.18	2.5	-0.53	-1.35	0.01
AlPd	-630.93	2.5	-0.36	-1.82	0.37
AlAg	-627.64	2	-0.43	-1.34	0.24
AlSn	-629.35	2.5	-0.90	-1.61	-0.26
AlHf	-635.78	3.5	-1.61	-1.86	-1.07
AlTa	-636.04	3	-1.13	-1.03	-0.79
AlW	-636.03	3	-0.78	-0.47	-0.62
AlRe	-635.37	3	-0.68	-0.40	-0.55
AlOs	-634.55	5.5	-0.41	-0.60	-0.30
AlIr	-633.17	3	-0.25	-1.09	0.11
AlPt	-631.92	2.5	-0.24	-1.87	0.51
AlAu	-628.16	3	-0.08	-1.07	0.28
AlBi	-628.65	2	-0.58	-1.31	0.08
ScTi	-637.00	2.5	-1.86	-2.19	-0.98
ScV	-637.58	2.5	-1.63	-1.89	-0.87
ScCr	-638.04	2.5	-1.50	-1.84	-0.76
ScMn	-637.82	2.5	-1.64	-1.98	-0.84
ScFe	-636.87	2.5	-1.27	-1.85	-0.52
ScCo	-635.96	2.5	-1.18	-2.07	-0.35
ScNi	-634.98	2.5	-1.17	-2.25	-0.27
ScCu	-632.73	2.5	-0.87	-1.96	-0.09
ScZn	-630.65	2.5	-1.42	-2.33	-0.49
ScGa	-632.66	3	-1.32	-2.46	-0.50
ScY	-635.93	3	-2.23	-2.37	-1.43
ScZr	-636.72	3.5	-1.77	-1.73	-1.27
ScNb	-637.17	3	-1.59	-1.18	-1.20
ScMo	-637.88	3	-1.14	-1.16	-0.75
ScRu	-637.14	2.5	-0.81	-1.58	-0.18
ScRh	-635.73	2.5	-0.74	-1.83	-0.01
ScPd	-633.84	2.5	-0.57	-1.98	0.23
ScAg	-630.38	2	-0.64	-1.42	0.07
ScSn	-632.94	2.5	-1.11	-2.11	-0.27
ScHf	-637.97	3.5	-1.82	-1.66	-1.34
ScTa	-639.06	3	-1.34	-1.24	-0.93
ScW	-639.24	3	-0.99	-0.79	-0.73
ScRe	-638.75	3	-0.89	-0.80	-0.62
ScOs	-638.17	5.5	-0.62	-1.12	-0.42
ScIr	-636.69	3	-0.46	-1.56	0.06
ScPt	-634.79	2.5	-0.45	-2.01	0.35
ScAu	-631.01	3	-0.29	-1.20	0.11
ScBi	-632.65	2	-0.79	-2.02	0.22

TiV	-637.77	2	-1.40	-1.21	-0.80
TiCr	-638.31	2	-1.27	-1.20	-0.67
TiMn	-638.34	2	-1.41	-1.45	-0.68
TiFe	-637.48	2	-1.04	-1.38	-0.35
TiCo	-636.76	2	-0.96	-1.69	-0.11
TiNi	-635.38	2	-0.95	-1.66	-0.11
TiCu	-632.90	2	-0.64	-1.27	-0.01
TiZn	-630.68	2	-1.19	-1.57	-0.41
TiGa	-632.87	2.5	-1.09	-1.78	-0.38
TiY	-636.41	2.5	-2.00	-1.84	-1.27
TiZr	-637.34	3	-1.54	-1.26	-1.12
TiNb	-637.93	2.5	-1.37	-0.77	-1.06
TiMo	-638.54	2.5	-0.91	-0.71	-0.63
TiRu	-637.69	2	-0.59	-1.07	-0.05
TiRh	-636.50	2	-0.51	-1.44	0.20
TiPd	-634.19	2	-0.34	-1.37	0.35
TiAg	-630.57	1.5	-0.41	-0.73	0.07
TiSn	-633.23	2	-0.89	-1.47	-0.15
TiHf	-638.60	3	-1.59	-1.19	-1.19
TiTa	-639.01	2.5	-1.12	-0.44	-0.94
TiW	-639.84	2.5	-0.76	-0.31	-0.64
TiRe	-639.42	2.5	-0.66	-0.36	-0.52
TiOs	-638.70	5	-0.39	-0.60	-0.27
TiIr	-637.62	2.5	-0.24	-1.24	0.26
TiPt	-635.23	2	-0.22	-1.45	0.50
TiAu	-631.18	2.5	-0.06	-0.51	0.14
TiBi	-632.87	1.5	-0.57	-1.35	0.34
VCr	-638.90	2	-1.05	-0.90	-0.60
VMn	-638.16	2	-1.19	-0.77	-0.80
VFe	-637.48	2	-0.82	-0.78	-0.42
VCo	-637.06	2	-0.73	-1.25	-0.11
VNi	-635.78	2	-0.72	-1.27	-0.08
VCu	-633.52	2	-0.42	-0.98	0.07
VZn	-631.13	2	-0.97	-1.20	-0.37
VGa	-633.42	2.5	-0.87	-1.46	-0.28
VY	-637.61	2.5	-1.78	-1.84	-1.04
VZr	-638.51	3	-1.32	-1.25	-0.90
VNb	-638.95	2.5	-1.14	-0.69	-0.86
VMo	-638.06	2.5	-0.69	0.13	-0.74
VRu	-638.27	2	-0.36	-0.76	0.02
VRh	-636.67	2	-0.29	-0.92	0.17
VPd	-634.50	2	-0.11	-0.93	0.35
VAg	-631.65	1.5	-0.19	-0.68	0.26
VSn	-633.80	2	-0.66	-1.16	-0.08
VHf	-639.39	3	-1.37	-0.99	-1.04
VTa	-640.14	2.5	-0.89	-0.41	-0.73
VW	-640.70	2.5	-0.54	-0.14	-0.48
VRe	-639.94	2.5	-0.44	-0.02	-0.43
VOs	-639.46	5	-0.17	-0.39	-0.09
VIr	-637.81	2.5	-0.01	-0.75	0.29
VPt	-635.50	2	0.00	-0.99	0.49
VAu	-631.74	2.5	0.16	-0.19	0.24
VBi	-633.26	1.5	-0.34	-0.95	0.29
CrMn	-639.08	2	-1.05	-0.95	-0.57
CrFe	-638.53	2	-0.68	-1.03	-0.16
CrCo	-637.36	2	-0.60	-1.12	-0.04
CrNi	-636.26	2	-0.59	-1.24	0.03
CrCu	-634.48	2	-0.29	-1.18	0.31
CrZn	-631.59	2	-0.84	-1.16	-0.26
CrGa	-634.10	2.5	-0.73	-1.52	-0.12
CrY	-638.02	2.5	-1.64	-1.77	-0.93
CrZr	-639.00	3	-1.18	-1.22	-0.77
CrNb	-639.41	2.5	-1.01	-0.64	-0.75
CrMo	-639.08	2.5	-0.56	-0.11	-0.51
CrRu	-638.12	2	-0.23	-0.41	-0.02
CrRh	-636.98	2	-0.16	-0.81	0.25
CrPd	-635.12	2	0.02	-0.96	0.50
CrAg	-632.33	1.5	-0.05	-0.74	0.44

CrSn	-634.33	2	-0.53	-1.15	0.05
CrHf	-640.25	3	-1.23	-1.14	-0.85
CrTa	-640.69	2.5	-0.76	-0.41	-0.59
CrW	-640.53	2.5	-0.41	0.22	-0.49
CrRe	-640.09	2.5	-0.31	0.18	-0.38
CrOs	-639.28	5	-0.04	-0.02	-0.03
CrIr	-637.93	2.5	0.12	-0.53	0.34
CrPt	-636.07	2	0.13	-1.00	0.63
CrAu	-632.62	2.5	0.30	-0.36	0.44
CrBi	-633.49	1.5	-0.21	-0.79	0.32
MnFe	-637.84	2	-0.82	-0.92	-0.36
MnCo	-636.91	2	-0.74	-1.13	-0.17
MnNi	-636.17	2	-0.73	-1.43	-0.01
MnCu	-633.73	2	-0.42	-1.05	0.10
MnZn	-631.21	2	-0.98	-1.20	-0.37
MnGa	-633.35	2.5	-0.87	-1.39	-0.31
MnY	-637.85	2.5	-1.78	-1.92	-1.01
MnZr	-638.85	3	-1.32	-1.39	-0.86
MnNb	-639.10	2.5	-1.15	-0.73	-0.85
MnMo	-639.47	2.5	-0.70	-0.54	-0.48
MnRu	-637.80	2	-0.37	-0.49	-0.12
MnRh	-636.54	2	-0.30	-0.83	0.12
MnPd	-635.02	2	-0.12	-1.15	0.46
MnAg	-631.37	1.5	-0.19	-0.50	0.14
MnSn	-633.83	2	-0.67	-1.14	-0.09
MnHf	-640.18	3	-1.37	-1.35	-0.92
MnTa	-640.43	2.5	-0.90	-0.52	-0.69
MnW	-640.67	2.5	-0.54	-0.09	-0.51
MnRe	-639.65	2.5	-0.44	0.16	-0.51
MnOs	-639.15	5	-0.18	-0.20	-0.14
MnIr	-637.80	2.5	-0.02	-0.71	0.27
MnPt	-636.04	2	-0.01	-1.22	0.61
MnAu	-632.10	2.5	0.16	-0.33	0.29
MnBi	-632.98	1.5	-0.35	-0.77	0.17
FeCo	-636.09	2	-0.37	-1.07	0.17
FeNi	-634.75	2	-0.36	-1.07	0.18
FeCu	-632.59	2	-0.05	-0.83	0.36
FeZn	-629.95	2	-0.61	-0.92	-0.14
FeGa	-631.99	2.5	-0.50	-1.06	-0.08
FeY	-636.87	2.5	-1.41	-1.79	-0.70
FeZr	-637.89	3	-0.95	-1.26	-0.53
FeNb	-638.49	2.5	-0.78	-0.78	-0.46
FeMo	-638.38	2.5	-0.33	-0.35	-0.19
FeRu	-636.84	2	0.01	-0.36	0.19
FeRh	-635.64	2	0.07	-0.72	0.44
FePd	-633.65	2	0.25	-0.82	0.66
FeAg	-630.23	1.5	0.18	-0.29	0.37
FeSn	-632.68	2	-0.30	-0.92	0.16
FeHf	-639.25	3	-1.00	-1.23	-0.59
FeTa	-639.97	2.5	-0.53	-0.64	-0.27
FeW	-639.82	2.5	-0.18	-0.02	-0.17
FeRe	-639.21	2.5	-0.08	0.03	-0.09
FeOs	-637.91	5	0.19	0.07	0.18
FeIr	-636.77	2.5	0.36	-0.54	0.57
FePt	-634.66	2	0.37	-0.89	0.81
FeAu	-630.89	2.5	0.53	-0.08	0.56
FeBi	-632.01	1.5	0.02	-0.64	0.45
CoNi	-633.56	2	-0.27	-1.15	0.31
CoCu	-631.58	2	0.03	-1.00	0.53
CoZn	-628.96	2	-0.52	-1.10	0.03
CoGa	-630.83	2.5	-0.42	-1.16	0.05
CoY	-635.94	2.5	-1.33	-2.00	-0.53
CoZr	-637.09	3	-0.87	-1.53	-0.35
CoNb	-637.49	2.5	-0.69	-0.95	-0.31
CoMo	-637.21	2.5	-0.24	-0.44	-0.07
CoRu	-635.43	2	0.09	-0.34	0.26
CoRh	-634.30	2	0.16	-0.73	0.53
CoPd	-632.46	2	0.33	-0.90	0.78

CoAg	-629.26	1.5	0.26	-0.47	0.58
CoSn	-631.65	2	-0.21	-1.08	0.33
CoHf	-638.53	3	-0.92	-1.55	-0.40
CoTa	-638.91	2.5	-0.44	-0.79	-0.13
CoW	-638.75	2.5	-0.09	-0.16	-0.03
CoRe	-637.83	2.5	0.01	0.04	-0.01
CoOs	-636.64	5	0.28	0.03	0.27
CoIr	-635.40	2.5	0.44	-0.53	0.65
CoPt	-633.46	2	0.45	-0.96	0.93
CoAu	-629.91	2.5	0.61	-0.27	0.72
CoBi	-630.96	1.5	0.11	-0.79	0.64
NiCu	-630.42	2	0.04	-1.08	0.58
NiZn	-628.13	2	-0.51	-1.36	0.17
NiGa	-629.89	2.5	-0.41	-1.35	0.14
NiY	-634.86	2.5	-1.32	-2.12	-0.47
NiZr	-635.88	3	-0.86	-1.59	-0.32
NiNb	-636.09	2.5	-0.68	-0.92	-0.31
NiMo	-635.62	2.5	-0.23	-0.30	-0.11
NiRu	-634.08	2	0.10	-0.33	0.26
NiRh	-633.05	2	0.17	-0.77	0.56
NiPd	-631.53	2	0.35	-1.10	0.89
NiAg	-628.14	1.5	0.27	-0.58	0.66
NiSn	-630.62	2	-0.20	-1.23	0.41
NiHf	-637.27	3	-0.91	-1.58	-0.38
NiTa	-637.50	2.5	-0.43	-0.75	-0.13
NiW	-637.05	2.5	-0.08	0.03	-0.09
NiRe	-636.20	2.5	0.02	0.20	-0.06
NiOs	-635.31	5	0.29	0.03	0.28
NiIr	-634.18	2.5	0.45	-0.59	0.68
NiPt	-632.52	2	0.46	-1.15	1.04
NiAu	-628.92	2.5	0.62	-0.44	0.79
NiBi	-629.99	1.5	0.12	-0.97	0.77
CuZn	-626.44	2	-0.21	-1.35	0.47
CuGa	-627.84	2.5	-0.11	-1.16	0.36
CuY	-632.73	2.5	-1.02	-1.90	-0.26
CuZr	-633.32	3	-0.55	-1.15	-0.17
CuNb	-633.44	2.5	-0.38	-0.43	-0.21
CuMo	-633.32	2.5	0.07	0.01	0.07
CuRu	-631.98	2	0.40	-0.12	0.46
CuRh	-631.30	2	0.47	-0.73	0.84
CuPd	-629.48	2	0.65	-0.92	1.10
CuAg	-626.13	1.5	0.57	-0.42	0.85
CuSn	-628.80	2	0.10	-1.16	0.68
CuHf	-634.75	3	-0.61	-1.17	-0.22
CuTa	-634.58	2.5	-0.13	-0.13	-0.08
CuW	-634.50	2.5	0.22	0.47	0.03
CuRe	-633.78	2.5	0.32	0.56	0.09
CuOs	-633.11	5	0.59	0.29	0.53
CuIr	-632.32	2.5	0.75	-0.49	0.95
CuPt	-630.47	2	0.76	-0.97	1.24
CuAu	-626.34	2.5	0.92	0.02	0.91
CuBi	-627.34	1.5	0.42	-0.48	0.74
ZnGa	-625.95	2.5	-0.66	-1.64	0.00
ZnY	-630.71	2.5	-1.57	-2.30	-0.64
ZnZr	-631.29	3	-1.11	-1.55	-0.59
ZnNb	-631.44	2.5	-0.93	-0.84	-0.59
ZnMo	-631.35	2.5	-0.48	-0.42	-0.31
ZnRu	-629.93	2	-0.15	-0.50	0.10
ZnRh	-629.01	2	-0.08	-1.00	0.42
ZnPd	-626.88	2	0.09	-1.03	0.61
ZnAg	-624.32	1.5	0.02	-0.92	0.64
ZnSn	-626.10	2	-0.45	-1.23	0.16
ZnHf	-632.85	3	-1.16	-1.63	-0.61
ZnTa	-632.88	2.5	-0.68	-0.69	-0.40
ZnW	-632.71	2.5	-0.33	-0.06	-0.31
ZnRe	-631.87	2.5	-0.23	0.10	-0.27
ZnOs	-631.09	5	0.04	-0.12	0.06
ZnIr	-629.91	2.5	0.20	-0.71	0.48

ZnPt	-627.68	2	0.21	-0.99	0.71
ZnAu	-624.59	2.5	0.37	-0.53	0.58
ZnBi	-625.24	1.5	-0.13	-0.85	0.44
GaY	-632.66	3	-1.46	-2.39	-0.66
GaZr	-633.31	3.5	-1.00	-1.67	-0.52
GaNb	-633.66	3	-0.83	-1.07	-0.47
GaMo	-633.39	3	-0.38	-0.56	-0.19
GaRu	-631.73	2.5	-0.05	-0.52	0.16
GaRh	-630.91	2.5	0.02	-1.07	0.45
GaPd	-629.05	2.5	0.20	-1.23	0.69
GaAg	-626.04	2	0.13	-0.90	0.58
GaSn	-627.90	2.5	-0.35	-1.24	0.15
GaHf	-634.75	3.5	-1.05	-1.69	-0.57
GaTa	-634.91	3	-0.58	-0.82	-0.30
GaW	-634.71	3	-0.23	-0.17	-0.17
GaRe	-633.89	3	-0.13	-0.02	-0.12
GaOs	-632.97	5.5	0.14	-0.17	0.18
GaIr	-631.85	3	0.30	-0.79	0.57
GaPt	-629.98	2.5	0.31	-1.25	0.82
GaAu	-626.05	3	0.48	-0.37	0.60
GaBi	-626.72	2	-0.03	-0.71	0.33
YZr	-636.57	3.5	-1.91	-1.59	-1.46
YNb	-637.03	3	-1.74	-1.04	-1.39
YMo	-637.88	3	-1.29	-1.09	-0.92
YRu	-637.19	2.5	-0.96	-1.53	-0.34
YRh	-635.84	2.5	-0.89	-1.82	-0.16
YPd	-633.79	2.5	-0.71	-1.89	0.04
YAg	-630.37	2	-0.79	-1.35	-0.11
YSn	-632.83	2.5	-1.26	-1.99	-0.46
YHf	-638.76	3.5	-1.96	-1.98	-1.39
YTa	-639.12	3	-1.49	-1.21	-1.08
YW	-639.23	3	-1.14	-0.72	-0.90
YRe	-638.84	3	-1.04	-0.78	-0.77
YOs	-638.22	5.5	-0.77	-1.08	-0.57
YIr	-636.81	3	-0.61	-1.56	-0.09
YPt	-634.68	2.5	-0.60	-1.89	0.16
YAu	-631.00	3	-0.44	-1.13	-0.06
YBi	-632.58	2	-0.94	-1.92	0.03
ZrNb	-638.46	3.5	-1.28	-0.72	-1.07
ZrMo	-639.05	3.5	-0.83	-0.64	-0.64
ZrRu	-638.02	3	-0.50	-0.91	-0.19
ZrRh	-636.86	3	-0.43	-1.29	0.00
ZrPd	-634.68	3	-0.25	-1.29	0.18
ZrAg	-630.94	2.5	-0.32	-0.59	-0.09
ZrSn	-633.79	3	-0.79	-1.43	-0.32
ZrHf	-638.91	4	-1.50	-1.02	-1.25
ZrTa	-639.48	3.5	-1.03	-0.35	-0.93
ZrW	-640.36	3.5	-0.67	-0.24	-0.61
ZrRe	-639.89	3.5	-0.58	-0.27	-0.50
ZrOs	-639.12	6	-0.31	-0.49	-0.22
ZrIr	-638.00	3.5	-0.15	-1.11	0.17
ZrPt	-635.76	3	-0.14	-1.39	0.33
ZrAu	-631.65	3.5	0.03	-0.42	0.14
ZrBi	-633.48	2.5	-0.48	-1.33	0.06
NbMo	-639.40	3	-0.65	-0.03	-0.64
NbRu	-638.58	2.5	-0.32	-0.41	-0.16
NbRh	-637.14	2.5	-0.25	-0.65	0.01
NbPd	-634.81	2.5	-0.08	-0.58	0.16
NbAg	-632.43	2	-0.15	-0.56	0.13
NbSn	-634.09	2.5	-0.62	-0.80	-0.30
NbHf	-639.30	3.5	-1.33	-0.43	-1.20
NbTa	-640.36	3	-0.85	-0.01	-0.85
NbW	-640.83	3	-0.50	0.30	-0.60
NbRe	-640.32	3	-0.40	0.30	-0.50
NbOs	-639.96	5.5	-0.13	-0.13	-0.11
NbIr	-638.43	3	0.03	-0.55	0.21
NbPt	-635.92	2.5	0.04	-0.69	0.32
NbAu	-631.79	3	0.20	0.30	0.10

NbBi	-633.74	2	-0.30	-0.68	0.04
MoRu	-638.58	2.5	0.13	-0.03	0.14
MoRh	-636.87	2.5	0.20	-0.14	0.26
MoPd	-634.58	2.5	0.38	-0.09	0.41
MoAg	-630.92	2	0.30	0.57	0.02
MoSn	-634.06	2.5	-0.17	-0.41	-0.01
MoHf	-640.30	3.5	-0.88	-0.56	-0.71
MoTa	-640.77	3	-0.40	0.16	-0.45
MoW	-641.07	3	-0.05	0.56	-0.24
MoRe	-641.03	3	0.05	0.32	-0.06
MoOs	-639.96	5.5	0.32	0.24	0.28
MoIr	-638.15	3	0.48	-0.03	0.49
MoPt	-635.72	2.5	0.49	-0.22	0.58
MoAu	-631.53	3	0.65	0.80	0.38
MoBi	-633.59	2	0.15	-0.23	0.27
RuRh	-635.11	2	0.53	-0.05	0.56
RuPd	-632.99	2	0.71	-0.08	0.75
RuAg	-630.11	1.5	0.63	0.19	0.51
RuSn	-632.78	2	0.16	-0.56	0.44
RuHf	-639.45	3	-0.55	-0.93	-0.24
RuTa	-640.35	2.5	-0.07	-0.42	0.10
RuW	-640.19	2.5	0.28	0.21	0.20
RuRe	-639.25	2.5	0.38	0.42	0.21
RuOs	-637.97	5	0.65	0.45	0.56
RuIr	-636.16	2.5	0.81	0.17	0.74
RuPt	-634.08	2	0.82	-0.18	0.91
RuAu	-630.08	2.5	0.98	0.73	0.69
RuBi	-632.13	1.5	0.48	-0.29	0.67
RhPd	-631.88	2	0.77	-0.48	1.02
RhAg	-629.26	1.5	0.70	-0.35	0.93
RhSn	-631.79	2	0.23	-1.03	0.74
RhHf	-638.47	3	-0.48	-1.40	-0.01
RhTa	-638.72	2.5	0.00	-0.56	0.23
RhW	-638.44	2.5	0.35	0.12	0.30
RhRe	-637.46	2.5	0.45	0.36	0.31
RhOs	-636.35	5	0.72	0.30	0.66
RhIr	-635.16	2.5	0.88	-0.28	0.99
RhPt	-632.88	2	0.89	-0.55	1.16
RhAu	-629.28	2.5	1.05	0.18	0.98
RuBi	-630.69	1.5	0.55	-0.53	0.90
PdAg	-627.02	1.5	0.88	-0.32	1.09
PdSn	-629.55	2	0.40	-1.00	0.90
PdHf	-636.17	3	-0.30	-1.34	0.15
PdTa	-636.29	2.5	0.18	-0.44	0.35
PdW	-635.99	2.5	0.53	0.26	0.42
PdRe	-635.07	2.5	0.63	0.46	0.44
PdOs	-634.28	5	0.90	0.25	0.85
PdIr	-632.93	2.5	1.06	-0.26	1.16
PdPt	-631.14	2	1.07	-0.77	1.45
PdAu	-627.69	2.5	1.23	-0.12	1.27
PdBi	-629.07	1.5	0.73	-0.81	1.27
AgSn	-626.68	1.5	0.33	-0.74	0.82
AgHf	-632.38	2.5	-0.38	-0.61	-0.13
AgTa	-632.14	2	0.10	0.46	-0.13
AgW	-632.08	2	0.45	1.04	-0.07
AgRe	-632.25	2	0.55	0.70	0.20
AgOs	-631.28	4.5	0.82	0.57	0.69
AgIr	-630.23	2	0.98	-0.09	1.02
AgPt	-627.96	1.5	0.99	-0.35	1.22
AgAu	-624.21	2	1.15	0.45	0.93
AgBi	-625.15	1	0.65	-0.03	0.68
SnHf	-635.43	3	-0.85	-1.55	-0.33
SnTa	-635.54	2.5	-0.37	-0.65	-0.11
SnW	-635.40	2.5	-0.02	-0.03	-0.01
SnRe	-634.68	2.5	0.08	0.07	0.05
SnOs	-633.96	5	0.35	-0.18	0.39
SnIr	-632.78	2.5	0.51	-0.77	0.82
SnPt	-630.35	2	0.52	-0.95	1.00

SnAu	-627.11	2.5	0.68	-0.41	0.85
SnBi	-627.11	1.5	0.18	-0.42	0.46
HfTa	-640.67	3.5	-1.08	-0.25	-1.00
HfW	-641.59	3.5	-0.73	-0.16	-0.68
HfRe	-641.18	3.5	-0.63	-0.21	-0.57
HfOs	-640.42	6	-0.36	-0.44	-0.28
HfIr	-639.50	3.5	-0.20	-1.16	0.14
HfPt	-637.22	3	-0.19	-1.42	0.29
HfAu	-633.09	3.5	-0.03	-0.44	0.10
HfBi	-635.04	2.5	-0.53	-1.41	0.04
TaW	-642.12	3	-0.25	0.54	-0.43
TaRe	-641.71	3	-0.15	0.48	-0.31
TaOs	-641.51	5.5	0.12	-0.03	0.13
TaIr	-639.95	3	0.28	-0.43	0.42
TaPt	-637.43	2.5	0.29	-0.57	0.52
TaAu	-632.94	3	0.45	0.59	0.25
TaBi	-635.19	2	-0.05	-0.53	0.22
WRe	-642.44	3	0.20	0.67	-0.02
WOs	-641.56	5.5	0.47	0.49	0.38
WIr	-639.76	3	0.63	0.22	0.56
WPt	-637.16	2.5	0.64	0.12	0.59
WAu	-632.77	3	0.80	1.23	0.39
WBi	-635.03	2	0.30	0.10	0.25
ReOs	-640.65	5.5	0.57	0.69	0.44
ReIr	-638.72	3	0.73	0.48	0.57
RePt	-636.22	2.5	0.74	0.32	0.61
ReAu	-631.98	3	0.90	1.37	0.44
ReBi	-634.38	2	0.40	0.17	0.32
OsIr	-637.54	5.5	1.00	0.45	0.92
OsPt	-635.39	5	1.01	0.13	0.98
OsAu	-631.24	5.5	1.17	1.12	0.97
OsBi	-633.41	4.5	0.67	0.04	0.66
IrPt	-634.02	2.5	1.17	-0.36	1.32
IrAu	-630.36	3	1.33	0.38	1.20
IrBi	-631.69	2	0.83	-0.28	0.97
PtAu	-628.66	2.5	1.34	-0.16	1.41
PtBi	-630.02	1.5	0.84	-0.85	1.40
AuBi	-625.78	2	1.00	0.19	0.90
AlAl	-630.43	3	-1.66	-2.28	-0.90
ScSc	-636.02	3	-2.08	-2.48	-1.25
TiTi	-637.47	2	-1.63	-1.65	-0.81
VV	-638.34	2	-1.18	-0.90	-0.73
CrCr	-639.50	2	-0.91	-0.92	-0.45
MnMn	-638.71	2	-1.19	-1.01	-0.68
FeFe	-637.00	2	-0.45	-0.86	-0.02
CoCo	-634.71	2	-0.28	-1.06	0.25
NiNi	-632.63	2	-0.26	-1.35	0.41
CuCu	-628.32	2	0.34	-0.88	0.78
ZnZn	-623.81	2	-0.76	-1.45	-0.03
GaGa	-626.92	3	-0.55	-1.24	-0.14
YY	-635.75	3	-2.37	-2.22	-1.63
ZrZr	-637.64	4	-1.45	-1.08	-1.18
NbNb	-639.39	3	-1.10	-0.40	-0.97
MoMo	-640.90	3	-0.20	-0.40	-0.07
RuRu	-636.62	2	0.46	0.16	0.38
RhRh	-634.02	2	0.60	-0.47	0.83
PdPd	-630.20	2	0.95	-0.73	1.32
AgAg	-624.09	1	0.80	-0.03	0.83
SnSn	-628.05	2	-0.14	-0.83	0.27
HfHf	-640.17	4	-1.55	-0.95	-1.31
TaTa	-641.57	3	-0.60	0.26	-0.69
WW	-642.34	3	0.10	0.97	-0.22
ReRe	-641.82	3	0.30	0.72	0.06
OsOs	-639.40	8	0.84	0.70	0.75
IrIr	-636.38	3	1.16	-0.15	1.21
PtPt	-632.11	2	1.18	-0.81	1.59
AuAu	-624.28	3	1.50	0.94	1.19
BiBi	-626.18	1	0.50	0.00	0.50

Table S3. Parameter values for machine learning analysis of catalyst stability: AtomNumber (Z), valence-electron number (VP)r, electronegativity (χ), electron affinity (EA), atomic radius (r), First ionization energy (IE), s electron number (Ns)d electron number (Nd), p electron number (Np) of dual-atom.

Metal	Z1	Z2	VP1	VP2	χ1	χ2	EA1	EA2	r1	r2	IE1	IE2	Ns1	Ns2	Nd1	Nd2	Np1	Np2
AlSc	13	21	3	3	1.61	1.36	0.44	0.19	118	144	5.99	6.54	1	2	0	1	2	6
AlTi	13	22	3	4	1.61	1.54	0.44	0.08	118	132	5.99	6.82	1	2	0	2	2	0
AlV	13	23	3	5	1.61	1.63	0.44	0.53	118	122	5.99	6.74	1	1	0	4	2	6
AlCr	13	24	3	6	1.61	1.66	0.44	0.68	118	118	5.99	6.77	1	1	0	5	2	0
AlMn	13	25	3	7	1.61	1.55	0.44	-0.50	118	117	5.99	7.44	1	1	0	6	2	0
AlFe	13	26	3	8	1.61	1.83	0.44	0.15	118	117	5.99	7.87	1	1	0	7	2	0
AlCo	13	27	3	9	1.61	1.88	0.44	0.66	118	116	5.99	7.86	1	1	0	8	2	0
AlNi	13	28	3	10	1.61	1.91	0.44	1.16	118	115	5.99	7.64	1	2	0	8	2	0
AlCu	13	29	3	11	1.61	1.9	0.44	1.24	118	117	5.99	7.73	1	1	0	10	2	0
AlZn	13	30	3	12	1.61	1.65	0.44	0.09	118	125	5.99	9.40	1	2	0	10	2	0
AlGa	13	31	3	3	1.61	1.81	0.44	0.30	118	126	5.99	6.00	1	2	0	0	2	1
AlY	13	39	3	11	1.61	1.22	0.44	0.75	118	162	5.99	6.39	1	4	0	1	2	6
AlZr	13	40	3	4	1.61	1.33	0.44	0.43	118	145	5.99	6.84	1	4	0	2	2	6
AlNb	13	41	3	11	1.61	1.6	0.44	0.92	118	134	5.99	6.89	1	1	0	4	2	6
AlMo	13	42	3	6	1.61	2.16	0.44	0.75	118	130	5.99	7.10	1	1	0	5	2	6
AlRu	13	44	3	8	1.61	2.2	0.44	1.05	118	125	5.99	7.37	1	1	0	7	2	0
AlRh	13	45	3	9	1.61	2.28	0.44	1.14	118	125	5.99	7.47	1	1	0	8	2	0
AlPd	13	46	3	10	1.61	2.2	0.44	0.56	118	128	5.99	8.35	1	1	0	9	2	0
AlAg	13	47	3	11	1.61	1.93	0.44	1.30	118	134	5.99	7.58	1	1	0	10	2	0
AlSn	13	50	3	4	1.61	1.96	0.44	1.20	118	140	5.99	7.35	1	2	0	0	2	2
AlHf	13	72	3	4	1.61	1.3	0.44	0.18	118	144	5.99	7.00	1	2	0	2	2	0
AlTa	13	73	3	11	1.61	1.5	0.44	0.32	118	134	5.99	7.89	1	2	0	3	2	0
AlW	13	74	3	6	1.61	2.38	0.44	0.82	118	130	5.99	7.98	1	2	0	4	2	0
AlRe	13	75	3	7	1.61	1.9	0.44	0.06	118	128	5.99	7.88	1	2	0	5	2	6
AlOs	13	76	3	8	1.61	2.2	0.44	1.08	118	126	5.99	8.71	1	2	0	6	2	0
AlIr	13	77	3	9	1.61	2.2	0.44	1.57	118	127	5.99	9.12	1	1	0	8	2	0
AlPt	13	78	3	10	1.61	2.28	0.44	2.13	118	130	5.99	9.02	1	1	0	9	2	0
AlAu	13	79	3	11	1.61	2.54	0.44	2.31	118	134	5.99	9.23	1	1	0	10	2	0
AlBi	13	83	3	5	1.61	2.02	0.44	0.95	118	146	5.99	7.29	1	2	0	0	2	3
ScTi	21	22	3	4	1.36	1.54	0.19	0.08	144	132	6.54	6.82	2	2	1	2	6	0
ScV	21	23	3	5	1.36	1.63	0.19	0.53	144	122	6.54	6.74	2	1	1	4	6	6
ScCr	21	24	3	6	1.36	1.66	0.19	0.68	144	118	6.54	6.77	2	1	1	5	6	0
ScMn	21	25	3	7	1.36	1.55	0.19	-0.50	144	117	6.54	7.44	2	1	1	6	6	0
ScFe	21	26	3	8	1.36	1.83	0.19	0.15	144	117	6.54	7.87	2	1	1	7	6	0
ScCo	21	27	3	9	1.36	1.88	0.19	0.66	144	116	6.54	7.86	2	1	1	8	6	0
ScNi	21	28	3	10	1.36	1.91	0.19	1.16	144	115	6.54	7.64	2	2	1	8	6	0
ScCu	21	29	3	11	1.36	1.9	0.19	1.24	144	117	6.54	7.73	2	1	1	10	6	0
ScZn	21	30	3	12	1.36	1.65	0.19	0.09	144	125	6.54	9.40	2	2	1	10	6	0
ScGa	21	31	3	3	1.36	1.81	0.19	0.30	144	126	6.54	6.00	2	2	1	0	6	1

ScY	21	39	3	11	1.36	1.22	0.19	0.75	144	162	6.54	6.39	2	4	1	1	6	6
ScZr	21	40	3	4	1.36	1.33	0.19	0.43	144	145	6.54	6.84	2	4	1	2	6	6
ScNb	21	41	3	11	1.36	1.6	0.19	0.92	144	134	6.54	6.89	2	1	1	4	6	6
ScMo	21	42	3	6	1.36	2.16	0.19	0.75	144	130	6.54	7.10	2	1	1	5	6	6
ScRu	21	44	3	8	1.36	2.2	0.19	1.05	144	125	6.54	7.37	2	1	1	7	6	0
ScRh	21	45	3	9	1.36	2.28	0.19	1.14	144	125	6.54	7.47	2	1	1	8	6	0
ScPd	21	46	3	10	1.36	2.2	0.19	0.56	144	128	6.54	8.35	2	1	1	9	6	0
ScAg	21	47	3	11	1.36	1.93	0.19	1.30	144	134	6.54	7.58	2	1	1	10	6	0
ScSn	21	50	3	4	1.36	1.96	0.19	1.20	144	140	6.54	7.35	2	2	1	0	6	2
ScHf	21	72	3	4	1.36	1.3	0.19	0.18	144	144	6.54	7.00	2	2	1	2	6	0
ScTa	21	73	3	11	1.36	1.5	0.19	0.32	144	134	6.54	7.89	2	2	1	3	6	0
ScW	21	74	3	6	1.36	2.38	0.19	0.82	144	130	6.54	7.98	2	2	1	4	6	0
ScRe	21	75	3	7	1.36	1.9	0.19	0.06	144	128	6.54	7.88	2	2	1	5	6	6
ScOs	21	76	3	8	1.36	2.2	0.19	1.08	144	126	6.54	8.71	2	2	1	6	6	0
ScIr	21	77	3	9	1.36	2.2	0.19	1.57	144	127	6.54	9.12	2	1	1	8	6	0
ScPt	21	78	3	10	1.36	2.28	0.19	2.13	144	130	6.54	9.02	2	1	1	9	6	0
ScAu	21	79	3	11	1.36	2.54	0.19	2.31	144	134	6.54	9.23	2	1	1	10	6	0
ScBi	21	83	3	5	1.36	2.02	0.19	0.95	144	146	6.54	7.29	2	2	1	0	6	3
TiV	22	23	4	5	1.54	1.63	0.08	0.53	132	122	6.82	6.74	2	1	2	4	0	6
TiCr	22	24	4	6	1.54	1.66	0.08	0.68	132	118	6.82	6.77	2	1	2	5	0	0
TiMn	22	25	4	7	1.54	1.55	0.08	-0.50	132	117	6.82	7.44	2	1	2	6	0	0
TiFe	22	26	4	8	1.54	1.83	0.08	0.15	132	117	6.82	7.87	2	1	2	7	0	0
TiCo	22	27	4	9	1.54	1.88	0.08	0.66	132	116	6.82	7.86	2	1	2	8	0	0
TiNi	22	28	4	10	1.54	1.91	0.08	1.16	132	115	6.82	7.64	2	2	2	8	0	0
TiCu	22	29	4	11	1.54	1.9	0.08	1.24	132	117	6.82	7.73	2	1	2	10	0	0
TiZn	22	30	4	12	1.54	1.65	0.08	0.09	132	125	6.82	9.40	2	2	2	10	0	0
TiGa	22	31	4	3	1.54	1.81	0.08	0.30	132	126	6.82	6.00	2	2	2	0	0	1
TiY	22	39	4	11	1.54	1.22	0.08	0.75	132	162	6.82	6.39	2	4	2	1	0	6
TiZr	22	40	4	4	1.54	1.33	0.08	0.43	132	145	6.82	6.84	2	4	2	2	0	6
TiNb	22	41	4	11	1.54	1.6	0.08	0.92	132	134	6.82	6.89	2	1	2	4	0	6
TiMo	22	42	4	6	1.54	2.16	0.08	0.75	132	130	6.82	7.10	2	1	2	5	0	6
TiRu	22	44	4	8	1.54	2.2	0.08	1.05	132	125	6.82	7.37	2	1	2	7	0	0
TiRh	22	45	4	9	1.54	2.28	0.08	1.14	132	125	6.82	7.47	2	1	2	8	0	0
TiPd	22	46	4	10	1.54	2.2	0.08	0.56	132	128	6.82	8.35	2	1	2	9	0	0
TiAg	22	47	4	11	1.54	1.93	0.08	1.30	132	134	6.82	7.58	2	1	2	10	0	0
TiSn	22	50	4	4	1.54	1.96	0.08	1.20	132	140	6.82	7.35	2	2	2	0	0	2
TiHf	22	72	4	4	1.54	1.3	0.08	0.18	132	144	6.82	7.00	2	2	2	2	0	0
TiTa	22	73	4	11	1.54	1.5	0.08	0.32	132	134	6.82	7.89	2	2	2	3	0	0
TiW	22	74	4	6	1.54	2.38	0.08	0.82	132	130	6.82	7.98	2	2	2	4	0	0
TiRe	22	75	4	7	1.54	1.9	0.08	0.06	132	128	6.82	7.88	2	2	2	5	0	6
TiOs	22	76	4	8	1.54	2.2	0.08	1.08	132	126	6.82	8.71	2	2	2	6	0	0
TiIr	22	77	4	9	1.54	2.2	0.08	1.57	132	127	6.82	9.12	2	1	2	8	0	0
TiPt	22	78	4	10	1.54	2.28	0.08	2.13	132	130	6.82	9.02	2	1	2	9	0	0
TiAu	22	79	4	11	1.54	2.54	0.08	2.31	132	134	6.82	9.23	2	1	2	10	0	0
TiBi	22	83	4	5	1.54	2.02	0.08	0.95	132	146	6.82	7.29	2	2	2	0	0	3
VCr	23	24	5	6	1.63	1.66	0.53	0.68	122	118	6.74	6.77	1	1	4	5	6	0
VMn	23	25	5	7	1.63	1.55	0.53	-0.50	122	117	6.74	7.44	1	1	4	6	6	0

VFe	23	26	5	8	1.63	1.83	0.53	0.15	122	117	6.74	7.87	1	1	4	7	6	0
VCo	23	27	5	9	1.63	1.88	0.53	0.66	122	116	6.74	7.86	1	1	4	8	6	0
VNi	23	28	5	10	1.63	1.91	0.53	1.16	122	115	6.74	7.64	1	2	4	8	6	0
VCu	23	29	5	11	1.63	1.9	0.53	1.24	122	117	6.74	7.73	1	1	4	10	6	0
VZn	23	30	5	12	1.63	1.65	0.53	0.09	122	125	6.74	9.40	1	2	4	10	6	0
VGa	23	31	5	3	1.63	1.81	0.53	0.30	122	126	6.74	6.00	1	2	4	0	6	1
VY	23	39	5	11	1.63	1.22	0.53	0.75	122	162	6.74	6.39	1	4	4	1	6	6
VZr	23	40	5	4	1.63	1.33	0.53	0.43	122	145	6.74	6.84	1	4	4	2	6	6
VNb	23	41	5	11	1.63	1.6	0.53	0.92	122	134	6.74	6.89	1	1	4	4	6	6
VMo	23	42	5	6	1.63	2.16	0.53	0.75	122	130	6.74	7.10	1	1	4	5	6	6
VRu	23	44	5	8	1.63	2.2	0.53	1.05	122	125	6.74	7.37	1	1	4	7	6	0
VRh	23	45	5	9	1.63	2.28	0.53	1.14	122	125	6.74	7.47	1	1	4	8	6	0
VPd	23	46	5	10	1.63	2.2	0.53	0.56	122	128	6.74	8.35	1	1	4	9	6	0
VAg	23	47	5	11	1.63	1.93	0.53	1.30	122	134	6.74	7.58	1	1	4	10	6	0
VSn	23	50	5	4	1.63	1.96	0.53	1.20	122	140	6.74	7.35	1	2	4	0	6	2
VHf	23	72	5	4	1.63	1.3	0.53	0.18	122	144	6.74	7.00	1	2	4	2	6	0
VTa	23	73	5	11	1.63	1.5	0.53	0.32	122	134	6.74	7.89	1	2	4	3	6	0
VW	23	74	5	6	1.63	2.38	0.53	0.82	122	130	6.74	7.98	1	2	4	4	6	0
VRe	23	75	5	7	1.63	1.9	0.53	0.06	122	128	6.74	7.88	1	2	4	5	6	6
VOs	23	76	5	8	1.63	2.2	0.53	1.08	122	126	6.74	8.71	1	2	4	6	6	0
VIr	23	77	5	9	1.63	2.2	0.53	1.57	122	127	6.74	9.12	1	1	4	8	6	0
VPt	23	78	5	10	1.63	2.28	0.53	2.13	122	130	6.74	9.02	1	1	4	9	6	0
VAu	23	79	5	11	1.63	2.54	0.53	2.31	122	134	6.74	9.23	1	1	4	10	6	0
VBi	23	83	5	5	1.63	2.02	0.53	0.95	122	146	6.74	7.29	1	2	4	0	6	3
CrMn	24	25	6	7	1.66	1.55	0.68	- 0.50	118	117	6.77	7.44	1	1	5	6	0	0
CrFe	24	26	6	8	1.66	1.83	0.68	0.15	118	117	6.77	7.87	1	1	5	7	0	0
CrCo	24	27	6	9	1.66	1.88	0.68	0.66	118	116	6.77	7.86	1	1	5	8	0	0
CrNi	24	28	6	10	1.66	1.91	0.68	1.16	118	115	6.77	7.64	1	2	5	8	0	0
CrCu	24	29	6	11	1.66	1.9	0.68	1.24	118	117	6.77	7.73	1	1	5	10	0	0
CrZn	24	30	6	12	1.66	1.65	0.68	0.09	118	125	6.77	9.40	1	2	5	10	0	0
CrGa	24	31	6	3	1.66	1.81	0.68	0.30	118	126	6.77	6.00	1	2	5	0	0	1
CrY	24	39	6	11	1.66	1.22	0.68	0.75	118	162	6.77	6.39	1	4	5	1	0	6
CrZr	24	40	6	4	1.66	1.33	0.68	0.43	118	145	6.77	6.84	1	4	5	2	0	6
CrNb	24	41	6	11	1.66	1.6	0.68	0.92	118	134	6.77	6.89	1	1	5	4	0	6
CrMo	24	42	6	6	1.66	2.16	0.68	0.75	118	130	6.77	7.10	1	1	5	5	0	6
CrRu	24	44	6	8	1.66	2.2	0.68	1.05	118	125	6.77	7.37	1	1	5	7	0	0
CrRh	24	45	6	9	1.66	2.28	0.68	1.14	118	125	6.77	7.47	1	1	5	8	0	0
CrPd	24	46	6	10	1.66	2.2	0.68	0.56	118	128	6.77	8.35	1	1	5	9	0	0
CrAg	24	47	6	11	1.66	1.93	0.68	1.30	118	134	6.77	7.58	1	1	5	10	0	0
CrSn	24	50	6	4	1.66	1.96	0.68	1.20	118	140	6.77	7.35	1	2	5	0	0	2
CrHf	24	72	6	4	1.66	1.3	0.68	0.18	118	144	6.77	7.00	1	2	5	2	0	0
CrTa	24	73	6	11	1.66	1.5	0.68	0.32	118	134	6.77	7.89	1	2	5	3	0	0
CrW	24	74	6	6	1.66	2.38	0.68	0.82	118	130	6.77	7.98	1	2	5	4	0	0
CrRe	24	75	6	7	1.66	1.9	0.68	0.06	118	128	6.77	7.88	1	2	5	5	0	6
CrOs	24	76	6	8	1.66	2.2	0.68	1.08	118	126	6.77	8.71	1	2	5	6	0	0
CrIr	24	77	6	9	1.66	2.2	0.68	1.57	118	127	6.77	9.12	1	1	5	8	0	0
CrPt	24	78	6	10	1.66	2.28	0.68	2.13	118	130	6.77	9.02	1	1	5	9	0	0

CrAu	24	79	6	11	1.66	2.54	0.68	2.31	118	134	6.77	9.23	1	1	5	10	0	0
CrBi	24	83	6	5	1.66	2.02	0.68	0.95	118	146	6.77	7.29	1	2	5	0	0	3
MnFe	25	26	7	8	1.55	1.83	-0.50	0.15	117	117	7.44	7.87	1	1	6	7	0	0
MnCo	25	27	7	9	1.55	1.88	-0.50	0.66	117	116	7.44	7.86	1	1	6	8	0	0
MnNi	25	28	7	10	1.55	1.91	-0.50	1.16	117	115	7.44	7.64	1	2	6	8	0	0
MnCu	25	29	7	11	1.55	1.9	-0.50	1.24	117	117	7.44	7.73	1	1	6	10	0	0
MnZn	25	30	7	12	1.55	1.65	-0.50	0.09	117	125	7.44	9.40	1	2	6	10	0	0
MnGa	25	31	7	3	1.55	1.81	-0.50	0.30	117	126	7.44	6.00	1	2	6	0	0	1
MnY	25	39	7	11	1.55	1.22	-0.50	0.75	117	162	7.44	6.39	1	4	6	1	0	6
MnZr	25	40	7	4	1.55	1.33	-0.50	0.43	117	145	7.44	6.84	1	4	6	2	0	6
MnNb	25	41	7	11	1.55	1.6	-0.50	0.92	117	134	7.44	6.89	1	1	6	4	0	6
MnMo	25	42	7	6	1.55	2.16	-0.50	0.75	117	130	7.44	7.10	1	1	6	5	0	6
MnRu	25	44	7	8	1.55	2.2	-0.50	1.05	117	125	7.44	7.37	1	1	6	7	0	0
MnRh	25	45	7	9	1.55	2.28	-0.50	1.14	117	125	7.44	7.47	1	1	6	8	0	0
MnPd	25	46	7	10	1.55	2.2	-0.50	0.56	117	128	7.44	8.35	1	1	6	9	0	0
MnAg	25	47	7	11	1.55	1.93	-0.50	1.30	117	134	7.44	7.58	1	1	6	10	0	0
MnSn	25	50	7	4	1.55	1.96	-0.50	1.20	117	140	7.44	7.35	1	2	6	0	0	2
MnHf	25	72	7	4	1.55	1.3	-0.50	0.18	117	144	7.44	7.00	1	2	6	2	0	0
MnTa	25	73	7	11	1.55	1.5	-0.50	0.32	117	134	7.44	7.89	1	2	6	3	0	0
MnW	25	74	7	6	1.55	2.38	-0.50	0.82	117	130	7.44	7.98	1	2	6	4	0	0
MnRe	25	75	7	7	1.55	1.9	-0.50	0.06	117	128	7.44	7.88	1	2	6	5	0	6
MnOs	25	76	7	8	1.55	2.2	-0.50	1.08	117	126	7.44	8.71	1	2	6	6	0	0
MnIr	25	77	7	9	1.55	2.2	-0.50	1.57	117	127	7.44	9.12	1	1	6	8	0	0
MnPt	25	78	7	10	1.55	2.28	-0.50	2.13	117	130	7.44	9.02	1	1	6	9	0	0
MnAu	25	79	7	11	1.55	2.54	-0.50	2.31	117	134	7.44	9.23	1	1	6	10	0	0
MnBi	25	83	7	5	1.55	2.02	-0.50	0.95	117	146	7.44	7.29	1	2	6	0	0	3
FeCo	26	27	8	9	1.83	1.88	0.15	0.66	117	116	7.87	7.86	1	1	7	8	0	0
FeNi	26	28	8	10	1.83	1.91	0.15	1.16	117	115	7.87	7.64	1	2	7	8	0	0
FeCu	26	29	8	11	1.83	1.9	0.15	1.24	117	117	7.87	7.73	1	1	7	10	0	0
FeZn	26	30	8	12	1.83	1.65	0.15	0.09	117	125	7.87	9.40	1	2	7	10	0	0
FeGa	26	31	8	3	1.83	1.81	0.15	0.30	117	126	7.87	6.00	1	2	7	0	0	1
FeY	26	39	8	11	1.83	1.22	0.15	0.75	117	162	7.87	6.39	1	4	7	1	0	6
FeZr	26	40	8	4	1.83	1.33	0.15	0.43	117	145	7.87	6.84	1	4	7	2	0	6
FeNb	26	41	8	11	1.83	1.6	0.15	0.92	117	134	7.87	6.89	1	1	7	4	0	6
FeMo	26	42	8	6	1.83	2.16	0.15	0.75	117	130	7.87	7.10	1	1	7	5	0	6
FeRu	26	44	8	8	1.83	2.2	0.15	1.05	117	125	7.87	7.37	1	1	7	7	0	0
FeRh	26	45	8	9	1.83	2.28	0.15	1.14	117	125	7.87	7.47	1	1	7	8	0	0
FePd	26	46	8	10	1.83	2.2	0.15	0.56	117	128	7.87	8.35	1	1	7	9	0	0
FeAg	26	47	8	11	1.83	1.93	0.15	1.30	117	134	7.87	7.58	1	1	7	10	0	0
FeSn	26	50	8	4	1.83	1.96	0.15	1.20	117	140	7.87	7.35	1	2	7	0	0	2
FeHf	26	72	8	4	1.83	1.3	0.15	0.18	117	144	7.87	7.00	1	2	7	2	0	0
FeTa	26	73	8	11	1.83	1.5	0.15	0.32	117	134	7.87	7.89	1	2	7	3	0	0
FeW	26	74	8	6	1.83	2.38	0.15	0.82	117	130	7.87	7.98	1	2	7	4	0	0
FeRe	26	75	8	7	1.83	1.9	0.15	0.06	117	128	7.87	7.88	1	2	7	5	0	6
FeOs	26	76	8	8	1.83	2.2	0.15	1.08	117	126	7.87	8.71	1	2	7	6	0	0
FeIr	26	77	8	9	1.83	2.2	0.15	1.57	117	127	7.87	9.12	1	1	7	8	0	0
FePt	26	78	8	10	1.83	2.28	0.15	2.13	117	130	7.87	9.02	1	1	7	9	0	0

FeAu	26	79	8	11	1.83	2.54	0.15	2.31	117	134	7.87	9.23	1	1	7	10	0	0
FeBi	26	83	8	5	1.83	2.02	0.15	0.95	117	146	7.87	7.29	1	2	7	0	0	3
CoNi	27	28	9	10	1.88	1.91	0.66	1.16	116	115	7.86	7.64	1	2	8	8	0	0
CoCu	27	29	9	11	1.88	1.9	0.66	1.24	116	117	7.86	7.73	1	1	8	10	0	0
CoZn	27	30	9	12	1.88	1.65	0.66	0.09	116	125	7.86	9.40	1	2	8	10	0	0
CoGa	27	31	9	3	1.88	1.81	0.66	0.30	116	126	7.86	6.00	1	2	8	0	0	1
CoY	27	39	9	11	1.88	1.22	0.66	0.75	116	162	7.86	6.39	1	4	8	1	0	6
CoZr	27	40	9	4	1.88	1.33	0.66	0.43	116	145	7.86	6.84	1	4	8	2	0	6
CoNb	27	41	9	11	1.88	1.6	0.66	0.92	116	134	7.86	6.89	1	1	8	4	0	6
CoMo	27	42	9	6	1.88	2.16	0.66	0.75	116	130	7.86	7.10	1	1	8	5	0	6
CoRu	27	44	9	8	1.88	2.2	0.66	1.05	116	125	7.86	7.37	1	1	8	7	0	0
CoRh	27	45	9	9	1.88	2.28	0.66	1.14	116	125	7.86	7.47	1	1	8	8	0	0
CoPd	27	46	9	10	1.88	2.2	0.66	0.56	116	128	7.86	8.35	1	1	8	9	0	0
CoAg	27	47	9	11	1.88	1.93	0.66	1.30	116	134	7.86	7.58	1	1	8	10	0	0
CoSn	27	50	9	4	1.88	1.96	0.66	1.20	116	140	7.86	7.35	1	2	8	0	0	2
CoHf	27	72	9	4	1.88	1.3	0.66	0.18	116	144	7.86	7.00	1	2	8	2	0	0
CoTa	27	73	9	11	1.88	1.5	0.66	0.32	116	134	7.86	7.89	1	2	8	3	0	0
CoW	27	74	9	6	1.88	2.38	0.66	0.82	116	130	7.86	7.98	1	2	8	4	0	0
CoRe	27	75	9	7	1.88	1.9	0.66	0.06	116	128	7.86	7.88	1	2	8	5	0	6
CoOs	27	76	9	8	1.88	2.2	0.66	1.08	116	126	7.86	8.71	1	2	8	6	0	0
CoIr	27	77	9	9	1.88	2.2	0.66	1.57	116	127	7.86	9.12	1	1	8	8	0	0
CoPt	27	78	9	10	1.88	2.28	0.66	2.13	116	130	7.86	9.02	1	1	8	9	0	0
CoAu	27	79	9	11	1.88	2.54	0.66	2.31	116	134	7.86	9.23	1	1	8	10	0	0
CoBi	27	83	9	5	1.88	2.02	0.66	0.95	116	146	7.86	7.29	1	2	8	0	0	3
NiCu	28	29	10	11	1.91	1.9	1.16	1.24	115	117	7.64	7.73	2	1	8	10	0	0
NiZn	28	30	10	12	1.91	1.65	1.16	0.09	115	125	7.64	9.40	2	2	8	10	0	0
NiGa	28	31	10	3	1.91	1.81	1.16	0.30	115	126	7.64	6.00	2	2	8	0	0	1
NiY	28	39	10	11	1.91	1.22	1.16	0.75	115	162	7.64	6.39	2	4	8	1	0	6
NiZr	28	40	10	4	1.91	1.33	1.16	0.43	115	145	7.64	6.84	2	4	8	2	0	6
NiNb	28	41	10	11	1.91	1.6	1.16	0.92	115	134	7.64	6.89	2	1	8	4	0	6
NiMo	28	42	10	6	1.91	2.16	1.16	0.75	115	130	7.64	7.10	2	1	8	5	0	6
NiRu	28	44	10	8	1.91	2.2	1.16	1.05	115	125	7.64	7.37	2	1	8	7	0	0
NiRh	28	45	10	9	1.91	2.28	1.16	1.14	115	125	7.64	7.47	2	1	8	8	0	0
NiPd	28	46	10	10	1.91	2.2	1.16	0.56	115	128	7.64	8.35	2	1	8	9	0	0
NiAg	28	47	10	11	1.91	1.93	1.16	1.30	115	134	7.64	7.58	2	1	8	10	0	0
NiSn	28	50	10	4	1.91	1.96	1.16	1.20	115	140	7.64	7.35	2	2	8	0	0	2
NiHf	28	72	10	4	1.91	1.3	1.16	0.18	115	144	7.64	7.00	2	2	8	2	0	0
NiTa	28	73	10	11	1.91	1.5	1.16	0.32	115	134	7.64	7.89	2	2	8	3	0	0
NiW	28	74	10	6	1.91	2.38	1.16	0.82	115	130	7.64	7.98	2	2	8	4	0	0
NiRe	28	75	10	7	1.91	1.9	1.16	0.06	115	128	7.64	7.88	2	2	8	5	0	6
NiOs	28	76	10	8	1.91	2.2	1.16	1.08	115	126	7.64	8.71	2	2	8	6	0	0
NiIr	28	77	10	9	1.91	2.2	1.16	1.57	115	127	7.64	9.12	2	1	8	8	0	0
NiPt	28	78	10	10	1.91	2.28	1.16	2.13	115	130	7.64	9.02	2	1	8	9	0	0
NiAu	28	79	10	11	1.91	2.54	1.16	2.31	115	134	7.64	9.23	2	1	8	10	0	0
NiBi	28	83	10	5	1.91	2.02	1.16	0.95	115	146	7.64	7.29	2	2	8	0	0	3
CuZn	29	30	11	12	1.9	1.65	1.24	0.09	117	125	7.73	9.40	1	2	10	10	0	0
CuGa	29	31	11	3	1.9	1.81	1.24	0.30	117	126	7.73	6.00	1	2	10	0	0	1

CuY	29	39	11	11	1.9	1.22	1.24	0.75	117	162	7.73	6.39	1	4	10	1	0	6
CuZr	29	40	11	4	1.9	1.33	1.24	0.43	117	145	7.73	6.84	1	4	10	2	0	6
CuNb	29	41	11	11	1.9	1.6	1.24	0.92	117	134	7.73	6.89	1	1	10	4	0	6
CuMo	29	42	11	6	1.9	2.16	1.24	0.75	117	130	7.73	7.10	1	1	10	5	0	6
CuRu	29	44	11	8	1.9	2.2	1.24	1.05	117	125	7.73	7.37	1	1	10	7	0	0
CuRh	29	45	11	9	1.9	2.28	1.24	1.14	117	125	7.73	7.47	1	1	10	8	0	0
CuPd	29	46	11	10	1.9	2.2	1.24	0.56	117	128	7.73	8.35	1	1	10	9	0	0
CuAg	29	47	11	11	1.9	1.93	1.24	1.30	117	134	7.73	7.58	1	1	10	10	0	0
CuSn	29	50	11	4	1.9	1.96	1.24	1.20	117	140	7.73	7.35	1	2	10	0	0	2
CuHf	29	72	11	4	1.9	1.3	1.24	0.18	117	144	7.73	7.00	1	2	10	2	0	0
CuTa	29	73	11	11	1.9	1.5	1.24	0.32	117	134	7.73	7.89	1	2	10	3	0	0
CuW	29	74	11	6	1.9	2.38	1.24	0.82	117	130	7.73	7.98	1	2	10	4	0	0
CuRe	29	75	11	7	1.9	1.9	1.24	0.06	117	128	7.73	7.88	1	2	10	5	0	6
CuOs	29	76	11	8	1.9	2.2	1.24	1.08	117	126	7.73	8.71	1	2	10	6	0	0
CuIr	29	77	11	9	1.9	2.2	1.24	1.57	117	127	7.73	9.12	1	1	10	8	0	0
CuPt	29	78	11	10	1.9	2.28	1.24	2.13	117	130	7.73	9.02	1	1	10	9	0	0
CuAu	29	79	11	11	1.9	2.54	1.24	2.31	117	134	7.73	9.23	1	1	10	10	0	0
CuBi	29	83	11	5	1.9	2.02	1.24	0.95	117	146	7.73	7.29	1	2	10	0	0	3
ZnGa	30	31	12	3	1.65	1.81	0.09	0.30	125	126	9.40	6.00	2	2	10	0	0	1
ZnY	30	39	12	11	1.65	1.22	0.09	0.75	125	162	9.40	6.39	2	4	10	1	0	6
ZnZr	30	40	12	4	1.65	1.33	0.09	0.43	125	145	9.40	6.84	2	4	10	2	0	6
ZnNb	30	41	12	11	1.65	1.6	0.09	0.92	125	134	9.40	6.89	2	1	10	4	0	6
ZnMo	30	42	12	6	1.65	2.16	0.09	0.75	125	130	9.40	7.10	2	1	10	5	0	6
ZnRu	30	44	12	8	1.65	2.2	0.09	1.05	125	125	9.40	7.37	2	1	10	7	0	0
ZnRh	30	45	12	9	1.65	2.28	0.09	1.14	125	125	9.40	7.47	2	1	10	8	0	0
ZnPd	30	46	12	10	1.65	2.2	0.09	0.56	125	128	9.40	8.35	2	1	10	9	0	0
ZnAg	30	47	12	11	1.65	1.93	0.09	1.30	125	134	9.40	7.58	2	1	10	10	0	0
ZnSn	30	50	12	4	1.65	1.96	0.09	1.20	125	140	9.40	7.35	2	2	10	0	0	2
ZnHf	30	72	12	4	1.65	1.3	0.09	0.18	125	144	9.40	7.00	2	2	10	2	0	0
ZnTa	30	73	12	11	1.65	1.5	0.09	0.32	125	134	9.40	7.89	2	2	10	3	0	0
ZnW	30	74	12	6	1.65	2.38	0.09	0.82	125	130	9.40	7.98	2	2	10	4	0	0
ZnRe	30	75	12	7	1.65	1.9	0.09	0.06	125	128	9.40	7.88	2	2	10	5	0	6
ZnOs	30	76	12	8	1.65	2.2	0.09	1.08	125	126	9.40	8.71	2	2	10	6	0	0
ZnIr	30	77	12	9	1.65	2.2	0.09	1.57	125	127	9.40	9.12	2	1	10	8	0	0
ZnPt	30	78	12	10	1.65	2.28	0.09	2.13	125	130	9.40	9.02	2	1	10	9	0	0
ZnAu	30	79	12	11	1.65	2.54	0.09	2.31	125	134	9.40	9.23	2	1	10	10	0	0
ZnBi	30	83	12	5	1.65	2.02	0.09	0.95	125	146	9.40	7.29	2	2	10	0	0	3
GaY	31	39	3	11	1.81	1.22	0.30	0.75	126	162	6.00	6.39	2	4	0	1	1	6
GaZr	31	40	3	4	1.81	1.33	0.30	0.43	126	145	6.00	6.84	2	4	0	2	1	6
GaNb	31	41	3	11	1.81	1.6	0.30	0.92	126	134	6.00	6.89	2	1	0	4	1	6
GaMo	31	42	3	6	1.81	2.16	0.30	0.75	126	130	6.00	7.10	2	1	0	5	1	6
GaRu	31	44	3	8	1.81	2.2	0.30	1.05	126	125	6.00	7.37	2	1	0	7	1	0
GaRh	31	45	3	9	1.81	2.28	0.30	1.14	126	125	6.00	7.47	2	1	0	8	1	0
GaPd	31	46	3	10	1.81	2.2	0.30	0.56	126	128	6.00	8.35	2	1	0	9	1	0
GaAg	31	47	3	11	1.81	1.93	0.30	1.30	126	134	6.00	7.58	2	1	0	10	1	0
GaSn	31	50	3	4	1.81	1.96	0.30	1.20	126	140	6.00	7.35	2	2	0	0	1	2
GaHf	31	72	3	4	1.81	1.3	0.30	0.18	126	144	6.00	7.00	2	2	0	2	1	0

GaTa	31	73	3	11	1.81	1.5	0.30	0.32	126	134	6.00	7.89	2	2	0	3	1	0
GaW	31	74	3	6	1.81	2.38	0.30	0.82	126	130	6.00	7.98	2	2	0	4	1	0
GaRe	31	75	3	7	1.81	1.9	0.30	0.06	126	128	6.00	7.88	2	2	0	5	1	6
GaOs	31	76	3	8	1.81	2.2	0.30	1.08	126	126	6.00	8.71	2	2	0	6	1	0
GaIr	31	77	3	9	1.81	2.2	0.30	1.57	126	127	6.00	9.12	2	1	0	8	1	0
GaPt	31	78	3	10	1.81	2.28	0.30	2.13	126	130	6.00	9.02	2	1	0	9	1	0
GaAu	31	79	3	11	1.81	2.54	0.30	2.31	126	134	6.00	9.23	2	1	0	10	1	0
GaBi	31	83	3	5	1.81	2.02	0.30	0.95	126	146	6.00	7.29	2	2	0	0	1	3
YZr	39	40	11	4	1.22	1.33	0.75	0.43	162	145	6.39	6.84	4	4	1	2	6	6
YNb	39	41	11	11	1.22	1.6	0.75	0.92	162	134	6.39	6.89	4	1	1	4	6	6
YMo	39	42	11	6	1.22	2.16	0.75	0.75	162	130	6.39	7.10	4	1	1	5	6	6
YRu	39	44	11	8	1.22	2.2	0.75	1.05	162	125	6.39	7.37	4	1	1	7	6	0
YRh	39	45	11	9	1.22	2.28	0.75	1.14	162	125	6.39	7.47	4	1	1	8	6	0
YPd	39	46	11	10	1.22	2.2	0.75	0.56	162	128	6.39	8.35	4	1	1	9	6	0
YAg	39	47	11	11	1.22	1.93	0.75	1.30	162	134	6.39	7.58	4	1	1	10	6	0
YSn	39	50	11	4	1.22	1.96	0.75	1.20	162	140	6.39	7.35	4	2	1	0	6	2
YTa	39	73	11	11	1.22	1.5	0.75	0.32	162	134	6.39	7.89	4	2	1	3	6	0
YW	39	74	11	6	1.22	2.38	0.75	0.82	162	130	6.39	7.98	4	2	1	4	6	0
YRe	39	75	11	7	1.22	1.9	0.75	0.06	162	128	6.39	7.88	4	2	1	5	6	6
YOs	39	76	11	8	1.22	2.2	0.75	1.08	162	126	6.39	8.71	4	2	1	6	6	0
YIr	39	77	11	9	1.22	2.2	0.75	1.57	162	127	6.39	9.12	4	1	1	8	6	0
YPt	39	78	11	10	1.22	2.28	0.75	2.13	162	130	6.39	9.02	4	1	1	9	6	0
YAu	39	79	11	11	1.22	2.54	0.75	2.31	162	134	6.39	9.23	4	1	1	10	6	0
YBi	39	83	11	5	1.22	2.02	0.75	0.95	162	146	6.39	7.29	4	2	1	0	6	3
ZrMo	40	42	4	6	1.33	2.16	0.43	0.75	145	130	6.84	7.10	4	1	2	5	6	6
ZrRu	40	44	4	8	1.33	2.2	0.43	1.05	145	125	6.84	7.37	4	1	2	7	6	0
ZrRh	40	45	4	9	1.33	2.28	0.43	1.14	145	125	6.84	7.47	4	1	2	8	6	0
ZrPd	40	46	4	10	1.33	2.2	0.43	0.56	145	128	6.84	8.35	4	1	2	9	6	0
ZrAg	40	47	4	11	1.33	1.93	0.43	1.30	145	134	6.84	7.58	4	1	2	10	6	0
ZrSn	40	50	4	4	1.33	1.96	0.43	1.20	145	140	6.84	7.35	4	2	2	0	6	2
ZrHf	40	72	4	4	1.33	1.3	0.43	0.18	145	144	6.84	7.00	4	2	2	2	6	0
ZrTa	40	73	4	11	1.33	1.5	0.43	0.32	145	134	6.84	7.89	4	2	2	3	6	0
ZrW	40	74	4	6	1.33	2.38	0.43	0.82	145	130	6.84	7.98	4	2	2	4	6	0
ZrRe	40	75	4	7	1.33	1.9	0.43	0.06	145	128	6.84	7.88	4	2	2	5	6	6
ZrOs	40	76	4	8	1.33	2.2	0.43	1.08	145	126	6.84	8.71	4	2	2	6	6	0
ZrIr	40	77	4	9	1.33	2.2	0.43	1.57	145	127	6.84	9.12	4	1	2	8	6	0
ZrPt	40	78	4	10	1.33	2.28	0.43	2.13	145	130	6.84	9.02	4	1	2	9	6	0
ZrAu	40	79	4	11	1.33	2.54	0.43	2.31	145	134	6.84	9.23	4	1	2	10	6	0
ZrBi	40	83	4	5	1.33	2.02	0.43	0.95	145	146	6.84	7.29	4	2	2	0	6	3
NbMo	41	42	11	6	1.6	2.16	0.92	0.75	134	130	6.89	7.10	1	1	4	5	6	6
NbRu	41	44	11	8	1.6	2.2	0.92	1.05	134	125	6.89	7.37	1	1	4	7	6	0
NbRh	41	45	11	9	1.6	2.28	0.92	1.14	134	125	6.89	7.47	1	1	4	8	6	0
NbPd	41	46	11	10	1.6	2.2	0.92	0.56	134	128	6.89	8.35	1	1	4	9	6	0
NbAg	41	47	11	11	1.6	1.93	0.92	1.30	134	134	6.89	7.58	1	1	4	10	6	0
NbSn	41	50	11	4	1.6	1.96	0.92	1.20	134	140	6.89	7.35	1	2	4	0	6	2
NbHf	41	72	11	4	1.6	1.3	0.92	0.18	134	144	6.89	7.00	1	2	4	2	6	0
NbTa	41	73	11	11	1.6	1.5	0.92	0.32	134	134	6.89	7.89	1	2	4	3	6	0

NbW	41	74	11	6	1.6	2.38	0.92	0.82	134	130	6.89	7.98	1	2	4	4	6	0
NbRe	41	75	11	7	1.6	1.9	0.92	0.06	134	128	6.89	7.88	1	2	4	5	6	6
NbOs	41	76	11	8	1.6	2.2	0.92	1.08	134	126	6.89	8.71	1	2	4	6	6	0
NbIr	41	77	11	9	1.6	2.2	0.92	1.57	134	127	6.89	9.12	1	1	4	8	6	0
NbPt	41	78	11	10	1.6	2.28	0.92	2.13	134	130	6.89	9.02	1	1	4	9	6	0
NbAu	41	79	11	11	1.6	2.54	0.92	2.31	134	134	6.89	9.23	1	1	4	10	6	0
NbBi	41	83	11	5	1.6	2.02	0.92	0.95	134	146	6.89	7.29	1	2	4	0	6	3
MoRu	42	44	6	8	2.16	2.2	0.75	1.05	130	125	7.10	7.37	1	1	5	7	6	0
MoRh	42	45	6	9	2.16	2.28	0.75	1.14	130	125	7.10	7.47	1	1	5	8	6	0
MoPd	42	46	6	10	2.16	2.2	0.75	0.56	130	128	7.10	8.35	1	1	5	9	6	0
MoAg	42	47	6	11	2.16	1.93	0.75	1.30	130	134	7.10	7.58	1	1	5	10	6	0
MoSn	42	50	6	4	2.16	1.96	0.75	1.20	130	140	7.10	7.35	1	2	5	0	6	2
MoHf	42	72	6	4	2.16	1.3	0.75	0.18	130	144	7.10	7.00	1	2	5	2	6	0
MoTa	42	73	6	11	2.16	1.5	0.75	0.32	130	134	7.10	7.89	1	2	5	3	6	0
MoW	42	74	6	6	2.16	2.38	0.75	0.82	130	130	7.10	7.98	1	2	5	4	6	0
MoRe	42	75	6	7	2.16	1.9	0.75	0.06	130	128	7.10	7.88	1	2	5	5	6	6
MoOs	42	76	6	8	2.16	2.2	0.75	1.08	130	126	7.10	8.71	1	2	5	6	6	0
MoIr	42	77	6	9	2.16	2.2	0.75	1.57	130	127	7.10	9.12	1	1	5	8	6	0
MoPt	42	78	6	10	2.16	2.28	0.75	2.13	130	130	7.10	9.02	1	1	5	9	6	0
MoAu	42	79	6	11	2.16	2.54	0.75	2.31	130	134	7.10	9.23	1	1	5	10	6	0
MoBi	42	83	6	5	2.16	2.02	0.75	0.95	130	146	7.10	7.29	1	2	5	0	6	3
RuRh	44	45	8	9	2.2	2.28	1.05	1.14	125	125	7.37	7.47	1	1	7	8	0	0
RuPd	44	46	8	10	2.2	2.2	1.05	0.56	125	128	7.37	8.35	1	1	7	9	0	0
RuAg	44	47	8	11	2.2	1.93	1.05	1.30	125	134	7.37	7.58	1	1	7	10	0	0
RuSn	44	50	8	4	2.2	1.96	1.05	1.20	125	140	7.37	7.35	1	2	7	0	0	2
RuHf	44	72	8	4	2.2	1.3	1.05	0.18	125	144	7.37	7.00	1	2	7	2	0	0
RuTa	44	73	8	11	2.2	1.5	1.05	0.32	125	134	7.37	7.89	1	2	7	3	0	0
RuW	44	74	8	6	2.2	2.38	1.05	0.82	125	130	7.37	7.98	1	2	7	4	0	0
RuRe	44	75	8	7	2.2	1.9	1.05	0.06	125	128	7.37	7.88	1	2	7	5	0	6
RuOs	44	76	8	8	2.2	2.2	1.05	1.08	125	126	7.37	8.71	1	2	7	6	0	0
RuIr	44	77	8	9	2.2	2.2	1.05	1.57	125	127	7.37	9.12	1	1	7	8	0	0
RuPt	44	78	8	10	2.2	2.28	1.05	2.13	125	130	7.37	9.02	1	1	7	9	0	0
RuAu	44	79	8	11	2.2	2.54	1.05	2.31	125	134	7.37	9.23	1	1	7	10	0	0
RuBi	44	83	8	5	2.2	2.02	1.05	0.95	125	146	7.37	7.29	1	2	7	0	0	3
RhPd	45	46	9	10	2.28	2.2	1.14	0.56	125	128	7.47	8.35	1	1	8	9	0	0
RhAg	45	47	9	11	2.28	1.93	1.14	1.30	125	134	7.47	7.58	1	1	8	10	0	0
RhSn	45	50	9	4	2.28	1.96	1.14	1.20	125	140	7.47	7.35	1	2	8	0	0	2
RhHf	45	72	9	4	2.28	1.3	1.14	0.18	125	144	7.47	7.00	1	2	8	2	0	0
RhTa	45	73	9	11	2.28	1.5	1.14	0.32	125	134	7.47	7.89	1	2	8	3	0	0
RhW	45	74	9	6	2.28	2.38	1.14	0.82	125	130	7.47	7.98	1	2	8	4	0	0
RhRe	45	75	9	7	2.28	1.9	1.14	0.06	125	128	7.47	7.88	1	2	8	5	0	6
RhOs	45	76	9	8	2.28	2.2	1.14	1.08	125	126	7.47	8.71	1	2	8	6	0	0
RhIr	45	77	9	9	2.28	2.2	1.14	1.57	125	127	7.47	9.12	1	1	8	8	0	0
RhPt	45	78	9	10	2.28	2.28	1.14	2.13	125	130	7.47	9.02	1	1	8	9	0	0
RhAu	45	79	9	11	2.28	2.54	1.14	2.31	125	134	7.47	9.23	1	1	8	10	0	0
RhBi	45	83	9	5	2.28	2.02	1.14	0.95	125	146	7.47	7.29	1	2	8	0	0	3
PdAg	46	47	10	11	2.2	1.93	0.56	1.30	128	134	8.35	7.58	1	1	9	10	0	0

PdSn	46	50	10	4	2.2	1.96	0.56	1.20	128	140	8.35	7.35	1	2	9	0	0	2
PdHf	46	72	10	4	2.2	1.3	0.56	0.18	128	144	8.35	7.00	1	2	9	2	0	0
PdTa	46	73	10	11	2.2	1.5	0.56	0.32	128	134	8.35	7.89	1	2	9	3	0	0
PdW	46	74	10	6	2.2	2.38	0.56	0.82	128	130	8.35	7.98	1	2	9	4	0	0
PdRe	46	75	10	7	2.2	1.9	0.56	0.06	128	128	8.35	7.88	1	2	9	5	0	6
PdOs	46	76	10	8	2.2	2.2	0.56	1.08	128	126	8.35	8.71	1	2	9	6	0	0
PdIr	46	77	10	9	2.2	2.2	0.56	1.57	128	127	8.35	9.12	1	1	9	8	0	0
PdPt	46	78	10	10	2.2	2.28	0.56	2.13	128	130	8.35	9.02	1	1	9	9	0	0
PdAu	46	79	10	11	2.2	2.54	0.56	2.31	128	134	8.35	9.23	1	1	9	10	0	0
PdBi	46	83	10	5	2.2	2.02	0.56	0.95	128	146	8.35	7.29	1	2	9	0	0	3
AgSn	47	50	11	4	1.93	1.96	1.30	1.20	134	140	7.58	7.35	1	2	10	0	0	2
AgHf	47	72	11	4	1.93	1.3	1.30	0.18	134	144	7.58	7.00	1	2	10	2	0	0
AgTa	47	73	11	11	1.93	1.5	1.30	0.32	134	134	7.58	7.89	1	2	10	3	0	0
AgW	47	74	11	6	1.93	2.38	1.30	0.82	134	130	7.58	7.98	1	2	10	4	0	0
AgRe	47	75	11	7	1.93	1.9	1.30	0.06	134	128	7.58	7.88	1	2	10	5	0	6
AgOs	47	76	11	8	1.93	2.2	1.30	1.08	134	126	7.58	8.71	1	2	10	6	0	0
AgIr	47	77	11	9	1.93	2.2	1.30	1.57	134	127	7.58	9.12	1	1	10	8	0	0
AgPt	47	78	11	10	1.93	2.28	1.30	2.13	134	130	7.58	9.02	1	1	10	9	0	0
AgAu	47	79	11	11	1.93	2.54	1.30	2.31	134	134	7.58	9.23	1	1	10	10	0	0
AgBi	47	83	11	5	1.93	2.02	1.30	0.95	134	146	7.58	7.29	1	2	10	0	0	3
SnHf	50	72	4	4	1.96	1.3	1.20	0.18	140	144	7.35	7.00	2	2	0	2	2	0
SnTa	50	73	4	11	1.96	1.5	1.20	0.32	140	134	7.35	7.89	2	2	0	3	2	0
SnW	50	74	4	6	1.96	2.38	1.20	0.82	140	130	7.35	7.98	2	2	0	4	2	0
SnRe	50	75	4	7	1.96	1.9	1.20	0.06	140	128	7.35	7.88	2	2	0	5	2	6
SnOs	50	76	4	8	1.96	2.2	1.20	1.08	140	126	7.35	8.71	2	2	0	6	2	0
SnIr	50	77	4	9	1.96	2.2	1.20	1.57	140	127	7.35	9.12	2	1	0	8	2	0
SnPt	50	78	4	10	1.96	2.28	1.20	2.13	140	130	7.35	9.02	2	1	0	9	2	0
SnAu	50	79	4	11	1.96	2.54	1.20	2.31	140	134	7.35	9.23	2	1	0	10	2	0
SnBi	50	83	4	5	1.96	2.02	1.20	0.95	140	146	7.35	7.29	2	2	0	0	2	3
HfTa	72	73	4	11	1.3	1.5	0.18	0.32	144	134	7.00	7.89	2	2	2	3	0	0
HfW	72	74	4	6	1.3	2.38	0.18	0.82	144	130	7.00	7.98	2	2	2	4	0	0
HfRe	72	75	4	7	1.3	1.9	0.18	0.06	144	128	7.00	7.88	2	2	2	5	0	6
HfOs	72	76	4	8	1.3	2.2	0.18	1.08	144	126	7.00	8.71	2	2	2	6	0	0
HfIr	72	77	4	9	1.3	2.2	0.18	1.57	144	127	7.00	9.12	2	1	2	8	0	0
HfPt	72	78	4	10	1.3	2.28	0.18	2.13	144	130	7.00	9.02	2	1	2	9	0	0
HfAu	72	79	4	11	1.3	2.54	0.18	2.31	144	134	7.00	9.23	2	1	2	10	0	0
HfBi	72	83	4	5	1.3	2.02	0.18	0.95	144	146	7.00	7.29	2	2	2	0	0	3
TaW	73	74	11	6	1.5	2.38	0.32	0.82	134	130	7.89	7.98	2	2	3	4	0	0
TaRe	73	75	11	7	1.5	1.9	0.32	0.06	134	128	7.89	7.88	2	2	3	5	0	6
TaOs	73	76	11	8	1.5	2.2	0.32	1.08	134	126	7.89	8.71	2	2	3	6	0	0
TaIr	73	77	11	9	1.5	2.2	0.32	1.57	134	127	7.89	9.12	2	1	3	8	0	0
TaPt	73	78	11	10	1.5	2.28	0.32	2.13	134	130	7.89	9.02	2	1	3	9	0	0
TaAu	73	79	11	11	1.5	2.54	0.32	2.31	134	134	7.89	9.23	2	1	3	10	0	0
TaBi	73	83	11	5	1.5	2.02	0.32	0.95	134	146	7.89	7.29	2	2	3	0	0	3
WRe	74	75	6	7	2.38	1.9	0.82	0.06	130	128	7.98	7.88	2	2	4	5	0	6
WOs	74	76	6	8	2.38	2.2	0.82	1.08	130	126	7.98	8.71	2	2	4	6	0	0
WIr	74	77	6	9	2.38	2.2	0.82	1.57	130	127	7.98	9.12	2	1	4	8	0	0

WPt	74	78	6	10	2.38	2.28	0.82	2.13	130	130	7.98	9.02	2	1	4	9	0	0
WAu	74	79	6	11	2.38	2.54	0.82	2.31	130	134	7.98	9.23	2	1	4	10	0	0
WBi	74	83	6	5	2.38	2.02	0.82	0.95	130	146	7.98	7.29	2	2	4	0	0	3
ReOs	75	76	7	8	1.9	2.2	0.06	1.08	128	126	7.88	8.71	2	2	5	6	6	0
ReIr	75	77	7	9	1.9	2.2	0.06	1.57	128	127	7.88	9.12	2	1	5	8	6	0
RePt	75	78	7	10	1.9	2.28	0.06	2.13	128	130	7.88	9.02	2	1	5	9	6	0
ReAu	75	79	7	11	1.9	2.54	0.06	2.31	128	134	7.88	9.23	2	1	5	10	6	0
ReBi	75	83	7	5	1.9	2.02	0.06	0.95	128	146	7.88	7.29	2	2	5	0	6	3
OsIr	76	77	8	9	2.2	2.2	1.08	1.57	126	127	8.71	9.12	2	1	6	8	0	0
OsPt	76	78	8	10	2.2	2.28	1.08	2.13	126	130	8.71	9.02	2	1	6	9	0	0
OsAu	76	79	8	11	2.2	2.54	1.08	2.31	126	134	8.71	9.23	2	1	6	10	0	0
OsBi	76	83	8	5	2.2	2.02	1.08	0.95	126	146	8.71	7.29	2	2	6	0	0	3
IrPt	77	78	9	10	2.2	2.28	1.57	2.13	127	130	9.12	9.02	1	1	8	9	0	0
IrAu	77	79	9	11	2.2	2.54	1.57	2.31	127	134	9.12	9.23	1	1	8	10	0	0
IrBi	77	83	9	5	2.2	2.02	1.57	0.95	127	146	9.12	7.29	1	2	8	0	0	3
PtAu	78	79	10	11	2.28	2.54	2.13	2.31	130	134	9.02	9.23	1	1	9	10	0	0
PtBi	78	83	10	5	2.28	2.02	2.13	0.95	130	146	9.02	7.29	1	2	9	0	0	3
AuBi	79	83	11	5	2.54	2.02	2.31	0.95	134	146	9.23	7.29	1	2	10	0	0	3
AlAl	13	13	3	3	1.61	1.61	0.44	0.44	118	118	5.99	5.99	1	1	0	0	2	2
ScSc	21	21	3	3	1.36	1.36	0.19	0.19	144	144	6.54	6.54	2	2	1	1	6	6
TiTi	22	22	4	4	1.54	1.54	0.08	0.08	132	132	6.82	6.82	2	2	2	2	0	0
VV	23	23	5	5	1.63	1.63	0.53	0.53	122	122	6.74	6.74	1	1	4	4	6	6
CrCr	24	24	6	6	1.66	1.66	0.68	0.68	118	118	6.77	6.77	1	1	5	5	0	0
MnMn	25	25	7	7	1.55	1.55	-0.50	-0.50	117	117	7.44	7.44	1	1	6	6	0	0
FeFe	26	26	8	8	1.83	1.83	0.15	0.15	117	117	7.87	7.87	1	1	7	7	0	0
CoCo	27	27	9	9	1.88	1.88	0.66	0.66	116	116	7.86	7.86	1	1	8	8	0	0
NiNi	28	28	10	10	1.91	1.91	1.16	1.16	115	115	7.64	7.64	2	2	8	8	0	0
CuCu	29	29	11	11	1.9	1.9	1.24	1.24	117	117	7.73	7.73	1	1	10	10	0	0
ZnZn	30	30	12	12	1.65	1.65	0.09	0.09	125	125	9.40	9.40	2	2	10	10	0	0
GaGa	31	31	3	3	1.81	1.81	0.30	0.30	126	126	6.00	6.00	2	2	0	0	1	1
YY	39	39	11	11	1.22	1.22	0.75	0.75	162	162	6.39	6.39	4	4	1	1	6	6
ZrZr	40	40	4	4	1.33	1.33	0.43	0.43	145	145	6.84	6.84	4	4	2	2	6	6
NbNb	41	41	11	11	1.6	1.6	0.92	0.92	134	134	6.89	6.89	1	1	4	4	6	6
MoMo	42	42	6	6	2.16	2.16	0.75	0.75	130	130	7.10	7.10	1	1	5	5	6	6
RuRu	44	44	8	8	2.2	2.2	1.05	1.05	125	125	7.37	7.37	1	1	7	7	0	0
RhRh	45	45	9	9	2.28	2.28	1.14	1.14	125	125	7.47	7.47	1	1	8	8	0	0
PdPd	46	46	10	10	2.2	2.2	0.56	0.56	128	128	8.35	8.35	1	1	9	9	0	0
AgAg	47	47	11	11	1.93	1.93	1.30	1.30	134	134	7.58	7.58	1	1	10	10	0	0
SnSn	50	50	4	4	1.96	1.96	1.20	1.20	140	140	7.35	7.35	2	2	0	0	2	2
HfHf	72	72	4	4	1.3	1.3	0.18	0.18	144	144	7.00	7.00	2	2	2	2	0	0
TaTa	73	73	11	11	1.5	1.5	0.32	0.32	134	134	7.89	7.89	2	2	3	3	0	0
WW	74	74	6	6	2.38	2.38	0.82	0.82	130	130	7.98	7.98	2	2	4	4	0	0
ReRe	75	75	7	7	1.9	1.9	0.06	0.06	128	128	7.88	7.88	2	2	5	5	6	6
OsOs	76	76	8	8	2.2	2.2	1.08	1.08	126	126	8.71	8.71	2	2	6	6	0	0
IrIr	77	77	9	9	2.2	2.2	1.57	1.57	127	127	9.12	9.12	1	1	8	8	0	0
PtPt	78	78	10	10	2.28	2.28	2.13	2.13	130	130	9.02	9.02	1	1	9	9	0	0
AuAu	79	79	11	11	2.54	2.54	2.31	2.31	134	134	9.23	9.23	1	1	10	10	0	0

Table S4. Adsorption Energies (Eads, eV) of Key Intermediates ($*CO_2$, *COOH, *CO, *CHO) in Dual-Atom Catalysts (DACs) during electrocatalytic CO_2 reduction reaction (CO_2RR).

Metal	E_b -CO	E_b -CO_2	E_b -COOH	E_b -CHO
AlRh	-1.97	-1.59	-2.50	-2.73
AlPd	-0.76	-1.27	-0.99	-1.02
AlIr	-2.48	-1.98	-2.83	-3.09
AlPt	-0.57	-1.32	-1.14	-1.26
TiRh	0.00	-0.37	-1.02	-1.00
TiPd	-1.25	-1.31	-1.94	-2.11
TiIr	-1.16	-2.00	-1.94	-2.24
TiPt	-1.12	-1.37	-1.95	-2.26
VCu	-0.14	-0.80	-1.04	-1.20
VRu	-1.68	-1.57	-1.65	-1.88
VRh	-0.70	-1.41	-1.46	-1.83
VPd	-0.84	-0.69	-0.99	-1.36
VIr	-0.74	-1.52	-1.64	-2.03
VPt	-0.16	-0.78	-1.07	-1.24
VAu	-0.13	-0.84	-1.15	-1.25
CrNi	-0.97	-0.67	-0.68	-0.92
CrRh	-0.76	0.00	-0.49	-1.24
CrPd	-1.12	-0.67	-0.58	-0.84
CrPt	-1.34	-0.05	-0.49	-1.07
MnRh	-1.88	-1.01	-1.39	-1.73
MnPd	-0.48	-0.21	-0.05	-0.32
MnIr	-1.92	-0.84	-1.51	-1.82
FeCo	-1.47	-0.08	-0.62	-0.96
FeNi	-1.39	-0.33	-0.38	-0.86
FeCu	-1.35	-0.01	-0.32	-1.29
FeRu	-1.94	-0.71	-1.25	-1.49
FeRh	-1.67	-0.64	-0.78	-1.12
FeAg	-1.37	-0.10	-0.51	-1.05
FeIr	-1.84	-0.68	-0.24	-1.28
FePt	-1.34	-0.11	-0.33	-1.54
CoNi	-1.56	-0.05	-0.72	-1.23
CoGa	-1.43	-0.56	-0.70	-1.02
CoRh	-2.04	-0.42	-0.84	-1.26
CoPd	-0.37	-0.04	-0.07	-0.35
CoIr	-2.25	-0.43	-1.09	-1.43
CoPt	-1.64	-0.01	-0.37	-1.22
NiZn	-1.04	-0.26	-0.38	-0.75
NiGa	-0.68	-0.41	-0.16	-0.32
NiRu	-2.23	-0.14	-1.19	-1.45
NiRh	-1.76	-0.20	-0.76	-1.40
NiIr	-2.05	-0.14	-0.88	-1.27

CuRu	-2.10	-0.33	-1.36	-1.64
ZnRu	-1.99	-1.25	-1.82	-2.08
ZnRh	-1.55	-0.82	-0.78	-1.01
ZnIr	-2.04	-0.96	-1.08	-1.31
ZnPt	-1.44	-0.37	-1.15	-1.32
GaRu	-0.03		-0.55	
GaPd	-0.53	-0.28	-0.11	-0.17
GaOs	-2.40	-1.07	-1.33	-1.43
GaPt	-0.65	-0.35	-0.20	-0.12
NbRh	-0.50	-1.10	-1.64	-1.76
NbAg	-0.84	-0.34	-0.79	-0.88
RuRh	-1.55	-0.23	-1.07	-1.44
RuPd	-2.17	-0.71	-1.06	-1.49
RhPd	-1.73	-0.36	-0.09	-1.32
RhTa	-1.44	-1.24	-3.31	-2.17
RhPt	-1.78	-0.13	-0.94	-1.31
PdTa	-1.39	-3.03	-3.21	-2.13
IrPt	-1.96	-0.59	-0.12	-1.38
CoCo	-1.90	-0.08	-0.69	-1.19
RhRh	-1.94	-0.07	-0.34	-0.54
IrIr	-1.95	-0.04	-0.50	-0.95

Table S5. Calculated adsorption structure parameters of adsorption (M-C bond length (d_{C-M1}, d_{C-M2} in Å, M-O bond length (d_{O1-M1}, d_{O2_M1} in Å) , M1-M2 bond length (d_{M1-M2} in Å), and Carbon dioxide activation Angle (∠O1CO2, °)) during the adsorption of CO_2.

Metal	d_{C-M1}	d_{C-M2}	d_{O1-M1}	d_{O1-M2}	d_{O2-M1}	d_{O2-M2}	d_{M1-M2}	∠O1CO2
AlRh	2.43	2.05	1.81	2.86	3.64	2.86	2.47	126.88
AlPd	2.34	2.06	1.82	2.95	3.56	2.73	2.51	129.69
AlIr	2.26	2.05	1.81	2.99	3.49	2.78	2.46	124.78
AlPt	2.31	2.08	1.81	2.98	3.53	2.78	2.49	127.53
TiRh	2.15	3.42	2.07	3.65	3.27	4.17	2.32	138.77
TiPd	2.37	2.11	1.92	3.02	3.59	2.79	2.49	127.69
TiIr	2.35	2.05	1.90	2.94	3.57	2.83	2.32	124.38
TiPt	2.39	2.10	1.91	3.00	3.60	2.82	2.48	126.46
VCu	2.04	2.54	1.89	3.51	3.23	3.05	2.46	130.10
VRu	2.36	2.04	1.87	2.79	3.58	2.92	2.09	125.98
VRh	2.35	2.03	1.83	2.90	3.56	2.84	2.35	124.36
VPd	2.00	3.07	1.96	3.53	3.13	3.74	2.29	136.71
VIr	2.34	2.03	1.84	2.92	3.55	2.85	2.35	123.34
VPt	2.00	3.25	1.97	3.52	3.12	3.99	2.29	136.98
VAu	1.99	3.02	1.90	3.84	3.18	3.58	2.50	131.44
CrNi	2.48	1.95	1.96	2.78	3.70	2.62	2.44	133.45
CrRh	1.99	3.53	2.00	3.57	3.10	4.33	2.52	139.50
CrPd	2.45	2.08	1.94	2.92	3.66	2.75	2.49	131.79
CrPt	1.99	3.39	1.97	3.40	3.11	4.21	2.41	139.00
MnRh	2.59	2.03	1.93	2.73	3.80	2.86	2.42	130.64
MnPd	2.55	2.18	2.10	2.95	3.73	2.77	2.40	138.87
MnIr	2.55	2.04	1.93	2.78	3.76	2.87	2.40	128.72
FeCo	2.15	2.05	2.20	3.13	3.24	2.17	2.40	139.06
FeNi	2.17	2.07	1.93	3.03	3.34	2.53	2.42	134.17
FeCu	2.23	2.92	2.55	3.95	3.02	2.68	2.45	149.72
FeRu	2.53	2.06	1.94	2.79	3.75	2.89	2.35	129.00
FeRh	2.60	2.07	1.99	2.76	3.81	2.87	2.38	132.89
FeAg	2.00	2.91	2.13	3.85	3.01	3.03	2.43	144.23
FeIr	2.57	2.08	1.99	2.79	3.79	2.87	2.36	131.71
FePt	3.24	3.40	2.80	3.65	4.02	3.58	2.34	177.84
CoNi	2.15	2.38	2.11	3.31	3.23	2.63	2.40	141.57
CoGa	1.91	2.30	2.47	3.52	2.91	1.99	2.40	130.62
CoRh	2.62	2.09	2.00	2.77	3.83	2.86	2.41	134.72
CoPd	2.54	2.20	2.11	2.97	3.71	2.75	2.40	140.71
CoIr	2.59	2.10	2.01	2.81	3.81	2.87	2.39	133.18
CoPt	2.54	2.18	2.07	2.94	3.74	2.80	2.41	137.04
NiZn	1.98	2.61	2.63	3.82	2.76	2.05	2.52	136.72
NiGa	1.94	2.41	2.57	3.62	2.84	1.98	2.39	132.28
NiRu	2.49	2.06	2.84	3.19	3.43	2.06	2.42	135.49
NiRh	3.47	2.19	2.99	2.69	4.59	2.89	2.44	146.71
NiIr	3.41	2.19	3.05	2.74	4.50	2.89	2.42	143.64

CuRu	2.92	2.08	2.73	2.95	4.03	2.48	2.45	142.38
ZnRu	2.66	2.06	1.93	2.80	3.86	2.90	2.65	127.48
ZnRh	2.71	2.08	1.96	2.77	3.91	2.87	2.67	132.33
ZnIr	2.67	2.08	1.94	2.82	3.87	2.88	2.69	129.91
ZnPt	2.48	2.10	2.00	2.98	3.70	2.76	2.55	130.37
GaPd	2.41	2.08	1.96	2.96	3.63	2.73	2.46	130.84
GaOs	2.50	2.07	1.91	2.89	3.71	2.92	2.47	123.28
GaPt	2.40	2.09	1.95	2.98	3.62	2.78	2.45	128.37
NbRh	2.13	3.56	2.12	3.75	3.27	4.37	2.33	134.17
NbAg	2.13	4.78	2.11	4.87	3.26	5.76	2.78	134.40
RuRh	2.15	3.44	2.77	4.45	2.79	3.29	2.36	144.94
RuPd	2.03	3.55	3.01	4.70	2.25	2.86	2.52	142.13
RhPd	2.67	2.11	2.25	2.94	3.87	2.70	2.51	136.07
RhTa	3.65	2.12	4.56	3.29	3.72	2.06	2.35	130.52
RhPt	2.17	3.64	2.89	4.65	2.62	3.32	2.48	148.48
PdTa	2.20	2.18	2.96	3.33	3.80	1.75	2.57	127.82
IrPt	2.59	2.06	2.05	2.89	3.81	2.82	2.53	127.61
CoCo	1.99	2.17	2.18	3.31	3.06	2.13	2.44	137.75
RhRh	2.27	3.63	2.89	4.53	2.77	3.46	2.48	150.30
IrIr	2.26	3.58	2.82	4.54	2.90	3.44	2.44	146.77

Table S6. Calculated bader charge (Q , |e|) transferred from the diatomic catalyst to the adsorbed carbon dioxide molecule and the charges transferred to C, O1 and O2 atoms respectively, and the corresponding carbon dioxide activation Angle (∠O1CO2, °).

Metal	C_bader	O1_bader	O2_bader	CO2-bader	∠O1CO2
AlRh	0.21	0.52	0.15	0.88	126.88
AlPd	0.23	0.44	0.17	0.84	129.69
AlIr	0.31	0.53	0.22	1.06	124.78
AlPt	0.2	0.52	0.16	0.88	127.53
TiRh	0.37	0.23	0.14	0.73	138.77
TiPd	0.26	0.27	0.14	0.67	127.69
TiIr	0.22	0.34	0.18	0.73	124.38
TiPt	0.21	0.29	0.16	0.65	126.46
VCu	0.42	0.23	0.2	0.85	130.1
VRu	0.24	0.3	0.17	0.71	125.98
VRh	0.23	0.29	0.16	0.67	124.36
VPd	0.36	0.18	0.16	0.7	136.71
VIr	0.23	0.29	0.17	0.69	123.34
VPt	0.35	0.18	0.15	0.69	136.98
VAu	0.36	0.26	0.19	0.81	131.44
CrNi	0.25	0.21	0.12	0.58	133.45
CrRh	0.33	0.17	0.15	0.65	139.5
CrPd	0.23	0.21	0.13	0.58	131.79
CrPt	0.35	0.14	0.14	0.63	139
MnRh	0.18	0.26	0.15	0.59	130.64
MnPd	0.18	0.17	0.12	0.47	138.87
MnIr	0.16	0.28	0.16	0.61	128.72
FeCo	0.33	0.14	0.15	0.63	139.06
FeNi	0.27	0.2	0.15	0.62	134.17
FeCu	0.21	0.1	0.12	0.42	149.72
FeRu	0.24	0.24	0.16	0.64	129
FeRh	0.15	0.25	0.15	0.56	132.89
FeAg	0.26	0.12	0.14	0.52	144.23
FeIr	0.16	0.24	0.16	0.56	131.71
FePt	0	0.04	0.01	0.06	177.84
CoNi	0.24	0.13	0.13	0.51	141.57
CoGa	0.31	0.17	0.34	0.81	130.62
CoRh	0.15	0.23	0.15	0.53	134.72
CoPd	0.15	0.15	0.12	0.43	140.71
CoIr	0.18	0.2	0.16	0.53	133.18
CoPt	0.16	0.16	0.13	0.46	137.04
NiZn	0.2	0.13	0.25	0.59	136.72
NiGa	0.25	0.13	0.27	0.65	132.28
NiRu	0.25	0.18	0.17	0.6	135.49
NiRh	0.16	0.12	0.12	0.4	146.71
NiIr	0.15	0.14	0.15	0.44	143.64

CuRu	0.22	0.17	0.13	0.51	142.38
ZnRu	0.22	0.31	0.19	0.73	127.48
ZnRh	0.11	0.31	0.14	0.56	132.33
ZnIr	0.13	0.32	0.17	0.63	129.91
ZnPt	0.17	0.34	0.19	0.7	130.37
GaPd	0.21	0.29	0.14	0.63	130.84
GaOs	0.2	0.38	0.2	0.78	123.28
GaPt	0.18	0.32	0.16	0.67	128.37
NbRh	0.38	0.31	0.18	0.87	134.17
NbAg	0.38	0.26	0.16	0.81	134.4
RuRh	0.22	0.13	0.13	0.48	144.94
RuPd	0.23	0.13	0.17	0.52	142.13
RhPd	0.2	0.14	0.13	0.47	136.07
RhTa	0.42	0.2	0.31	0.93	130.52
RhPt	0.16	0.1	0.11	0.37	148.48
PdTa	0.39	0.01	0.72	1.12	127.82
IrPt	0.19	0.15	0.16	0.5	127.61
CoCo	0.32	0.14	0.17	0.64	137.75
RhRh	0.14	0.11	0.11	0.36	150.3
IrIr	0.15	0.13	0.12	0.4	146.77

Table S7. Calculated COHP between carbon dioxide adsorbed on diatomic catalyst and bimetal. There are six possible interactions between two metal atoms and three carbon dioxide atoms.

COHP	atom	atom	distance (Å)	ICOHP (at) E_F
1.000	C	V70	1.995	-3.014
2.000	O1	V70	1.899	-3.222
3.000	O2	V70	3.177	-0.227
4.000	C	Au71	3.018	-0.197
5.000	O1	Au71	3.835	-0.061
6.000	O2	Au71	3.581	-0.024
Average				-1.124
1.000	C	V70	2.038	-2.705
2.000	O1	V70	1.886	-3.394
3.000	O2	V70	3.231	-0.226
4.000	C	Cu71	2.542	-0.420
5.000	O1	Cu71	3.514	-0.049
6.000	O2	Cu71	3.052	-0.046
Average				-1.140
1.000	C	Ru70	2.152	-2.452
2.000	O1	Ru70	2.766	-0.130
3.000	O2	Ru70	2.792	-0.132
4.000	C	Rh71	3.445	-0.087
5.000	O1	Rh71	4.450	-0.029
6.000	O2	Rh71	3.289	-0.013
Average				-0.474
1.000	C	Zn70	2.479	-0.290
2.000	O1	Zn70	2.005	-0.998
3.000	O2	Zn70	3.704	-0.142
4.000	C	Pt71	2.102	-3.058
5.000	O1	Pt71	2.975	-0.155
6.000	O2	Pt71	2.757	-0.087
Average				-0.788
1.000	C	Nb70	2.127	-2.880
2.000	O1	Nb70	2.111	-3.369
3.000	O2	Nb70	3.263	-0.202
4.000	C	Ag71	4.782	0.071
5.000	O1	Ag71	4.875	-0.094
6.000	O2	Ag71	5.757	0.025
Average				-1.075
1.000	C	Zn70	2.656	-0.075
2.000	O1	Zn70	1.927	-1.625
3.000	O2	Zn70	3.861	-0.085
4.000	C	Ru71	2.061	-3.399
5.000	O1	Ru71	2.804	-0.027
6.000	O2	Ru71	2.904	-0.121

Average				-0.889
1.000	C	V70	2.365	-0.700
2.000	O1	V70	1.871	-3.505
3.000	O2	V70	3.576	-0.137
4.000	C	Ru71	2.045	-3.321
5.000	O1	Ru71	2.795	-0.115
6.000	O2	Ru71	2.922	-0.134
Average				-1.319
1.000	C	Al70	2.261	-1.896
2.000	O1	Al70	1.808	-4.232
3.000	O2	Al70	3.488	-0.261
4.000	C	Ir71	2.050	-3.455
5.000	O1	Ir71	2.989	-0.183
6.000	O2	Ir71	2.777	-0.134
Average				-1.693
1.000	C	Mn70	2.546	-0.398
2.000	O1	Mn70	1.929	-2.430
3.000	O2	Mn70	3.760	-0.123
4.000	C	Ir71	2.040	-3.630
5.000	O1	Ir71	2.775	-0.106
6.000	O2	Ir71	2.870	-0.138
Average				-1.138
1.000	C	Al70	2.308	-1.841
2.000	O1	Al70	1.811	-4.230
3.000	O2	Al70	3.526	-0.266
4.000	C	Pt71	2.083	-2.893
5.000	O1	Pt71	2.983	-0.158
6.000	O2	Pt71	2.780	-0.074
Average				-1.577
1.000	C	Cr70	2.483	-0.569
2.000	O1	Cr70	1.956	-2.594
3.000	O2	Cr70	3.696	-0.123
4.000	C	Ni71	1.952	-2.256
5.000	O1	Ni71	2.782	-0.094
6.000	O2	Ni71	2.623	-0.105
Average				-0.957
1.000	C	Al70	2.341	-1.993
2.000	O1	Al70	1.819	-4.217
3.000	O2	Al70	3.557	-0.232
4.000	C	Pd71	2.058	-1.905
5.000	O1	Pd71	2.946	-0.008
6.000	O2	Pd71	2.729	0.012
Average				-1.390
1.000	C	Mn70	2.592	-0.292
2.000	O1	Mn70	1.926	-2.386
3.000	O2	Mn70	3.802	-0.096
4.000	C	Rh71	2.032	-3.105

5.000	O1	Rh71	2.726	-0.055
6.000	O2	Rh71	2.865	-0.134
Average				-1.011
1.000	C	V70	1.995	-2.894
2.000	O1	V70	1.963	-2.535
3.000	O2	V70	3.131	-0.201
4.000	C	Pd71	3.071	-0.094
5.000	O1	Pd71	3.533	-0.020
6.000	O2	Pd71	3.737	-0.010
Average				-0.959
1.000	C	Cr70	1.995	-2.670
2.000	O1	Cr70	2.004	-2.159
3.000	O2	Cr70	3.097	-0.173
4.000	C	Rh71	3.530	-0.096
5.000	O1	Rh71	3.571	-0.013
6.000	O2	Rh71	4.328	-0.009
Average				-0.853
1.000	C	Al70	2.435	-1.162
2.000	O1	Al70	1.809	-4.568
3.000	O2	Al70	3.642	-0.212
4.000	C	Rh71	2.052	-2.935
5.000	O1	Rh71	2.858	-0.046
6.000	O2	Rh71	2.858	-0.078
Average				-1.500

Table S8. Calculated the number of electrons of two metal atoms in different integration regions before and after adsorption of carbon dioxide molecules.

Energy interval		(-1.40,0.15)	(0.15,2.86)	(-10,0)	(-10,10)
Zn-before	up	0.106	0.050	4.844	4.994
	down	0.108	0.049	4.847	4.969
Ru-before	up	0.191	2.617	0.859	4.566
	down	0.119	2.607	0.743	4.537
Zn-after	up	0.190	0.037	4.842	4.986
	down	0.190	0.037	4.842	4.986
Ru-after	up	0.162	2.355	0.972	4.593
	down	0.162	2.356	0.974	4.594
Zn	before	0.214	0.099	9.691	9.963
	after	0.380 (+0.025)	0.074 (-0.025)	9.684	9.972
Ru	before	0.310	5.224	1.602	9.103
	after	0.324 (+0.025)	4.711 (-0.025)	1.946	9.187

Table S9. Structure types of adsorbed intermediates *CO and *CHO by diatomic catalysts.

Metal	*CO	*CHO	Metal	*CO	*CHO
AlIr	end-bridge	side-bridge II	MnIr	end-bridge	side-bridge II
AlPd	side-bridge II	side-bridge II	MnPd	end-on-right	side-bridge II
AlPt	side-bridge II	side-bridge II	MnRh	end-bridge	side-bridge II
AlRh	end-bridge	side-bridge II	NbAg	end-on-left	side-on I
CoCo	end-bridge	end-bridge	NbRh	side-on I	side-on I
CoGa	end-on-left		NiGa	end-on-left	end-on-left
CoIr	end-bridge	end-bridge	NiIr	end-bridge	side-bridge II
CoNi	end-bridge	end-bridge	NiRh	end-bridge	end-bridge
CoPd(COO)	end-on-right	side-bridge II	NiRu	end-bridge	end-on-right
CoPt	end-bridge	end-bridge	NiZn	end-on-left	end-bridge
CoRh	end-bridge	end-bridge	RhPd	end-bridge	end-bridge
CrNi	end-bridge	side-bridge II	RhPt	end-bridge	end-bridge
CrPd	end-bridge	side-bridge II	RuPd	end-bridge	end-on-right
CrPt	end-bridge	end-bridge	RuRh	end-on-left	end-on-right
CrRh	end-on-left	end-bridge	TiIr	side-bridge II	side-bridge II
CuRu	end-on-right	t	TiPd	side-bridge II	side-bridge II
FeAg	end-on-left		TiPt	side-bridge II	side-bridge II
FeCo	end-bridge	end-bridge	TiRh	side-on I	side-on I
FeIr	end-bridge	end-bridge	VAu	side-on I	side-on I
FeNi	end-bridge	end-bridge	VCu	side-on I	side-on I
FePt	end-bridge		VIr	side-bridge II	side-bridge II
FeRh	end-bridge	end-bridge	VPd	side-bridge II	side-bridge II
FeRu	end-bridge	side-bridge II	VPt	side-on I	side-on I
GaOs	end-bridge		VRh	side-bridge II	side-bridge II
GaPd	end-on-right	end-on-right	VRu	end-bridge	side-bridge II
GaPt	end-on-right	end-on-right	ZnIr	end-bridge	side-bridge II
GaRu	end-on-left	end-on-left	ZnPt	end-on-right	side-bridge II
IrIr	end-bridge	end-bridge	ZnRh	end-bridge	side-bridge II
IrPt	end-bridge	end-bridge	ZnRu	end-bridge	side-bridge II

Table S10. The free energy (ΔG/eV) of electrocatalytic CO_2 reduction on TM1/TM2–N@Gra (Key intermediate: CO_2-*COOH-*CO-*CHO).

metal	molecules	E_0/eV	E_ZPE/eV	T*S/eV	ΔE /eV	ΔG/eV
AlRh	slab	-632.18			-655.41	0.00
AlRh	COOH	-660.88	0.65	0.14	-660.36	-1.55
AlRh	CO	-649.32	0.22	0.11	-649.21	0.33
AlRh	CHO	-653.46	0.50	0.09	-653.05	-0.44
	slab	-630.93			-654.17	0.00
AlPd	COOH	-658.13	0.63	0.11	-657.62	-0.05
AlPd	CO	-646.86	0.22	0.07	-646.72	0.08
AlPd	CHO	-650.50	0.48	0.06	-650.08	0.03
	slab	-633.17			-656.40	0.00
AlIr	COOH	-662.20	0.64	0.10	-661.66	-1.86
AlIr	CO	-650.82	0.22	0.06	-650.66	0.18
AlIr	CHO	-654.81	0.52	0.08	-654.37	-0.31
	slab	-631.92			-655.16	0.00
AlPt	COOH	-659.26	0.67	0.13	-658.72	-0.17
AlPt	CO	-647.66	0.21	0.07	-647.52	0.38
AlPt	CHO	-651.74	0.52	0.07	-651.29	-0.36
	slab	-636.50			-659.74	0.00
TiRh	COOH	-663.73	0.62	0.13	-663.23	-0.09
TiRh	CO	-651.67	0.17	0.10	-651.61	0.80
TiRh	CHO	-656.05	0.48	0.13	-655.70	-0.69
	slab	-634.19			-657.43	0.00
TiPd	COOH	-662.34	0.65	0.13	-661.82	-0.99
TiPd	CO	-650.61	0.21	0.08	-650.48	0.52
TiPd	CHO	-654.85	0.50	0.08	-654.43	-0.56
	slab	-637.62			-660.85	0.00
TiIr	COOH	-665.76	0.64	0.11	-665.23	-0.98
TiIr	CO	-653.95	0.19	0.09	-653.85	0.56
TiIr	CHO	-658.41	0.50	0.10	-658.01	-0.77
	slab	-635.23			-658.47	0.00
TiPt	COOH	-663.38	0.65	0.13	-662.86	-0.99
TiPt	CO	-651.52	0.22	0.09	-651.39	0.65
TiPt	CHO	-656.04	0.50	0.05	-655.58	-0.79
	slab	-633.52			-656.76	0.00
VCu	COOH	-660.77	0.64	0.15	-660.28	-0.12
VCu	CO	-648.83	0.18	0.12	-648.77	0.69
VCu	CHO	-653.27	0.47	0.15	-652.95	-0.78
	slab	-638.27			-661.50	0.00
VRu	COOH	-666.12	0.64	0.10	-665.59	-0.68
VRu	CO	-655.12	0.23	0.09	-654.98	-0.21
VRu	CHO	-658.70	0.48	0.12	-658.34	0.04
	slab	-636.67			-659.90	0.00
VRh	COOH	-664.33	0.65	0.14	-663.81	-0.51

VRh	CO	-652.54	0.21	0.07	-652.41	0.59
VRh	CHO	-657.05	0.50	0.09	-656.63	-0.83
	slab	-634.50			-657.73	0.00
VPd	COOH	-661.69	0.64	0.15	-661.21	-0.08
VPd	CO	-650.51	0.20	0.11	-650.42	-0.03
VPd	CHO	-654.41	0.49	0.10	-654.02	-0.20
	slab	-637.81			-661.05	0.00
VIr	COOH	-665.66	0.67	0.11	-665.10	-0.65
VIr	CO	-653.73	0.19	0.09	-653.63	0.66
VIr	CHO	-658.39	0.51	0.08	-657.97	-0.94
	slab	-635.50			-658.73	0.00
VPt	COOH	-662.77	0.64	0.15	-662.27	-0.14
VPt	CO	-650.83	0.17	0.10	-650.76	0.69
VPt	CHO	-655.29	0.47	0.13	-654.94	-0.78
	slab	-631.74			-654.98	0.00
VAu	COOH	-659.09	0.62	0.18	-658.65	-0.27
VAu	CO	-647.04	0.16	0.11	-647.00	0.83
VAu	CHO	-651.55	0.47	0.13	-651.21	-0.81
	slab	-636.26			-659.50	0.00
CrNi	COOH	-663.14	0.64	0.11	-662.61	0.29
CrNi	CO	-652.40	0.22	0.10	-652.28	-0.49
CrNi	CHO	-655.73	0.50	0.12	-655.35	0.33
	slab	-636.98			-660.22	0.00
CrRh	COOH	-663.67	0.61	0.10	-663.17	0.45
CrRh	CO	-652.91	0.22	0.10	-652.80	-0.45
CrRh	CHO	-656.77	0.49	0.10	-656.38	-0.18
	slab	-635.12			-658.35	0.00
CrPd	COOH	-661.90	0.64	0.18	-661.44	0.31
CrPd	CO	-651.41	0.21	0.12	-651.32	-0.69
CrPd	CHO	-654.51	0.49	0.10	-654.13	0.59
	slab	-636.07			-659.30	0.00
CrPt	COOH	-662.76	0.62	0.13	-662.26	0.44
CrPt	CO	-652.58	0.22	0.09	-652.45	-1.01
CrPt	CHO	-655.68	0.48	0.08	-655.28	0.58
	slab	-636.54			-659.78	0.00
MnRh	COOH	-664.14	0.64	0.16	-663.65	-0.47
MnRh	CO	-653.59	0.22	0.09	-653.46	-0.63
MnRh	CHO	-656.82	0.50	0.10	-656.42	0.43
	slab	-635.02			-658.25	0.00
MnPd	COOH	-661.27	0.62	0.12	-660.77	0.88
MnPd	CO	-650.66	0.21	0.12	-650.57	-0.62
MnPd	CHO	-653.89	0.48	0.11	-653.52	0.46
	slab	-637.80			-661.04	0.00
MnIr	COOH	-665.52	0.66	0.14	-665.00	-0.56
MnIr	CO	-654.89	0.21	0.13	-654.82	-0.63
MnIr	CHO	-658.18	0.52	0.10	-657.76	0.45
	slab	-636.09			-659.32	0.00
FeCo	COOH	-662.92	0.65	0.10	-662.36	0.36

FeCo	CO	-652.73	0.11	0.04	-652.67	-1.12
FeCo	CHO	-655.60	0.48	0.11	-655.23	0.84
	slab	-634.75			-657.98	0.00
FeNi	COOH	-661.33	0.63	0.17	-660.87	0.51
FeNi	CO	-651.31	0.11	0.05	-651.25	-1.19
FeNi	CHO	-654.16	0.48	0.11	-653.78	0.86
	slab	-632.59			-655.83	0.00
FeCu	COOH	-659.12	0.63	0.16	-658.65	0.58
FeCu	CO	-649.11	0.23	0.10	-648.99	-1.16
FeCu	CHO	-652.43	0.22	0.13	-652.33	0.05
	slab	-636.84			-660.07	0.00
FeRu	COOH	-664.29	0.65	0.14	-663.78	-0.31
FeRu	CO	-653.95	0.23	0.04	-653.77	-0.81
FeRu	CHO	-656.87	0.51	0.09	-656.45	0.72
	slab	-635.64			-658.87	0.00
FeRh	COOH	-662.62	0.65	0.15	-662.13	0.14
FeRh	CO	-652.48	0.23	0.09	-652.35	-1.04
FeRh	CHO	-655.31	0.50	0.09	-654.90	0.85
	slab	-630.23			-653.47	0.00
FeAg	COOH	-656.94	0.64	0.14	-656.44	0.42
FeAg	CO	-646.77	0.24	0.09	-646.63	-1.00
FeAg	CHO	-649.97	0.23	0.10	-649.84	0.19
	slab	-636.77			-660.01	0.00
FeIr	COOH	-663.21	0.68	0.13	-662.67	0.74
FeIr	CO	-653.79	0.23	0.08	-653.65	-1.80
FeIr	CHO	-656.61	0.49	0.11	-656.22	0.82
	slab	-634.66			-657.90	0.00
FePt	COOH	-661.20	0.65	0.14	-660.68	0.62
FePt	CO	-651.18	0.23	0.09	-651.04	-1.17
FePt	CHO	-654.76	0.24	0.09	-654.61	-0.17
	slab	-633.56			-656.80	0.00
CoNi	COOH	-660.49	0.65	0.13	-659.97	0.23
CoNi	CO	-650.29	0.10	0.05	-650.24	-1.09
CoNi	CHO	-653.34	0.48	0.13	-652.99	0.65
	slab	-630.83			-654.07	0.00
CoGa	COOH	-657.74	0.59	0.16	-657.30	0.17
CoGa	CO	-647.43	0.21	0.14	-647.36	-0.87
CoGa	CHO	-650.41	0.40	0.10	-650.11	0.65
	slab	-634.30			-657.54	0.00
CoRh	COOH	-661.35	0.62	0.13	-660.86	0.08
CoRh	CO	-651.52	0.23	0.09	-651.38	-1.34
CoRh	CHO	-654.11	0.49	0.11	-653.74	1.04
	slab	-632.46			-655.69	0.00
CoPd	COOH	-658.73	0.64	0.17	-658.26	0.83
CoPd	CO	-648.00	0.20	0.12	-647.92	-0.48
CoPd	CHO	-651.36	0.49	0.11	-650.99	0.34
	slab	-635.40			-658.63	0.00
CoIr	COOH	-662.69	0.65	0.15	-662.18	-0.15

CoIr	CO	-652.82	0.21	0.12	-652.73	-1.37
CoIr	CHO	-655.37	0.49	0.11	-654.99	1.14
	slab	-633.46			-656.70	0.00
CoPt	COOH	-660.03	0.66	0.13	-659.50	0.60
CoPt	CO	-650.27	0.23	0.10	-650.15	-1.46
CoPt	CHO	-653.23	0.49	0.13	-652.87	0.68
	slab	-628.13			-651.37	0.00
NiZn	COOH	-654.71	0.63	0.16	-654.24	0.52
NiZn	CO	-644.34	0.21	0.13	-644.26	-0.84
NiZn	CHO	-647.43	0.48	0.11	-647.06	0.60
	slab	-629.89			-653.13	0.00
NiGa	COOH	-656.25	0.57	0.21	-655.90	0.63
NiGa	CO	-645.74	0.22	0.10	-645.63	-0.55
NiGa	CHO	-648.77	0.49	0.11	-648.39	0.64
	slab	-634.08			-657.32	0.00
NiRu	COOH	-661.48	0.61	0.16	-661.02	-0.30
NiRu	CO	-651.48	0.23	0.10	-651.35	-1.15
NiRu	CHO	-654.08	0.48	0.13	-653.73	1.02
	slab	-633.05			-656.29	0.00
NiRh	COOH	-660.01	0.63	0.14	-659.52	0.16
NiRh	CO	-649.98	0.22	0.11	-649.87	-1.16
NiRh	CHO	-653.00	0.49	0.11	-652.62	0.65
	slab	-634.18			-657.41	0.00
NiIr	COOH	-661.26	0.64	0.17	-660.79	0.03
NiIr	CO	-651.40	0.24	0.09	-651.25	-1.28
NiIr	CHO	-654.00	0.46	0.11	-653.64	1.01
	slab	-631.98			-655.22	0.00
CuRu	COOH	-659.54	0.63	0.14	-659.04	-0.43
CuRu	CO	-649.25	0.22	0.10	-649.13	-0.91
CuRu	CHO	-652.71	0.45	0.10	-652.35	0.18
	slab	-629.93			-653.16	0.00
ZnRu	COOH	-657.95	0.65	0.14	-657.44	-0.88
ZnRu	CO	-647.09	0.23	0.09	-646.96	-0.33
ZnRu	CHO	-650.56	0.50	0.09	-650.15	0.20
	slab	-629.01			-652.24	0.00
ZnRh	COOH	-655.99	0.64	0.14	-655.49	0.16
ZnRh	CO	-645.73	0.24	0.08	-645.57	-0.91
ZnRh	CHO	-648.56	0.48	0.11	-648.19	0.78
	slab	-629.91			-653.15	0.00
ZnIr	COOH	-657.19	0.63	0.17	-656.72	-0.18
ZnIr	CO	-647.12	0.24	0.08	-646.95	-1.05
ZnIr	CHO	-649.77	0.48	0.13	-649.41	0.94
	slab	-627.68			-650.91	0.00
ZnPt	COOH	-655.04	0.65	0.13	-654.51	-0.20
ZnPt	CO	-644.29	0.23	0.09	-644.15	-0.45
ZnPt	CHO	-647.55	0.50	0.10	-647.14	0.41
	slab	-631.73			-654.97	0.00
GaRu	COOH	-658.48	0.64	0.17	-658.01	0.35

GaRu	CO	-646.87	0.19	0.13	-646.81	0.38
GaRu	CHO	-646.89	0.19	0.15	-646.85	3.37
	slab	-629.05			-652.28	0.00
GaPd	COOH	-655.36	0.63	0.15	-654.88	0.80
GaPd	CO	-644.75	0.21	0.11	-644.66	-0.59
GaPd	CHO	-647.77	0.47	0.09	-647.39	0.67
	slab	-632.97			-656.20	0.00
GaOs	COOH	-660.50	0.63	0.17	-660.04	-0.44
GaOs	CO	-650.53	0.24	0.08	-650.38	-1.16
GaOs	CHO	-654.06	0.46	0.10	-653.69	0.08
	slab	-629.98			-653.21	0.00
GaPt	COOH	-656.38	0.66	0.14	-655.86	0.75
GaPt	CO	-645.80	0.23	0.11	-645.68	-0.64
GaPt	CHO	-648.64	0.49	0.12	-648.27	0.81
	slab	-637.14			-660.38	0.00
NbRh	COOH	-664.98	0.62	0.20	-664.56	-0.78
NbRh	CO	-652.81	0.15	0.14	-652.80	0.94
NbRh	CHO	-657.45	0.47	0.16	-657.14	-0.94
	slab	-632.43			-655.66	0.00
NbAg	COOH	-659.42	0.65	0.15	-658.92	0.15
NbAg	CO	-648.44	0.21	0.11	-648.35	-0.25
NbAg	CHO	-651.86	0.46	0.11	-651.50	0.25
	slab	-635.11			-658.35	0.00
RuRh	COOH	-662.39	0.63	0.12	-661.88	-0.14
RuRh	CO	-651.83	0.22	0.12	-651.74	-0.67
RuRh	CHO	-655.11	0.48	0.12	-654.75	0.39
	slab	-632.99			-656.23	0.00
RuPd	COOH	-660.26	0.63	0.12	-659.75	-0.12
RuPd	CO	-650.33	0.23	0.11	-650.20	-1.28
RuPd	CHO	-653.83	0.24	0.08	-653.67	-0.07
	slab	-631.88			-655.11	0.00
RhPd	COOH	-657.99	0.63	0.17	-657.52	0.99
RhPd	CO	-648.78	0.22	0.11	-648.67	-1.96
RhPd	CHO	-651.75	0.49	0.11	-651.38	0.69
	slab	-640.35			-663.59	0.00
RhTa	COOH	-668.23	0.58	0.17	-667.82	-0.83
RhTa	CO	-655.32	0.21	0.10	-655.21	1.79
RhTa	CHO	-659.44	0.39	0.12	-659.17	-0.56
	slab	-632.88			-656.12	0.00
RhPt	COOH	-660.03	0.63	0.17	-659.57	-0.06
RhPt	CO	-649.83	0.24	0.08	-649.67	-0.92
RhPt	CHO	-652.75	0.48	0.08	-652.35	0.72
	slab	-636.29			-659.52	0.00
PdTa	COOH	-665.70	0.58	0.17	-665.28	-2.36
PdTa	CO	-652.84	0.21	0.11	-652.74	1.72
PdTa	CHO	-656.97	0.39	0.12	-656.71	-0.57
	slab	-634.02			-657.25	0.00
IrPt	COOH	-660.09	0.65	0.15	-659.59	1.06

IrPt	CO	-651.14	0.23	0.09	-651.00	-2.23
IrPt	CHO	-653.95	0.49	0.11	-653.56	0.84
	slab	-634.71			-657.95	0.00
CoCo	COOH	-661.61	0.62	0.19	-661.18	0.17
CoCo	CO	-651.79	0.10	0.05	-651.74	-1.38
CoCo	CHO	-654.45	0.47	0.14	-654.11	1.02
	slab	-634.02			-657.26	0.00
RhRh	COOH	-660.56	0.65	0.16	-660.08	0.58
RhRh	CO	-651.13	0.23	0.08	-650.98	-1.72
RhRh	CHO	-653.11	0.50	0.09	-652.71	1.67
	slab	-636.38			-659.62	0.00
IrIr	COOH	-663.08	0.65	0.16	-662.59	0.42
IrIr	CO	-653.51	0.24	0.07	-653.34	-1.56
IrIr	CHO	-655.88	0.48	0.09	-655.49	1.25

Table S11. The free energy (ΔG/eV) of electrocatalytic CO_2 reduction on TM1/TM2–N@Gra to generate CH_4 (Path1: CO_2-*COOH-*CO-*CHO-*CH_2O-*CH_3O-*O+CH_4-*OH-*H_2O).

metal	molecules	E0	E_ZPE	T*S	ΔE /eV	ΔG/eV
	slab	-632.18	-632.17		-655.41	0.00
AlRh	COOH	-660.88	0.65	0.14	-660.36	-1.55
AlRh	CO	-649.32	0.22	0.11	-649.21	0.33
AlRh	CHO	-653.46	0.50	0.09	-653.05	-0.44
AlRh	CH2O	-656.81	0.79	0.12	-656.14	0.31
AlRh	CH3O	-661.09	1.11	0.20	-660.17	-0.63
AlRh	O	-640.54	0.09	0.03	-640.48	-0.95
AlRh	OH	-644.98	0.39	0.06	-644.65	-0.77
AlRh	H2O		-14.25		-646.43	1.63
	slab	-631.92			-655.16	0.00
AlPt	COOH	-659.26	0.67	0.13	-658.72	-0.17
AlPt	CO	-647.66	0.21	0.08	-647.52	0.38
AlPt	CHO	-651.74	0.52	0.07	-651.29	-0.36
AlPt	CH2O	-656.15	0.81	0.07	-655.41	-0.73
AlPt	CH3O	-659.72	1.08	0.07	-658.72	0.10
AlPt	O	-639.44	0.09	0.03	-639.38	-1.30
AlPt	OH	-643.31	0.39	0.05	-642.97	-0.19
AlPt	H2O		-14.25		-646.17	0.20
	slab	-630.93			-654.17	0.00
AlPd	COOH	-658.13	0.63	0.11	-657.62	-0.05
AlPd	CO	-646.86	0.22	0.07	-646.72	0.08
AlPd	CHO	-650.50	0.48	0.06	-650.08	0.03
AlPd	CH2O	-655.10	0.81	0.10	-654.39	-0.90
AlPd	CH3O	-658.97	1.11	0.19	-658.05	-0.26
AlPd	O	-638.48	0.09	0.03	-638.42	-1.01
AlPd	OH	-642.93	0.36	0.08	-642.65	-0.83

AlPd	H2O		-14.25		-645.18	0.87
	slab	-638.27			-661.50	0.00
VRu	COOH	-666.12	0.64	0.10	-665.59	-0.68
VRu	CO	-655.12	0.23	0.09	-654.98	-0.21
VRu	CHO	-658.70	0.48	0.12	-658.34	0.04
VRu	CH2O	-663.01	0.80	0.13	-662.33	-0.59
VRu	CH3O	-666.48	1.11	0.12	-665.49	0.24
VRu	O	-646.46	0.08	0.06	-646.44	-1.59
VRu	OH	-650.26	0.35	0.11	-650.02	-0.18
VRu	H2O		-14.25		-652.52	0.90
	slab	-634.50			-657.73	0.00
VPd	COOH	-661.69	0.64	0.15	-661.21	-0.08
VPd	CO	-650.51	0.20	0.11	-650.42	-0.03
VPd	CHO	-654.41	0.49	0.10	-654.02	-0.20
VPd	CH2O	-659.04	0.82	0.11	-658.34	-0.92
VPd	CH3O	-663.26	1.11	0.13	-662.28	-0.54
VPd	O	-643.57	0.09	0.04	-643.52	-1.88
VPd	OH	-647.07	0.34	0.09	-646.82	0.10
VPd	H2O		-14.25		-648.75	1.48
	slab	-636.26			-659.50	0.00
CrNi	COOH	-663.14	0.64	0.11	-662.61	0.29
CrNi	CO	-652.40	0.22	0.10	-652.28	-0.49
CrNi	CHO	-655.73	0.50	0.12	-655.35	0.33
CrNi	CH2O	-659.96	0.81	0.12	-659.27	-0.52
CrNi	CH3O	-663.95	1.09	0.22	-663.08	-0.41
CrNi	O	-644.12	0.08	0.05	-644.09	-1.65
CrNi	OH	-647.78	0.36	0.08	-647.50	-0.01
CrNi	H2O		-14.25		-650.51	0.39
	slab	-636.98			-660.22	0.00
CrRh	COOH	-663.67	0.61	0.10	-663.17	0.45
CrRh	CO	-652.91	0.22	0.10	-652.80	-0.45
CrRh	CHO	-656.77	0.49	0.10	-656.38	-0.18
CrRh	CH2O	-661.04	0.80	0.11	-660.35	-0.57
CrRh	CH3O	-664.62	1.08	0.11	-663.65	0.09
CrRh	O	-644.69	0.08	0.06	-644.66	-1.65
CrRh	OH	-648.48	0.35	0.08	-648.21	-0.14
CrRh	H2O		-14.25		-651.23	0.38
	slab	-636.54			-659.78	0.00
MnRh	COOH	-664.14	0.64	0.16	-663.65	-0.47
MnRh	CO	-653.60	0.22	0.09	-653.46	-0.63
MnRh	CHO	-656.82	0.50	0.10	-656.43	0.43
MnRh	CH2O	-660.58	0.80	0.12	-659.90	-0.07

MnRh	CH3O	-663.93	1.09	0.20	-663.04	0.26
MnRh	O	-643.42	0.08	0.05	-643.39	-0.99
MnRh	OH	-647.39	0.28	0.01	-647.13	-0.34
MnRh	H2O		-14.25		-650.79	-0.27
	slab	-637.80			-661.04	0.00
MnIr	COOH	-665.52	0.66	0.14	-665.00	-0.56
MnIr	CO	-654.89	0.21	0.13	-654.82	-0.63
MnIr	CHO	-658.18	0.52	0.10	-657.76	0.45
MnIr	CH2O	-661.65	0.82	0.07	-660.91	0.26
MnIr	CH3O	-664.98	1.09	0.20	-664.08	0.22
MnIr	O	-644.48	0.07	0.06	-644.46	-1.02
MnIr	OH	-648.82	0.35	0.08	-648.55	-0.68
MnIr	H2O		-14.25		-652.05	-0.11
	slab	-629.93			-653.16	0.00
ZnRu	COOH	-657.95	0.65	0.14	-657.44	-0.88
ZnRu	CO	-647.09	0.23	0.09	-646.96	-0.33
ZnRu	CHO	-650.56	0.50	0.09	-650.15	0.21
ZnRu	CH2O	-654.00	0.81	0.10	-653.29	0.26
ZnRu	CH3O	-657.08	1.07	0.18	-656.18	0.51
ZnRu	O	-637.22	0.08	0.04	-637.18	-1.63
ZnRu	OH	-641.39	0.39	0.05	-641.05	-0.47
ZnRu	H2O		-14.25		-644.18	0.27
	slab	-627.68			-650.91	0.00
ZnPt	COOH	-655.04	0.65	0.13	-654.51	-0.20
ZnPt	CO	-644.29	0.23	0.09	-644.15	-0.45
ZnPt	CHO	-647.55	0.50	0.10	-647.14	0.41
ZnPt	CH2O	-650.67	0.79	0.13	-650.02	0.52
ZnPt	CH3O	-654.89	1.10	0.19	-653.98	-0.56
ZnPt	O	-634.20	0.08	0.04	-634.16	-0.82
ZnPt	OH	-639.03	0.39	0.05	-638.69	-1.13
ZnPt	H2O		-14.25		-641.93	0.16
	slab	-632.43			-655.66	0.00
NbAg	COOH	-659.42	0.65	0.15	-658.92	0.15
NbAg	CO	-648.44	0.21	0.11	-648.35	-0.25
NbAg	CHO	-651.86	0.46	0.11	-651.50	0.25
NbAg	CH2O	-656.73	0.79	0.13	-656.07	-1.17
NbAg	CH3O	-660.80	1.11	0.09	-659.78	-0.31
NbAg	O	-641.67	0.08	0.05	-641.64	-2.50
NbAg	OH	-644.56	0.33	0.11	-644.34	0.70
NbAg	H2O		-14.25		-646.68	1.06
	slab	-635.11			-658.35	0.00
RuRh	COOH	-662.39	0.63	0.12	-661.88	-0.14

RuRh	CO	-651.83	0.22	0.12	-651.74	-0.67
RuRh	CHO	-655.11	0.48	0.12	-654.75	0.39
RuRh	CH2O	-658.44	0.80	0.15	-657.79	0.36
RuRh	CH3O	-662.37	1.10	0.19	-661.46	-0.27
RuRh	O	-642.10	0.08	0.05	-642.07	-1.25
RuRh	OH	-646.26	0.36	0.09	-645.99	-0.52
RuRh	H2O		-14.25		-649.36	0.03

Table S12. The free energy (ΔG/eV) of electrocatalytic CO_2 reduction on TM1/TM2–N@Gra to generate CH_4 (Path2: CO_2-*COOH-*CO-*CHO-*CHOH-*CH-*CH_2-*CH_3-*CH_4).

metal	molecules	E0	E_ZPE	T*S	ΔE /eV	ΔG/eV
	slab	-632.18	-632.17		-655.41	0.00
AlRh	COOH	-660.88	0.65	0.14	-660.36	-1.55
AlRh	CO	-649.32	0.22	0.11	-649.21	0.33
AlRh	CHO	-653.46	0.50	0.09	-653.05	-0.44
AlRh	CHOH	-655.35	0.75	0.17	-654.77	1.67
AlRh	CH	-644.29	0.36	0.05	-643.98	-0.03
AlRh	CH2	-649.02	0.66	0.06	-648.43	-1.05
AlRh	CH3	-653.52	0.96	0.10	-652.67	-0.84
AlRh	CH4	-656.35	1.21	0.18	-655.33	0.74
	slab	-633.17			-656.40	0.00
AlIr	COOH	-662.20	0.64	0.10	-661.66	-1.86
AlIr	CO	-650.82	0.22	0.06	-650.66	0.18
AlIr	CHO	-654.81	0.52	0.08	-654.37	-0.31
AlIr	CHOH	-656.96	0.78	0.14	-656.32	1.45
AlIr	CH	-645.67	0.37	0.04	-645.33	0.17
AlIr	CH2	-650.30	0.66	0.06	-649.71	-0.97
AlIr	CH3	-654.65	0.95	0.11	-653.81	-0.70
AlIr	CH4	-657.35	1.21	0.23	-656.37	0.84
	slab	-631.92			-655.16	0.00
AlPt	COOH	-659.26	0.67	0.13	-658.72	-0.17
AlPt	CO	-647.66	0.21	0.08	-647.52	0.38
AlPt	CHO	-651.74	0.52	0.07	-651.29	-0.36
AlPt	CHOH	-653.84	0.78	0.10	-653.16	1.53
AlPt	CH	-642.82	0.35	0.05	-642.52	-0.18
AlPt	CH2	-648.27	0.68	0.05	-647.65	-1.73
AlPt	CH3	-652.05	0.95	0.08	-651.19	-0.14
AlPt	CH4	-656.08	1.20	0.13	-655.01	-0.42
	slab	-630.93			-654.17	0.00
AlPd	COOH	-658.13	0.63	0.11	-657.62	-0.05
AlPd	CO	-646.86	0.22	0.07	-646.72	0.08
AlPd	CHO	-650.50	0.48	0.06	-650.08	0.03
AlPd	CHOH	-652.96	0.77	0.13	-652.32	1.17
AlPd	CH	-641.72	0.34	0.06	-641.43	0.06
AlPd	CH2	-647.24	0.67	0.06	-646.62	-1.79
AlPd	CH3	-651.10	0.93	0.13	-650.29	-0.27

AlPd	CH4	-655.11	1.23	0.18	-654.05	-0.36
	slab	-638.27			-661.50	0.00
VRu	COOH	-666.12	0.64	0.10	-665.59	-0.68
VRu	CO	-655.12	0.23	0.09	-654.98	-0.21
VRu	CHO	-658.70	0.48	0.12	-658.34	0.04
VRu	CHOH	-661.08	0.80	0.15	-660.43	1.31
VRu	CH	-650.79	0.36	0.05	-650.48	-0.86
VRu	CH2	-655.14	0.66	0.07	-654.56	-0.68
VRu	CH3	-658.87	0.93	0.09	-658.02	-0.06
VRu	CH4	-662.44	1.20	0.14	-661.38	0.04
	slab	-634.50			-657.73	0.00
VPd	COOH	-661.69	0.64	0.15	-661.21	-0.08
VPd	CO	-650.51	0.20	0.11	-650.42	-0.03
VPd	CHO	-654.41	0.49	0.10	-654.02	-0.20
VPd	CHOH	-657.62	0.79	0.12	-656.95	0.47
VPd	CH	-646.26	0.36	0.05	-645.95	0.18
VPd	CH2	-650.93	0.65	0.07	-650.35	-1.00
VPd	CH3	-654.71	0.95	0.09	-653.85	-0.10
VPd	CH4	-658.66	1.22	0.14	-657.58	-0.33
	slab	-636.26			-659.50	0.00
CrNi	COOH	-663.14	0.64	0.11	-662.61	0.29
CrNi	CO	-652.40	0.22	0.10	-652.28	-0.49
CrNi	CHO	-655.73	0.50	0.12	-655.35	0.33
CrNi	CHOH	-659.34	0.81	0.11	-658.64	0.11
CrNi	CH	-647.90	0.25	0.01	-647.66	0.16
CrNi	CH2	-652.57	0.66	0.06	-651.97	-0.91
CrNi	CH3	-656.48	0.94	0.13	-655.67	-0.30
CrNi	CH4	-660.46	1.21	0.25	-659.49	-0.42
	slab	-636.98			-660.22	0.00
CrRh	COOH	-663.67	0.61	0.10	-663.17	0.45
CrRh	CO	-652.91	0.22	0.10	-652.80	-0.45
CrRh	CHO	-656.77	0.49	0.10	-656.38	-0.18
CrRh	CHOH	-660.27	0.79	0.09	-659.58	0.20
CrRh	CH	-649.15	0.36	0.05	-648.85	-0.08
CrRh	CH2	-653.64	0.67	0.05	-653.03	-0.78
CrRh	CH3	-657.41	0.93	0.09	-656.57	-0.14
CrRh	CH4	-661.19	1.21	0.17	-660.16	-0.19
	slab	-636.54			-659.78	0.00
MnRh	COOH	-664.14	0.64	0.16	-663.65	-0.47
MnRh	CO	-653.60	0.22	0.09	-653.46	-0.63
MnRh	CHO	-656.82	0.50	0.10	-656.43	0.43
MnRh	CHOH	-660.20	0.80	0.15	-659.55	0.28
MnRh	CH	-648.74	0.37	0.05	-648.42	0.31
MnRh	CH2	-653.46	0.67	0.06	-652.85	-1.03
MnRh	CH3	-657.28	0.95	0.07	-656.40	-0.15
MnRh	CH4	-660.73	1.20	0.19	-659.72	0.08
	slab	-637.80			-661.04	0.00

MnIr	COOH	-665.52	0.66	0.14	-665.00	-0.56
MnIr	CO	-654.89	0.21	0.13	-654.82	-0.63
MnIr	CHO	-658.18	0.52	0.10	-657.76	0.45
MnIr	CHOH	-661.56	0.80	0.13	-660.88	0.28
MnIr	CH	-650.17	0.37	0.05	-649.84	0.21
MnIr	CH2	-654.84	0.68	0.05	-654.21	-0.96
MnIr	CH3	-658.56	0.96	0.06	-657.66	-0.05
MnIr	CH4	-662.00	1.21	0.13	-660.93	0.12
	slab	-629.93			-653.16	0.00
ZnRu	COOH	-657.95	0.65	0.14	-657.44	-0.88
ZnRu	CO	-647.09	0.23	0.09	-646.96	-0.33
ZnRu	CHO	-650.56	0.50	0.09	-650.15	0.21
ZnRu	CHOH	-653.00	0.80	0.12	-652.32	1.23
ZnRu	CH	-642.08	0.36	0.06	-641.78	-0.28
ZnRu	CH2	-646.59	0.67	0.06	-645.97	-0.79
ZnRu	CH3	-650.60	0.95	0.10	-649.75	-0.38
ZnRu	CH4	-654.08	1.23	0.27	-653.13	0.02
	slab	-627.68			-650.91	0.00
ZnPt	COOH	-655.04	0.65	0.13	-654.51	-0.20
ZnPt	CO	-644.29	0.23	0.09	-644.15	-0.45
ZnPt	CHO	-647.55	0.50	0.10	-647.14	0.41
ZnPt	CHOH	-650.35	0.82	0.12	-649.65	0.89
ZnPt	CH	-638.32	0.35	0.07	-638.04	0.79
ZnPt	CH2	-643.26	0.66	0.07	-642.68	-1.24
ZnPt	CH3	-647.32	0.93	0.05	-646.44	-0.36
ZnPt	CH4	-652.07	1.17	0.07	-650.97	-1.14
	slab	-632.43			-655.66	0.00
NbAg	COOH	-659.42	0.65	0.15	-658.92	0.15
NbAg	CO	-648.44	0.21	0.11	-648.35	-0.25
NbAg	CHO	-651.86	0.46	0.11	-651.50	0.25
NbAg	CHOH	-655.20	0.78	0.14	-654.57	0.34
NbAg	CH	-644.23	0.35	0.06	-643.95	-0.20
NbAg	CH2	-648.67	0.60	0.11	-648.18	-0.83
NbAg	CH3	-652.99	0.92	0.10	-652.16	-0.58
NbAg	CH4	-656.66	1.22	0.20	-655.64	-0.08
	slab	-635.11			-658.35	0.00
RuRh	COOH	-662.39	0.63	0.12	-661.88	-0.14
RuRh	CO	-651.83	0.22	0.12	-651.74	-0.67
RuRh	CHO	-655.11	0.48	0.12	-654.75	0.39
RuRh	CHOH	-658.21	0.80	0.14	-657.56	0.59
RuRh	CH	-647.51	0.38	0.04	-647.18	-0.44
RuRh	CH2	-651.77	0.67	0.07	-651.17	-0.58
RuRh	CH3	-655.80	0.94	0.10	-654.97	-0.40
RuRh	CH4	-659.26	1.21	0.16	-658.22	0.15

Table S13. The free energy (ΔG/eV) of electrocatalytic CO_2 reduction on TM1/TM2–N@Gra to generate CH_4 (Path3: CO_2-*COOH-*CO - * CHO -*CH_2O-*CH_2OH-*CH_2-*CH_3-*CH_4).

metal	molecules	E0	E_ZPE	T*S	ΔE /eV	ΔG/eV
	slab	-632.18	-632.17		-655.41	0.00
AlRh	COOH	-660.88	0.65	0.14	-660.36	-1.55
AlRh	CO	-649.32	0.22	0.11	-649.21	0.33
AlRh	CHO	-653.46	0.50	0.09	-653.05	-0.44
AlRh	CH2O	-656.81	0.79	0.12	-656.14	0.31
AlRh	CH2OH	-660.39	1.10	0.13	-659.43	0.11
AlRh	CH2	-649.02	0.66	0.06	-648.43	0.18
AlRh	CH3	-653.52	0.96	0.10	-652.67	-0.84
AlRh	CH4	-656.35	1.21	0.18	-655.33	0.74
	slab	-631.92			-655.16	0.00
AlPt	COOH	-659.26	0.67	0.13	-658.72	-0.17
AlPt	CO	-647.66	0.21	0.08	-647.52	0.38
AlPt	CHO	-651.74	0.52	0.07	-651.29	-0.36
AlPt	CH2O	-656.15	0.81	0.07	-655.41	-0.73
AlPt	CH2OH	-659.05	1.13	0.13	-658.05	0.76
AlPt	CH2	-648.27	0.68	0.05	-647.65	-0.42
AlPt	CH3	-652.05	0.95	0.08	-651.19	-0.14
AlPt	CH4	-656.08	1.20	0.13	-655.01	-0.42
	slab	-630.93			-654.17	0.00
AlPd	COOH	-658.13	0.63	0.11	-657.62	-0.05
AlPd	CO	-646.86	0.22	0.07	-646.72	0.08
AlPd	CHO	-650.50	0.48	0.06	-650.08	0.03
AlPd	CH2O	-655.10	0.81	0.10	-654.39	-0.90
AlPd	CH2OH	-657.82	1.11	0.14	-656.85	0.94
AlPd	CH2	-647.24	0.67	0.06	-646.62	-0.59
AlPd	CH3	-651.10	0.93	0.13	-650.29	-0.27
AlPd	CH4	-655.11	1.23	0.18	-654.05	-0.36
	slab	-638.27			-661.50	0.00
VRu	COOH	-666.12	0.64	0.10	-665.59	-0.68
VRu	CO	-655.12	0.23	0.09	-654.98	-0.21
VRu	CHO	-658.70	0.48	0.12	-658.34	0.04
VRu	CH2O	-663.01	0.80	0.13	-662.33	-0.59
VRu	CH2OH	-665.70	1.13	0.13	-664.70	1.03
VRu	CH2	-655.14	0.66	0.07	-654.56	-0.68
VRu	CH3	-658.87	0.93	0.09	-658.02	-0.06
VRu	CH4	-662.44	1.20	0.14	-661.38	0.04
	slab	-634.50			-657.73	0.00
VPd	COOH	-661.69	0.64	0.15	-661.21	-0.08
VPd	CO	-650.51	0.20	0.11	-650.42	-0.03
VPd	CHO	-654.41	0.49	0.10	-654.02	-0.20
VPd	CH2O	-659.04	0.82	0.11	-658.34	-0.92
VPd	CH2OH	-661.05	1.08	0.11	-660.08	1.66
VPd	CH2	-650.93	0.65	0.07	-650.35	-1.08
VPd	CH3	-654.71	0.95	0.09	-653.85	-0.10
VPd	CH4	-658.66	1.22	0.14	-657.58	-0.33
	slab	-636.26			-659.50	0.00

CrNi	COOH	-663.14	0.64	0.11	-662.61	0.29
CrNi	CO	-652.40	0.22	0.10	-652.28	-0.49
CrNi	CHO	-655.73	0.50	0.12	-655.35	0.33
CrNi	CH2O	-659.96	0.81	0.12	-659.27	-0.52
CrNi	CH2OH	-662.91	1.08	0.15	-661.98	0.69
CrNi	CH2	-652.57	0.66	0.06	-651.97	-0.81
CrNi	CH3	-656.48	0.94	0.13	-655.67	-0.30
CrNi	CH4	-660.46	1.21	0.25	-659.49	-0.42
	slab	-636.98			-660.22	0.00
CrRh	COOH	-663.67	0.61	0.10	-663.17	0.45
CrRh	CO	-652.91	0.22	0.10	-652.80	-0.45
CrRh	CHO	-656.77	0.49	0.10	-656.38	-0.18
CrRh	CH2O	-661.04	0.80	0.11	-660.35	-0.57
CrRh	CH2OH	-663.80	1.08	0.15	-662.87	0.88
CrRh	CH2	-653.64	0.67	0.05	-653.03	-0.98
CrRh	CH3	-657.41	0.93	0.09	-656.57	-0.14
CrRh	CH4	-661.19	1.21	0.17	-660.16	-0.19
	slab	-636.54			-659.78	0.00
MnRh	COOH	-664.14	0.64	0.16	-663.65	-0.47
MnRh	CO	-653.60	0.22	0.09	-653.46	-0.63
MnRh	CHO	-656.82	0.50	0.10	-656.43	0.43
MnRh	CH2O	-660.58	0.80	0.12	-659.90	-0.07
MnRh	CH2OH	-663.59	1.08	0.13	-662.64	0.66
MnRh	CH2	-653.46	0.67	0.06	-652.85	-1.03
MnRh	CH3	-657.28	0.95	0.07	-656.40	-0.15
MnRh	CH4	-660.73	1.20	0.19	-659.72	0.08
	slab	-637.80			-661.04	0.00
MnIr	COOH	-665.52	0.66	0.14	-665.00	-0.56
MnIr	CO	-654.89	0.21	0.13	-654.82	-0.63
MnIr	CHO	-658.18	0.52	0.10	-657.76	0.45
MnIr	CH2O	-661.65	0.82	0.07	-660.91	0.26
MnIr	CH2OH	-664.95	1.09	0.14	-664.00	0.31
MnIr	CH2	-654.84	0.68	0.05	-654.21	-1.03
MnIr	CH3	-658.56	0.96	0.06	-657.66	-0.05
MnIr	CH4	-662.00	1.21	0.13	-660.93	0.12
	slab	-629.93			-653.16	0.00
ZnRu	COOH	-657.95	0.65	0.14	-657.44	-0.88
ZnRu	CO	-647.09	0.23	0.09	-646.96	-0.33
ZnRu	CHO	-650.56	0.50	0.09	-650.15	0.21
ZnRu	CH2O	-654.00	0.81	0.10	-653.29	0.26
ZnRu	CH2OH	-657.54	1.10	0.11	-656.55	0.15
ZnRu	CH2	-646.59	0.67	0.06	-645.97	-0.25
ZnRu	CH3	-650.60	0.95	0.10	-649.75	-0.38
ZnRu	CH4	-654.08	1.23	0.27	-653.13	0.02
	slab	-627.68			-650.91	0.00
ZnPt	COOH	-655.04	0.65	0.13	-654.51	-0.20
ZnPt	CO	-644.29	0.23	0.09	-644.15	-0.45

ZnPt	CHO	-647.55	0.50	0.10	-647.14	0.41
ZnPt	CH2O	-650.67	0.79	0.13	-650.02	0.52
ZnPt	CH2OH	-654.91	1.13	0.14	-653.92	-0.50
ZnPt	CH2	-643.26	0.66	0.07	-642.68	0.42
ZnPt	CH3	-647.32	0.93	0.05	-646.44	-0.36
ZnPt	CH4	-652.07	1.17	0.07	-650.97	-1.14
	slab	-632.43			-655.66	0.00
NbAg	COOH	-659.42	0.65	0.15	-658.92	0.15
NbAg	CO	-648.44	0.21	0.11	-648.35	-0.25
NbAg	CHO	-651.86	0.46	0.11	-651.50	0.25
NbAg	CH2O	-656.73	0.79	0.13	-656.07	-1.17
NbAg	CH2OH	-659.54	1.09	0.12	-658.57	0.90
NbAg	CH2	-648.67	0.60	0.11	-648.18	-0.43
NbAg	CH3	-652.99	0.92	0.10	-652.16	-0.58
NbAg	CH4	-656.66	1.22	0.20	-655.64	-0.08
	slab	-635.11			-658.35	0.00
RuRh	COOH	-662.39	0.63	0.12	-661.88	-0.14
RuRh	CO	-651.83	0.22	0.12	-651.74	-0.67
RuRh	CHO	-655.11	0.48	0.12	-654.75	0.39
RuRh	CH2O	-658.44	0.80	0.15	-657.79	0.36
RuRh	CH2OH	-662.36	1.10	0.13	-661.39	-0.20
RuRh	CH2	-651.77	0.67	0.07	-651.17	-0.60
RuRh	CH3	-655.80	0.94	0.10	-654.97	-0.40
RuRh	CH4	-659.26	1.21	0.16	-658.22	0.15

Table S14. The free energy (ΔG/eV) of electrocatalytic CO_2 reduction on TM1/TM2–N@Gra to generate CH_4 (Path4: CO_2-*HCOO-*HCOOH-*CHO-*CHOH-*CH-*CH_2-*CH_3 -*CH_4).

metal	molecules	E_0/eV	E_ZPE/eV	T*S	ΔE/eV	ΔG/eV
	COO	-631.92			-655.16	0.00
AlPt	HCOO	-659.62	0.64	0.17	-659.16	-0.60
	HCOOH	-663.18	0.93	0.16	-662.41	0.15
	CHO	-651.74	0.52	0.07	-651.29	0.30
	CH2O	-656.15	0.81	0.07	-655.41	-0.73
	CH3O	-659.72	1.08	0.07	-658.72	0.10
	O	-639.44	0.09	0.03	-639.38	-1.30
	OH	-643.31	0.39	0.05	-642.97	-0.19
	H2O		-14.25		-646.17	0.20
	COO	-630.93			-654.17	0.00
AlPd	HCOO	-658.77	0.62	0.15	-658.30	-0.73
	HCOOH	-662.20	0.96	0.13	-661.37	0.34
	HCOOH				-660.73	0.63
	COO	-634.50			-657.73	0.00
VPd	HCOO	-662.60	0.62	0.18	-662.15	-1.02
	HCOOH	-665.43	0.93	0.17	-664.67	0.88
	CHO	-654.41	0.49	0.10	-654.02	-0.17

	CHOH	-657.62	0.79	0.12	-656.95	0.47
	CH	-646.26	0.36	0.05	-645.95	0.18
	CH2	-650.93	0.65	0.07	-650.35	-1.00
	CH3	-654.71	0.95	0.09	-653.85	-0.10
	CH4	-658.66	1.22	0.14	-657.58	-0.33
	COO	-636.26			-659.50	0.00
CrNi	HCOO	-663.42	0.60	0.15	-662.97	-0.07
	HCOOH	-667.00	0.91	0.15	-666.24	0.13
	CHO	-655.73	0.50	0.12	-655.35	0.07
	CHOH	-659.34	0.81	0.11	-658.64	0.11
	CH	-647.90	0.25	0.01	-647.66	0.16
	CH2	-652.57	0.66	0.06	-651.97	-0.91
	CH3	-656.48	0.94	0.13	-655.67	-0.30
	CH4	-660.46	1.21	0.25	-659.49	-0.42
	COO	-636.98			-660.22	0.00
CrRh	HCOO	-664.18	0.62	0.18	-663.74	-0.12
	HCOOH	-667.42	0.92	0.20	-666.69	0.45
	CHO	-656.77	0.49	0.10	-656.38	-0.51
	CHOH	-660.27	0.79	0.09	-659.58	0.20
	CH	-649.15	0.36	0.05	-648.85	-0.08
	CH2	-653.64	0.67	0.05	-653.03	-0.78
	CH3	-657.41	0.93	0.09	-656.57	-0.14
	CH4	-661.19	1.21	0.17	-660.16	-0.19
	COO	-632.43			-655.66	0.00
NbAg	HCOO	-660.09	0.59	0.13	-659.62	-0.56
	HCOOH	-663.19	0.90	0.15	-662.44	0.58
	CHO	-651.86	0.46	0.11	-651.50	0.12
	CHOH	-655.20	0.78	0.14	-654.56	0.34
	CH	-644.23	0.35	0.06	-643.95	-0.20
	CH2	-648.67	0.60	0.11	-648.18	-0.83
	CH3	-652.99	0.92	0.10	-652.16	-0.58
	CH4	-656.66	1.22	0.20	-655.64	-0.08

Table S15. Adsorption energy of potential product on diatomic catalyst surface.

Metal	CO	HCOOH	CH3OH	CH4
AlRh	-1.87	-1.84	-1.16	-0.13
AlPd	-0.66	-1.47	-0.91	-0.13
AlPt	-0.47	-1.46	-0.86	-0.12
VRu	-1.58	-1.75	-0.87	-0.13
VPd	-0.74	-1.13	-0.34	-0.12
CrNi	-0.87	-0.94	-0.25	-0.15
CrRh	-0.66	-0.63	-0.24	-0.17
MnRh	-1.78	-1.23	-0.74	-0.15
MnIr	-1.82	-1.02	-0.28	-0.16
ZnRu	-1.89	-1.31	660.15	-0.11
ZnPt	-1.34	-0.40	-0.59	-0.35
NbAg	-0.74	-0.97	-0.76	-0.19

RuRh	-1.45	-0.30	-0.32	-0.11
AlIr	-2.38	-1.98		-0.14

Table S16. The free energy (ΔG/eV) of electrocatalytic CO_2 reduction on TM1/TM2–N@Gra to generate CH_3OH (Path1: CO_2-*COOH-*CO-*CHO-*CHOH-*CH_2OH -*CH_3OH).

metal	molecules	E_0/eV	E_ZPE/eV	T*S	ΔE/eV	ΔG/eV
		-632.18	-632.18		-655.41	0.00
AlRh	COOH	-660.88	0.65	0.14	-660.36	-1.55
AlRh	CO	-649.32	0.22	0.11	-649.21	0.33
AlRh	CHO	-653.46	0.50	0.09	-653.05	-0.44
AlRh	CHOH	-655.35	0.75	0.17	-654.77	1.67
AlRh	CH2OH	-660.39	1.10	0.13	-659.43	-1.25
AlRh	CH3OH	-663.56	1.45	0.21	-662.32	0.50
		-631.92			-655.16	0.00
AlPt	COOH	-659.26	0.67	0.13	-658.72	-0.17
AlPt	CO	-647.66	0.21	0.07	-647.52	0.38
AlPt	CHO	-651.74	0.52	0.07	-651.29	-0.36
AlPt	CHOH	-653.84	0.78	0.10	-653.16	1.53
AlPt	CH2OH	-659.05	1.13	0.13	-658.05	-1.49
AlPt	CH3OH	-663.00	1.44	0.22	-661.78	-0.33
		-630.93			-654.17	0.00
AlPd	COOH	-658.13	0.63	0.11	-657.62	-0.05
AlPd	CO	-646.86	0.22	0.07	-646.72	0.08
AlPd	CHO	-650.50	0.48	0.06	-650.08	0.03
AlPd	CHOH	-652.96	0.77	0.13	-652.32	1.17
AlPd	CH2OH	-657.82	1.11	0.14	-656.85	-1.13
AlPd	CH3OH	-662.07	1.43	0.23	-660.87	-0.62
		-638.27			-661.50	0.00
VRu	COOH	-666.12	0.64	0.10	-665.59	-0.68
VRu	CO	-655.12	0.23	0.09	-654.98	-0.21
VRu	CHO	-658.70	0.48	0.12	-658.34	0.04
VRu	CHOH	-661.08	0.80	0.15	-660.43	1.31
VRu	CH2OH	-665.70	1.13	0.13	-664.70	-0.87
VRu	CH3OH	-669.36	1.44	0.21	-668.13	-0.03
		-634.50			-657.73	0.00
VPd	COOH	-661.69	0.64	0.15	-661.21	-0.08
VPd	CO	-650.51	0.20	0.11	-650.42	-0.03
VPd	CHO	-654.41	0.49	0.10	-654.02	-0.20
VPd	CHOH	-657.62	0.79	0.12	-656.95	0.47
VPd	CH2OH	-661.05	1.08	0.11	-660.08	0.27
VPd	CH3OH	-665.06	1.40	0.24	-663.90	-0.42
		-636.26			-659.50	0.00
CrNi	COOH	-663.14	0.64	0.11	-662.61	0.29

CrNi	CO	-652.40	0.22	0.10	-652.28	-0.49
CrNi	CHO	-655.73	0.50	0.12	-655.35	0.33
CrNi	CHOH	-659.34	0.81	0.11	-658.64	0.11
CrNi	CH2OH	-662.91	1.08	0.15	-661.98	0.06
CrNi	CH3OH	-666.73	1.39	0.22	-665.56	-0.18
		-636.98			-660.22	0.00
CrRh	COOH	-663.67	0.61	0.10	-663.17	0.45
CrRh	CO	-652.91	0.22	0.10	-652.80	-0.45
CrRh	CHO	-656.77	0.49	0.10	-656.38	-0.18
CrRh	CHOH	-660.27	0.79	0.09	-659.58	0.20
CrRh	CH2OH	-663.80	1.08	0.15	-662.87	0.11
CrRh	CH3OH	-667.44	1.39	0.25	-666.31	-0.04
		-636.54			-659.78	0.00
MnRh	COOH	-664.14	0.64	0.16	-663.65	-0.47
MnRh	CO	-653.59	0.22	0.09	-653.46	-0.63
MnRh	CHO	-656.82	0.50	0.10	-656.43	0.43
MnRh	CHOH	-660.20	0.80	0.15	-659.55	0.28
MnRh	CH2OH	-663.59	1.08	0.13	-662.64	0.31
MnRh	CH3OH	-667.50	1.42	0.14	-666.23	-0.19
		-637.80			-661.04	0.00
MnIr	COOH	-665.52	0.66	0.14	-665.00	-0.56
MnIr	CO	-654.89	0.21	0.13	-654.82	-0.63
MnIr	CHO	-658.18	0.52	0.10	-657.76	0.45
MnIr	CHOH	-661.56	0.80	0.13	-660.88	0.28
MnIr	CH2OH	-664.95	1.09	0.14	-664.00	0.28
MnIr	CH3OH	-668.30	1.39	0.20	-667.11	0.29
		-629.93			-653.16	0.00
ZnRu	COOH	-657.95	0.65	0.14	-657.44	-0.88
ZnRu	CO	-647.09	0.23	0.09	-646.96	-0.33
ZnRu	CHO	-650.56	0.50	0.09	-650.15	0.20
ZnRu	CHOH	-653.00	0.80	0.12	-652.32	1.23
ZnRu	CH2OH	-657.54	1.10	0.11	-656.55	-0.82
ZnRu	CH3OH	-660.78	1.40	0.12	-659.50	0.45
		-627.68			-650.92	0.00
ZnPt	COOH	-655.04	0.65	0.13	-654.51	-0.20
ZnPt	CO	-644.29	0.23	0.09	-644.15	-0.45
ZnPt	CHO	-647.55	0.50	0.10	-647.14	0.41
ZnPt	CHOH	-650.35	0.82	0.12	-649.65	0.89
ZnPt	CH2OH	-654.91	1.13	0.14	-653.92	-0.87
ZnPt	CH3OH	-658.49	1.42	0.20	-657.27	0.06
		-632.43			-655.66	0.00
NbAg	COOH	-659.42	0.65	0.15	-658.92	0.15
NbAg	CO	-648.44	0.21	0.11	-648.35	-0.25
NbAg	CHO	-651.86	0.46	0.11	-651.50	0.25
NbAg	CHOH	-655.20	0.78	0.14	-654.57	0.34
NbAg	CH2OH	-659.54	1.09	0.12	-658.57	-0.60
NbAg	CH3OH	-663.41	1.44	0.21	-662.18	-0.21

		-635.11			-658.35	0.00
RuRh	COOH	-662.39	0.63	0.12	-661.89	-0.14
RuRh	CO	-651.83	0.22	0.12	-651.74	-0.67
RuRh	CHO	-655.11	0.48	0.12	-654.75	0.39
RuRh	CHOH	-658.21	0.80	0.14	-657.56	0.59
RuRh	CH2OH	-662.36	1.10	0.13	-661.39	-0.43
RuRh	CH3OH	-665.65	1.38	0.20	-664.47	0.31

Table S17. The free energy (ΔG/eV) of electrocatalytic CO_2 reduction on TM1/TM2–N@Gra to generate CH_3OH (Path2: CO_2-*COOH-*CO-*CHO-*CH_2-*CH_2OH -*CH_3OH).

metal	molecules	E_0/eV	E_ZPE/eV	T*S	ΔE/eV	ΔG/eV
		-632.18	-632.18		-655.41	0.00
AlRh	COOH	-660.88	0.65	0.14	-660.36	-1.55
AlRh	CO	-649.32	0.22	0.11	-649.21	0.33
AlRh	CHO	-653.46	0.50	0.09	-653.05	-0.44
AlRh	CH2O	-656.81	0.79	0.12	-656.14	0.31
AlRh	CH2OH	-660.39	1.10	0.13	-659.43	0.11
AlRh	CH3OH	-663.56	1.45	0.21	-662.32	0.50
		-631.92			-655.16	0.00
AlPt	COOH	-659.26	0.67	0.13	-658.72	-0.17
AlPt	CO	-647.66	0.21	0.07	-647.52	0.38
AlPt	CHO	-651.74	0.52	0.07	-651.29	-0.36
AlPt	CH2O	-656.15	0.81	0.07	-655.41	-0.73
AlPt	CH2OH	-659.05	1.13	0.13	-658.05	0.76
AlPt	CH3OH	-663.00	1.44	0.22	-661.78	-0.33
		-630.93			-654.17	0.00
AlPd	COOH	-658.13	0.63	0.11	-657.62	-0.05
AlPd	CO	-646.86	0.22	0.07	-646.72	0.08
AlPd	CHO	-650.50	0.48	0.06	-650.08	0.03
AlPd	CH2O	-655.10	0.81	0.10	-654.39	-0.90
AlPd	CH2OH	-657.82	1.11	0.14	-656.85	0.94
AlPd	CH3OH	-662.07	1.43	0.23	-660.87	-0.62
		-638.27			-661.50	0.00
VRu	COOH	-666.12	0.64	0.10	-665.59	-0.68
VRu	CO	-655.12	0.23	0.09	-654.98	-0.21
VRu	CHO	-658.70	0.48	0.12	-658.34	0.04
VRu	CH2O	-663.01	0.80	0.13	-662.33	-0.59
VRu	CH2OH	-665.70	1.13	0.13	-664.70	1.03
VRu	CH3OH	-669.36	1.44	0.21	-668.13	-0.03
		-634.50			-657.73	0.00
VPd	COOH	-661.69	0.64	0.15	-661.21	-0.08
VPd	CO	-650.51	0.20	0.11	-650.42	-0.03
VPd	CHO	-654.41	0.49	0.10	-654.02	-0.20
VPd	CH2O	-659.04	0.82	0.11	-658.34	-0.92
VPd	CH2OH	-661.05	1.08	0.11	-660.08	1.66
VPd	CH3OH	-665.06	1.40	0.24	-663.90	-0.42

		-636.26			-659.50	0.00
CrNi	COOH	-663.14	0.64	0.11	-662.61	0.29
CrNi	CO	-652.40	0.22	0.10	-652.28	-0.49
CrNi	CHO	-655.73	0.50	0.12	-655.35	0.33
CrNi	CH2O	-659.96	0.81	0.12	-659.27	-0.52
CrNi	CH2OH	-662.91	1.08	0.15	-661.98	0.69
CrNi	CH3OH	-666.73	1.39	0.22	-665.56	-0.18
		-636.98			-660.22	0.00
CrRh	COOH	-663.67	0.61	0.10	-663.17	0.45
CrRh	CO	-652.91	0.22	0.10	-652.80	-0.45
CrRh	CHO	-656.77	0.49	0.10	-656.38	-0.18
CrRh	CH2O	-661.04	0.80	0.11	-660.35	-0.57
CrRh	CH2OH	-663.80	1.08	0.15	-662.87	0.88
CrRh	CH3OH	-667.44	1.39	0.25	-666.31	-0.04
		-636.54			-659.78	0.00
MnRh	COOH	-664.14	0.64	0.16	-663.65	-0.47
MnRh	CO	-653.59	0.22	0.09	-653.46	-0.63
MnRh	CHO	-656.82	0.50	0.10	-656.43	0.43
MnRh	CH2O	-660.58	0.80	0.12	-659.90	-0.07
MnRh	CH2OH	-663.59	1.08	0.13	-662.64	0.66
MnRh	CH3OH	-667.50	1.42	0.14	-666.23	-0.19
		-637.80			-661.04	0.00
MnIr	COOH	-665.52	0.66	0.14	-665.00	-0.56
MnIr	CO	-654.89	0.21	0.13	-654.82	-0.63
MnIr	CHO	-658.18	0.52	0.10	-657.76	0.45
MnIr	CH2O	-661.65	0.82	0.07	-660.91	0.26
MnIr	CH2OH	-664.95	1.09	0.14	-664.00	0.31
MnIr	CH3OH	-668.30	1.39	0.20	-667.11	0.29
		-629.93			-653.16	0.00
ZnRu	COOH	-657.95	0.65	0.14	-657.44	-0.88
ZnRu	CO	-647.09	0.23	0.09	-646.96	-0.33
ZnRu	CHO	-650.56	0.50	0.09	-650.15	0.20
ZnRu	CH2O	-654.00	0.81	0.10	-653.30	0.26
ZnRu	CH2OH	-657.54	1.10	0.11	-656.55	0.15
ZnRu	CH3OH	-660.78	1.40	0.12	-659.50	0.45
		-627.68			-650.92	0.00
ZnPt	COOH	-655.04	0.65	0.13	-654.51	-0.20
ZnPt	CO	-644.29	0.23	0.09	-644.15	-0.45
ZnPt	CHO	-647.55	0.50	0.10	-647.14	0.41
ZnPt	CH2O	-650.67	0.79	0.13	-650.02	0.52
ZnPt	CH2OH	-654.91	1.13	0.14	-653.92	-0.50
ZnPt	CH3OH	-658.49	1.42	0.20	-657.27	0.06
		-632.43			-655.66	0.00
NbAg	COOH	-659.42	0.65	0.15	-658.92	0.15
NbAg	CO	-648.44	0.21	0.11	-648.35	-0.25
NbAg	CHO	-651.86	0.46	0.11	-651.50	0.25
NbAg	CH2O	-656.73	0.79	0.13	-656.07	-1.17

NbAg	CH2OH	-659.54	1.09	0.12	-658.57	0.90
NbAg	CH3OH	-663.41	1.44	0.21	-662.18	-0.21
		-635.11			-658.35	0.00
RuRh	COOH	-662.39	0.63	0.12	-661.89	-0.14
RuRh	CO	-651.83	0.22	0.12	-651.74	-0.67
RuRh	CHO	-655.11	0.48	0.12	-654.75	0.39
RuRh	CH2O	-658.44	0.80	0.15	-657.79	0.36
RuRh	CH2OH	-662.36	1.10	0.13	-661.39	-0.20
RuRh	CH3OH	-665.65	1.38	0.20	-664.47	0.31

Table S18. The free energy (ΔG/eV) of electrocatalytic CO_2 reduction on TM1/TM2–N@Gra to generate CH_3OH (Path3: CO_2-*COOH-*CO-*CHO-*CH_2-*CH_3 -*CH_3OH).

metal	molecules	E_0/eV	E_ZPE/eV	T*S	ΔE/eV	ΔG/eV
						▽
		-632.18	-632.18		-655.41	0.00
AlRh	COOH	-660.88	0.65	0.14	-660.36	-1.55
AlRh	CO	-649.32	0.22	0.11	-649.21	0.33
AlRh	CHO	-653.46	0.50	0.09	-653.05	-0.44
AlRh	CH2O	-656.81	0.79	0.12	-656.14	0.31
AlRh	CH3O	-661.09	1.11	0.20	-660.17	-0.63
AlRh	CH3OH	-663.56	1.45	0.21	-662.32	1.25
		-631.92			-655.16	0.00
AlPt	COOH	-659.26	0.67	0.13	-658.72	-0.17
AlPt	CO	-647.66	0.21	0.07	-647.52	0.38
AlPt	CHO	-651.74	0.52	0.07	-651.29	-0.36
AlPt	CH2O	-656.15	0.81	0.07	-655.41	-0.73
AlPt	CH3O	-659.72	1.08	0.07	-658.72	0.10
AlPt	CH3OH	-663.00	1.44	0.22	-661.78	0.34
		-630.93			-654.17	0.00
AlPd	COOH	-658.13	0.63	0.11	-657.62	-0.05
AlPd	CO	-646.86	0.22	0.07	-646.72	0.08
AlPd	CHO	-650.50	0.48	0.06	-650.08	0.03
AlPd	CH2O	-655.10	0.81	0.10	-654.39	-0.90
AlPd	CH3O	-658.97	1.11	0.19	-658.05	-0.26
AlPd	CH3OH	-662.07	1.43	0.23	-660.87	0.58
		-638.27			-661.50	0.00
VRu	COOH	-666.12	0.64	0.10	-665.59	-0.68
VRu	CO	-655.12	0.23	0.09	-654.98	-0.21
VRu	CHO	-658.70	0.48	0.12	-658.34	0.04
VRu	CH2O	-663.01	0.80	0.13	-662.33	-0.59
VRu	CH3O	-666.48	1.11	0.12	-665.49	0.24
VRu	CH3OH	-669.36	1.44	0.21	-668.13	0.76

		-634.50			-657.73	0.00
VPd	COOH	-661.69	0.64	0.15	-661.21	-0.08
VPd	CO	-650.51	0.20	0.11	-650.42	-0.03
VPd	CHO	-654.41	0.49	0.10	-654.02	-0.20
VPd	CH2O	-659.04	0.82	0.11	-658.34	-0.92
VPd	CH3O	-663.26	1.11	0.13	-662.28	-0.54
VPd	CH3OH	-665.06	1.40	0.24	-663.90	1.78
		-636.26			-659.50	0.00
CrNi	COOH	-663.14	0.64	0.11	-662.61	0.29
CrNi	CO	-652.40	0.22	0.10	-652.28	-0.49
CrNi	CHO	-655.73	0.50	0.12	-655.35	0.33
CrNi	CH2O	-659.96	0.81	0.12	-659.27	-0.52
CrNi	CH3O	-663.95	1.09	0.22	-663.08	-0.41
CrNi	CH3OH	-666.73	1.39	0.22	-665.56	0.92
		-636.98			-660.22	0.00
CrRh	COOH	-663.67	0.61	0.10	-663.17	0.45
CrRh	CO	-652.91	0.22	0.10	-652.80	-0.45
CrRh	CHO	-656.77	0.49	0.10	-656.38	-0.18
CrRh	CH2O	-661.04	0.80	0.11	-660.35	-0.57
CrRh	CH3O	-664.62	1.08	0.11	-663.65	0.09
CrRh	CH3OH	-667.44	1.39	0.25	-666.31	0.75
		-636.54			-659.78	0.00
MnRh	COOH	-664.14	0.64	0.16	-663.65	-0.47
MnRh	CO	-653.59	0.22	0.09	-653.46	-0.63
MnRh	CHO	-656.82	0.50	0.10	-656.43	0.43
MnRh	CH2O	-660.58	0.80	0.12	-659.90	-0.07
MnRh	CH3O	-663.93	1.09	0.20	-663.04	0.26
MnRh	CH3OH	-667.50	1.42	0.14	-666.23	0.21
		-637.80			-661.04	0.00
MnIr	COOH	-665.52	0.66	0.14	-665.00	-0.56
MnIr	CO	-654.89	0.21	0.13	-654.82	-0.63
MnIr	CHO	-658.18	0.52	0.10	-657.76	0.45
MnIr	CH2O	-661.65	0.82	0.07	-660.91	0.26
MnIr	CH3O	-664.98	1.09	0.20	-664.08	0.22
MnIr	CH3OH	-668.30	1.39	0.20	-667.11	0.37
		-629.93			-653.16	0.00
ZnRu	COOH	-657.95	0.65	0.14	-657.44	-0.88
ZnRu	CO	-647.09	0.23	0.09	-646.96	-0.33
ZnRu	CHO	-650.56	0.50	0.09	-650.15	0.20
ZnRu	CH2O	-654.00	0.81	0.10	-653.30	0.26
ZnRu	CH3O	-657.08	1.07	0.18	-656.19	0.51
ZnRu	CH3OH	-660.78	1.40	0.12	-659.50	0.08

		-627.68			-650.92	0.00
ZnPt	COOH	-655.04	0.65	0.13	-654.51	-0.20
ZnPt	CO	-644.29	0.23	0.09	-644.15	-0.45
ZnPt	CHO	-647.55	0.50	0.10	-647.14	0.41
ZnPt	CH2O	-650.67	0.79	0.13	-650.02	0.52
ZnPt	CH3O	-654.89	1.10	0.19	-653.98	-0.56
ZnPt	CH3OH	-658.49	1.42	0.20	-657.27	0.12
		-632.43			-655.66	0.00
NbAg	COOH	-659.42	0.65	0.15	-658.92	0.15
NbAg	CO	-648.44	0.21	0.11	-648.35	-0.25
NbAg	CHO	-651.86	0.46	0.11	-651.50	0.25
NbAg	CH2O	-656.73	0.79	0.13	-656.07	-1.17
NbAg	CH3O	-660.80	1.11	0.09	-659.78	-0.31
NbAg	CH3OH	-663.41	1.44	0.21	-662.18	1.00
		-635.11			-658.35	0.00
RuRh	COOH	-662.39	0.63	0.12	-661.89	-0.14
RuRh	CO	-651.83	0.22	0.12	-651.74	-0.67
RuRh	CHO	-655.11	0.48	0.12	-654.75	0.39
RuRh	CH2O	-658.44	0.80	0.15	-657.79	0.36
RuRh	CH3O	-662.37	1.10	0.19	-661.46	-0.27
RuRh	CH3OH	-665.65	1.38	0.20	-664.47	0.39
